# Actuation-enhanced multifunctional sensing and information recognition by magnetic artificial cilia arrays

**DOI:** 10.1073/pnas.2308301120

**Published:** 2023-10-04

**Authors:** Jie Han, Xiaoguang Dong, Zhen Yin, Shuaizhong Zhang, Meng Li, Zhiqiang Zheng, Musab Cagri Ugurlu, Weitao Jiang, Hongzhong Liu, Metin Sitti

**Affiliations:** ^a^Physical Intelligence Department, Max Planck Institute for Intelligent Systems, 70569 Stuttgart, Germany; ^b^State Key Laboratory for Manufacturing Systems Engineering, Xi’an Jiaotong University, 710054 Xi’an, China; ^c^School of Mechanical Engineering, Xi’an Jiaotong University, 710054 Xi’an, China; ^d^Department of Mechanical Engineering, Vanderbilt University, Nashville, TN 37212; ^e^Department of Control Science and Engineering, Tongji University, Shanghai 201800, China; ^f^Shanghai Research Institute for Intelligent Autonomous Systems, Shanghai 200120, China; ^g^School of Mechanical Engineering, Yanshan University, Qinhuangdao 066004, China; ^h^National Key Laboratory of Hoisting Machinery Key Technology, Yanshan University, Qinhuangdao 066004, China; ^i^Hebei Key Laboratory of Heavy Machinery Fluid Power Transmission and Control, Yanshan University, Qinhuangdao 066004, China; ^j^Institute for Biomedical Engineering, ETH Zürich, 8092 Zürich, Switzerland; ^k^School of Medicine and College of Engineering, Koç University, 34450 Istanbul, Turkey

**Keywords:** soft robot, fluidics, bioinspiration, actuation-enhanced sensing mechanism, sensor-integrated cilia

## Abstract

Artificial cilia integrating both actuation and sensing functions allow simultaneously sensing environmental properties and manipulating fluids in situ, which are promising for environment monitoring and fluidic applications. However, existing artificial cilia have limited ability to sense environmental cues in fluid flows that have versatile information encoded. This limits their potential to work in complex and dynamic fluid-filled environments. Here, we propose a generic actuation-enhanced sensing mechanism to sense complex environmental cues through the active interaction between artificial cilia and the surrounding fluidic environments. The proposed mechanism is based on fluid–cilia interaction by integrating soft robotic artificial cilia with flexible sensors. With a machine learning-based approach, complex environmental cues such as liquid viscosity, environment boundaries, and distributed fluid flows of a wide range of velocities can be sensed, which is beyond the capability of existing artificial cilia. As a proof of concept, we implement this mechanism on magnetically actuated cilia with integrated laser-induced graphene-based sensors and demonstrate sensing fluid apparent viscosity, environment boundaries, and fluid flow speed with a reconfigurable sensitivity and range. The same principle could be potentially applied to other soft robotic systems integrating other actuation and sensing modalities for diverse environmental and fluidic applications.

## Significance

The ability to sense environmental cues encoded in fluid flows could enable intelligent behaviors of artificial cilia for versatile applications such as aquatic environmental monitoring and exploration. Our proposed actuation-enhanced sensing mechanism and sensor-integrated cilium (SIC) allow for the ability of recovering environmental information such as fluid apparent viscosity, environment boundaries, and fluid flow speed with a reconfigurable sensitivity and sensing range, all by actively interacting with the fluidic environments. By incorporating the actuation-enhanced sensing mechanism, our demonstrated artificial cilia devices are promising for enabling sensing complex and dynamic cues in fluidic environments, which paves the way for developing the next generation of soft robots and devices with enhanced environmental awareness and adaptation for diverse fluidic and environmental applications.

Cilia are tiny slender structures widely existing in various biological systems (1), such as reef corals (2), *Paramecium* (3), and the human body (4–6), which have the functions of pumping and mixing fluids, swimming propulsion, and sensing fluid flows. In biological systems, motile cilia can efficiently pump fluids at low Reynolds (*Re*) numbers, while primary cilia cannot actively manipulate fluid but exhibit mechanosensory and chemosensory functions (7). Inspired by the active motions and sensing functions of biological cilia, small-scale cilia-like devices as actuators and sensors, which can manipulate fluids or sense the fluid properties in narrow spaces, show great potential in microfluidics (8, 9), biomechanics (10), biomedical engineering (11), and flow detection (12). For example, integrating flexible sensors into miniature devices (13, 14) could potentially enable physical intelligence (15) by allowing adaptive behaviors in dynamic, complex, and fluid-filled confined environments using in situ feedback of the environmental cues.

Existing artificial cilia are limited to passive sensing of simple environmental cues such as fluid flows, or active manipulation of fluid flows without any sensing capabilities, as summarized in *SI Appendix*, Table S1 (16, 17). These limitations prevent them from operating in complex fluidic environments adaptively, which requires in situ sensing-based closed-loop control, as observed in biological systems (18). Notably, the active interactions between artificial cilia and the fluidic environment have extensive information on the surrounding environment encoded in the movement of artificial cilia (19). However, due to the lack of precise deformation sensing ability, efficient signal extraction, and parameter estimation methods, it remains challenging to develop artificial cilia that are capable of both active flow manipulation and simultaneous sensing advanced environmental cues in complex and dynamic fluidic environments.

To tackle this challenge, we propose an actuation-enhanced sensing mechanism for artificial cilia by integrating simultaneous actuation and sensing capability in the cilia structures. With a machine learning-based approach, implicit environmental information is further recovered. We showcase the mechanism on an individually addressable artificial cilia array with magnetically actuated soft bodies and integrated flexible strain-gauge sensing layer using laser-induced graphene (LIG) (20, 21). The magnetic composite elastomer and conductive LIG allow programmable dynamic motions and precise in situ motion sensing in fluidic environments (gauge factor up to ~3,500, strain detection limit down to 0.01%). The proposed actuation-enhanced sensing mechanism enables simultaneously sensing critical environmental properties during fluid manipulation, such as the apparent viscosity of both Newtonian and non-Newtonian fluids, environment boundaries of different sizes and distances, and fluid flows with an adjustable speed range. Moreover, the actuation-enhanced sensing mechanism also enables 1) self-sensing of the deformation of the cilium body and 2) an adjustable stiffness-enabled tunable sensing range. Our proposed mechanism is potentially applicable to other soft actuators and flexible sensors to enhance the fluid manipulation and sensing abilities of soft robots and devices for fluidic and environmental applications.

## Results

### Mechanism of Actuation-Enhanced Sensing in Artificial Cilia.

**Fig. 1.**
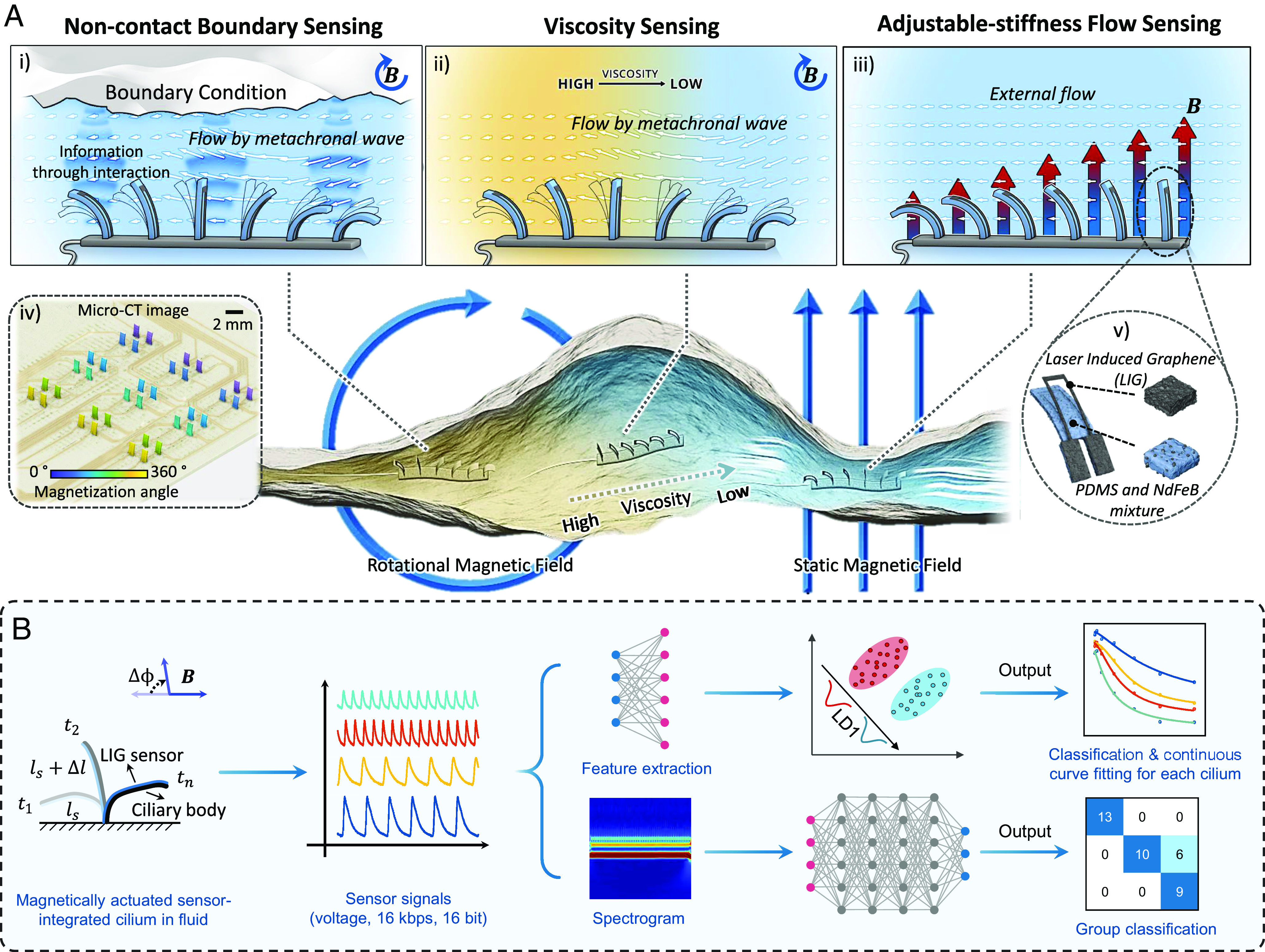
Concept of the actuation-enhanced sensing mechanism and signal analysis. (*A*) The proposed application scenarios for the actuation-enhanced sensing mechanism of the SIC array. The SIC array could sense i) the size and distance of the boundary wall, ii) apparent viscosity of the liquid environment, and iii) the fluid flow with adjustable stiffness in a confined space. iv) The microcomputational tomography (micro-CT) image of the SIC array (6×6) with integrated sensors on top of a printed circuit board (PCB) for distributed sensing of liquid environment properties. v) The zoomed-in schematic of the SIC with a layer of magnetic composite and a layer of LIG. (*B*) The signals collected from SICs in different fluid environments are encoded as standard pulse code modulation (PCM) signals. To enable continuous prediction, features are extracted from the signals in both time and frequency domains and classified using linear discriminant analysis (LDA). The feature that contributes the most to the classification is used in the calibration of SICs, which further enables the prediction of unknown environmental properties. To efficiently classify signals from a large number of SICs, the corresponding spectrograms are generated using the wavelet packet decomposition (WPD) and processed using external recognition with deep-learning models.

We propose an actuation-enhanced sensing mechanism for detecting environmental cues in fluid-filled confined spaces. As shown in Fig. 1*A*, the cilia motion can be precisely recorded by an integrated sensor, providing abundant environmental information based on the interaction between the liquid environment and the cilia. It is noteworthy that the influence of various environmental conditions on ciliary movement is spatiotemporal, which allows to estimate liquid environmental information by analyzing this previously overlooked information using machine learning techniques. As a proof of concept, we demonstrate three actuation-enhanced sensing showcases, including sensing fluid viscosity, sensing environment boundary in a noncontact-based manner, and sensing fluid flows with an adjustable sensitivity and sensing range. While our proposed actuation-enhanced sensing mechanism is generic for various flexible actuators and sensors, we implement the mechanism with magnetic artificial cilia for wireless actuation and LIG-based strain gauge sensors due to their high sensitivity. The sensor-integrated cilia array with an addressable signal acquisition circuit can convert the strain to electric signals when the cilia are actuated by external magnetic fields. As shown in Fig. 1*B*, the behavior of the SIC can be individually calibrated to achieve continuously monitoring and predicting environmental cues in fluidic environments. Furthermore, deep learning models can be applied to classify high-throughput information and identify liquid environments even in challenging and complex conditions. Additionally, these SIC arrays could be potentially deployed on miniature flexible appendages with embedded liquid metal circuits (*SI Appendix*, Fig. S1), which could adapt to the surface with a complex shape and could be potentially integrated with different miniature robots, devices, and other tools to work in confined fluidic spaces (22).

### Materials, Fabrication, and Characterization of SIC.

**Fig. 2.**
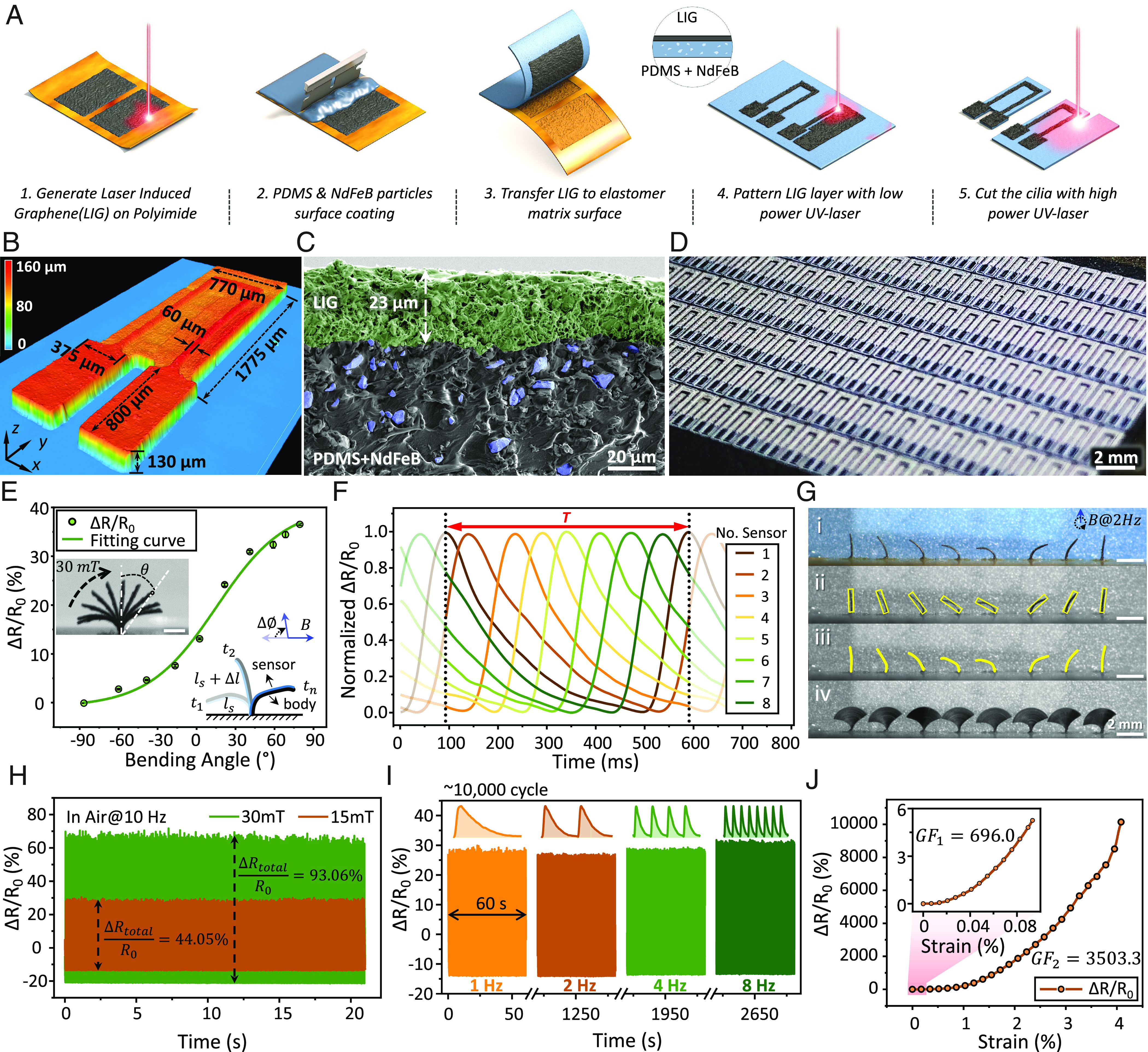
Design, fabrication, and characterization of the SIC array. (*A*) The illustration of the proposed laser-based method for fabricating the SIC array. (*B*) The 3D profile of a single sensor-integrated cilium recorded by a laser surface profiler microscope. (*C*) The SEM image (recolored) of the bilayer structure after the LIG transfer process. On top is the LIG layer (green) and on bottom is the PDMS-NdFeB composite layer. The NdFeB particle is marked in blue (average diameter of 5 μm). (Scale bar, 20 μm.) (*D*) Optical image of cilia array after the laser-based fabrication process. The dark part is the patterned LIG sensor, and the gray part is the magnetic elastomer after engraving, which holds a rough surface with Sa = 1.092 μm. (Scale bar, 2 mm.) (*E*) The data plot of relative resistance changes $\left(\Delta R/R_{0}\right)$ as a function of the cilium bending angle. The fitting curve from nonlinear regression holds R^2^ = 0.995. (Scale bar, 1 mm.) (*F*) Signals with phase difference obtained from a SIC array (1×8) with the magnetic profiles in metachronal coordination. (*G*) The optical image and the image analysis of a SIC array with metachronal waves. (Scale bar, 2 mm.) (*H*) The signal amplitude of a SIC changed with different applied magnetic field strengths in air. (*I*) Durability tests of the SIC under over 10,000 cycles at different magnetic actuation frequencies, ranging from 1 Hz to 8 Hz. The inserted subfigure shows the signal in 1 s. (*J*) The scaled resistance changes of the LIG-based sensor as a function of the strain in a tensile test. The gauge factor reaches around 700 when the applied strain is less than 0.1% and around 3,500 when the strain is in the range of 1% to 4%.

Fig. 2 illustrates the fabrication and characterization process of the proposed SIC in terms of material properties and sensing performance. In Fig. 2*A*, the laser-based massive fabrication processes are presented, including generating LIG (thickness, ~20 μm) on a polyimide (PI) tape, and transferring it onto a thin polydimethylsiloxane and neodymium iron boron (PDMS-NdFeB) sheet with a thickness of ~120 μm. To pattern the LIG sensors and cut the cilia array with a high resolution, we present an ultraviolet laser-based fabrication strategy that combines laser delamination and cutting. In general, the laser structuring method is used to remove the extra LIG for desired sensor patterns (minimum feature size, 20 ± 2 μm, shown in *SI Appendix*, Figs. S1 and S2). And the laser cutting method is used to pattern the cilia array, while a thin layer of PDMS-NdFeB mixture film (thickness, ~3 μm) is accordingly added on top for isolation. Finally, as shown in *SI Appendix*, Fig. S3, a jig-assisted method is used for programming the magnetic material with desired magnetic profiles (10, 23). The SICs are further integrated on a customized printed circuit board (PCB) with electrodes connected to the circuit using silver conductive paint, resulting in a SIC array that is capable of sensing mechanical deformation while being controlled magnetically. It is worth mentioning that the fabrication method of this magnetic SIC array and corresponding circuits can be further optimized for integrated massive production using a lithographic process, which is fully compatible with the proposed actuation-enhanced sensing mechanism (24, 25).

Fig. 2*B* and *SI Appendix*, Table S2 present the three-dimensional (3D) profile of a millimeter-scale ferromagnetic-elastic cilium (length: 1.8 mm) with an integrated LIG-based sensor. The scanning electron microscope (SEM) images (Fig. 2*C* and *SI Appendix*, Figs. S1 and S2) show the porous and continuous profile of the LIG layer, which enables a strong bonding between the LIG and magnetic elastomer (26). As the porous LIG layer works as a strain gauge (sheet resistance: 35.98 ± 0.91 Ω/square) with a high sensitivity (gauge factor, ~3,500), the micro- and nanostructures of the LIG flakes are also visualized by a high-resolution transmission electron microscopy (HRTEM) as shown in *SI Appendix*, Fig. S1. After utilizing the laser delamination and cutting strategies as described above, massive production of the proposed SIC array can be achieved (Fig. 2*D*). The SICs are inserted into the customized PCB according to their different phases after being magnetized inside a 1.8-T magnetic field. The SIC array shows a metachronal beating motion upon applying a rotating uniform magnetic field ( $B_{m}$ , 15 ~ 40 mT), as shown in *SI Appendix*, Figs. S3 and S4. This SIC array with metachronal motions can pump the glycerol and transport particle at an average speed of $\begin{array}{ccc} v_{x} & = & 0.72\;\pm \;0.07\;mm/s \end{array}$ ( $\begin{array}{ccc} B_{m} & = & 30\;\mathrm{mT} \end{array}$ , $\begin{array}{ccc} f & = & 2\;\mathrm{Hz} \end{array}$ ), as shown in *SI Appendix*, Fig. S5 and Movie S1. Though maximizing the pumping speed of our device is not the main focus in this work, we show that the proposed SIC array still exhibits comparable pumping capability when compared with other existing artificial cilia arrays, as summarized in *SI Appendix*, Table S3.

The sensitivity and stability of the integrated sensor are crucial for accurately sensing the movement and deformation of the SIC, which capture the effect of different liquid environments. We perform a systematic investigation of the response of the LIG-based sensor to mechanical deformation. Fig. 2*E* and *SI Appendix*, Fig. S6 show the resistance change as a function of the cilium bending angle at quasi-static states when varying direction of the applied magnetic field. The cilium bending angle *θ* is defined as the angle between the line connecting the cilium tip to its base and the vertical axis, with negative angles indicating leftward bending and positive angles denoting rightward bending. The initial net resistances $R_{0}$   of the integrated sensors are from 3 kΩ to 6.5 kΩ due to the errors introduced during fabrication. As the applied magnetic field ( $\begin{array}{ccc} B_{m} & = & 30\;\mathrm{mT} \end{array}$   ) rotates with an incremental angle of 20° and holds for 5 s to stabilize the cilium motion in air, the maximum $\Delta R/R_{0}$   reaches 35% after the cilium tip bending at 70° (rotation angle of the magnetic field, 90°). The normalized resistance change $\Delta R/R_{0}$   increases when increasing the bending angle of the cilium ( $R^{2}=0.995$   ). For a 1 × 8 SIC array with a magnetization phase difference of $\begin{array}{ccc} \Delta \phi & = & 45{}^{\circ} \end{array}$   , both the LIG sensor signals and the optically tracked cilia motions show a metachronal coordination when a rotating magnetic field is applied (Fig. 2 *F* and *G*, *SI Appendix*, Fig. S4, and Movie S2). The amplitude of the sensor signal depends on the strength of the applied magnetic field as shown in Fig. 2*H*. When driven at a frequency of 10 Hz in air, the $\Delta R/R_{0}$   reaches 93.06% when $\begin{array}{ccc} B_{m} & = & 30\;\mathrm{mT} \end{array}$   and 44.05% when $\begin{array}{ccc} B_{m} & = & 15\;\mathrm{mT} \end{array}$   . As shown in Fig. 2*I*, the SIC system also shows relatively good durability in a continuous cyclic test for about 10,000 cycles, where rotating magnetic fields with different actuation frequencies ( f   , 1 Hz to 8 Hz; $\begin{array}{ccc} B_{m} & = & 15\;\mathrm{mT} \end{array}$   ) are applied. It is noteworthy that while there are variations in $R_{0}$   along different SICs, the signal responses to bending angles under the same driving conditions are similar after normalization (*SI Appendix*, Fig. S7). Moreover, the reversible electrical connection in the network-like structure of the LIG allows the integrated sensor with a relatively large gauge factor of ~3,500 at 4% elongation as shown in Fig. 2*J*. In addition, the sensor exhibits a remarkable angular resolution and a rapid temporal response resolution under dynamic conditions, indicating its ability to detect rapid and delicate changes in deformation (*SI Appendix*, Fig. S6 and Movie S3). Overall, the integrated LIG-based sensor is highly reliable and sensitive for detecting the small deformation of the SIC, especially when optical imaging-based methods are limited or not available (10, 27).

### Sensing Liquid Viscosity in Fluid-Filled Environments.

With the ability of sensing deformation by the SIC, we first demonstrate sensing liquid viscosity by employing an actuation-enhanced sensing mechanism. Different from the conventional optical- or medical imaging–based method, our proposed SIC array and the sensing mechanism utilize both the known applied magnetic field and the shape information of the cilium given by the LIG sensor to achieve liquid viscosity sensing. This device and mechanism could be used for diagnosing diseases in biomedical engineering where liquid viscosity is an important physiological biomarker of biofluids inside the body (28). For example, blood viscosity is associated with cardiovascular diseases such as stroke and coronary heart disease (29), while mucus viscosity is related to gastrointestinal tract diseases, such as peptic ulcer (30).

**Fig. 3.**
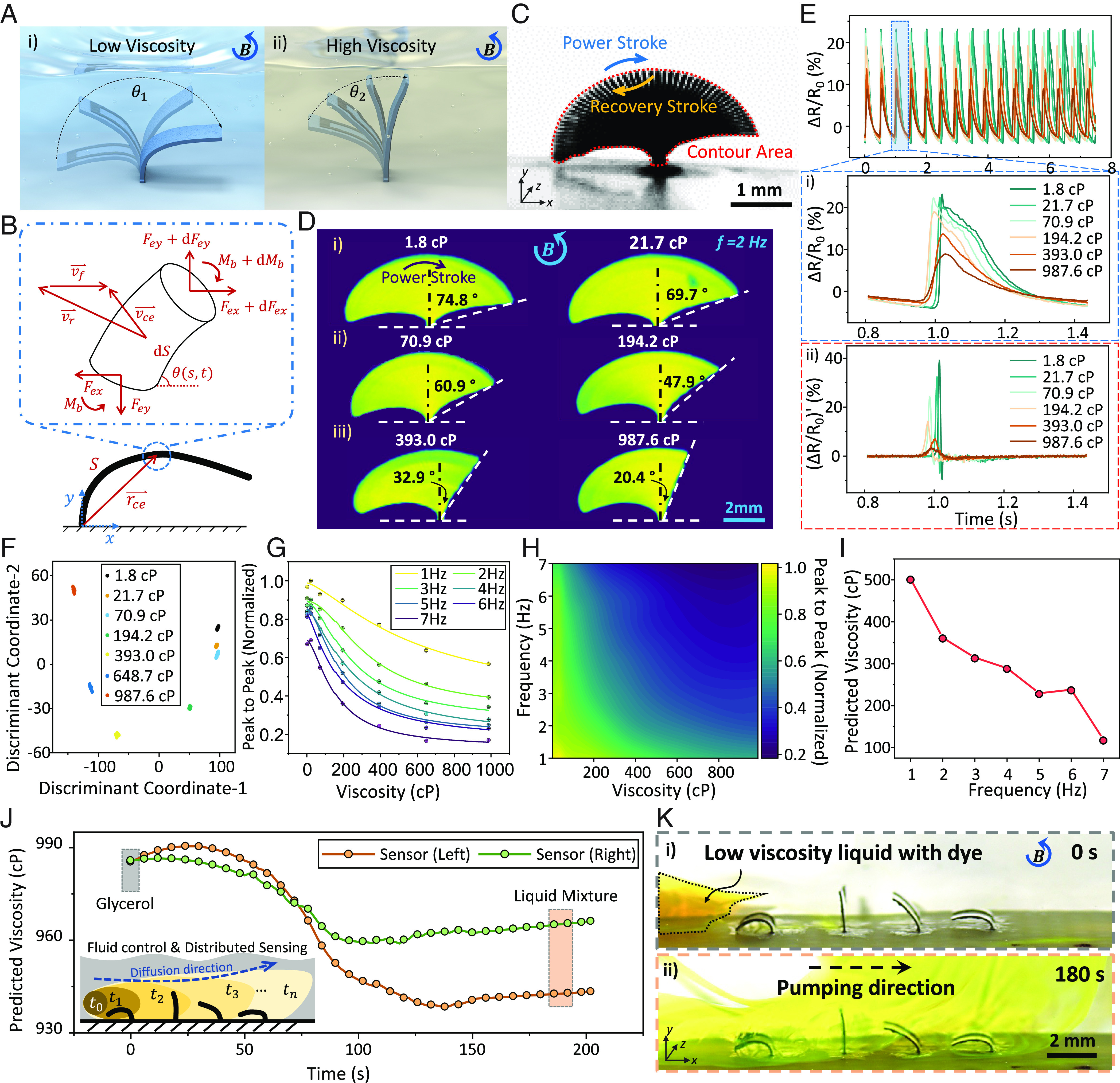
Demonstration of sensing the viscosity of a liquid environment. (*A*) Illustration of the motions of the SIC in liquid environments of different viscosities. The proposed magnetically controlled cilium holds a larger bending angle in a low-viscosity environment than that in a high-viscosity environment with the same actuation signals. (*B*) The force analysis diagram of the infinitesimal element of the SIC. The movement of SIC is influenced by the fluid drag force $F_{D}$ , magnetic force $F_{m}$ and torque τ . (*C*) Stacked optical images of the SIC in one actuation cycle. Tested in air with $\begin{array}{ccc} B_{m} & = & 15\;\mathrm{mT} \end{array}$ and $\begin{array}{ccc} f & = & 2\;\mathrm{Hz} \end{array}$ . (Scale bar, 1 mm.) (*D*) Contours of the SIC actuated in different liquid environments with various viscosities, and the color is remapped from grayscale to “viridis” colormap. $\begin{array}{ccc} B_{m} & = & 30\;\mathrm{mT} \end{array}$ ; $\begin{array}{ccc} f & = & 2\;\mathrm{Hz} \end{array}$ . (*E*) Comparison among signals of a SIC under different viscosity environments. $\begin{array}{ccc} B_{m} & = & 15\;\mathrm{mT} \end{array}$ ; $\begin{array}{ccc} f & = & 2\;\mathrm{Hz} \end{array}$ . i) shows the original signal ( $\Delta R/R_{0}$ ) and ii) shows the signals after first-order deviation. (*F*) Visualized classification result on the signal categories after dimensionality reduction with LDA. Each category corresponds to different viscosity liquid environments. The extracted features of the signals include both time- and frequency-domain. $\begin{array}{ccc} B_{m} & = & 30\;\mathrm{mT} \end{array}$ ; $\begin{array}{ccc} f & = & 2\;\mathrm{Hz} \end{array}$ . (*G* and *H*) The peak-to-peak value extracted from the signals of SIC under different actuation frequencies and different viscosity liquid environments. $\begin{array}{ccc} B_{m} & = & 30\;\mathrm{mT} \end{array}$ ; $\begin{array}{ccc} f & = & 1\;\mathrm{to}\;7\;\mathrm{Hz} \end{array}$ . (*I*) The fluid viscosities predicted by the proposed SIC at different actuation frequencies in non-Newtonian fluids (hyaluronic acid aqueous solutions, 6.3 mg/mL). (*J* and *K*) The simultaneous distributed dynamic viscosity perception and fluid manipulation when mixing low viscosity droplet (3 cP) into high viscosity environment (glycerol) by the proposed SIC array (array size: 2×4).

Fig. 3*A* schematically illustrates the mechanism of sensing liquid viscosity by monitoring the artificial cilium’s motion under various fluid drags when subject to a given rotating magnetic field. The force analysis in Fig. 3*B* explains the effect of the liquid viscosities on cilia motion by exerting different fluid drag forces. The analysis is further explained in *SI Appendix, Note S1, Fluid-Structure-Interaction Model for Magnetically Actuated Cilia* section. In summary, when subjected to a uniform rotating magnetic field B , the SIC exhibits nonreciprocal motions due to a rotational snap-through motion (31). The stacked optical images in Fig. 3*C* show the trajectory of the cilium in one actuation cycle. With a phase difference in the magnetization profile of the ciliary array, the array can simultaneously pump the fluid directionally at both low and intermediate Reynolds numbers ( $Re,10^{-2}-10^{1}$ , shown in *SI Appendix*, *Note S2* and Fig. S5 and Movie S1) while achieving the viscosity sensing function (Movie S4). As the liquid environments with different viscosities produce various viscous resistances to the ciliary motion, we can train a mapping between the fluid viscosity and the sensor outputs by quantifying the motion envelope of the ciliary motion (shown in Fig. 3*D*). We observe noticeable amplitude difference from the sensor output signals and their first-order temporal derivatives in liquids of different viscosities (Fig. 3*E* and Movie S5) under the same magnetic actuation waveforms ( $\begin{array}{ccc} f & = & 2\;\mathrm{Hz} \end{array}$ ; $\begin{array}{ccc} B_{m} & = & 15\;\mathrm{mT} \end{array}$ ). The sensor signal difference indicates different cilia bending angles in different liquids. The results from image processing also confirm the similar trend of the cilia bending angle compared with the sensor output, validating our sensing strategy (Fig. 3*D* and *SI Appendix*, Fig. S8). Moreover, by adjusting the actuation frequency, both the sensing accuracy and range can be tuned according to the contour area of the cilium beating motion, as shown in *SI Appendix*, Fig. S9.

We further identify the feature of the signals that holds the strongest correlation with liquid viscosity. We utilize the linear discriminant analysis (LDA) method (32) for feature extraction and signal classification as it requires a smaller dataset for the classification. The schematic diagram of the signal classification algorithm using LDA is given in *SI Appendix*, Fig. S10. During the training process, seven liquids with viscosities ranging from 1.8 cP to 1,000 cP are prepared by mixing water and glycerol. In Fig. 3*F* and *SI Appendix*, Fig. S11, the LDA method can successfully identify different viscosity environments with a classification accuracy of ~99% (signal feature used, less than 3) under the actuation frequency ranging from 1 Hz to 7 Hz ( $\begin{array}{ccc} B_{m} & = & 30\;\mathrm{mT} \end{array}$   ). The selected feature of the sensor signals in the time and frequency domains after Z-score normalization are defined in *SI Appendix*, Tables S4 and S5. Based on the contributions of these features in classification as presented in *SI Appendix*, Figs. S12 and S13, the peak-to-peak (P2P) value was selected for predicting the viscosity of an unknown fluidic environment. Based on the identified critical features, nonlinear regression is then employed for predicting the unknown liquid viscosities. The nonlinear regression results demonstrate the sensing performance of the SIC under different actuation frequencies as shown in Fig. 3 *G* and *H*. A frequency sweeping is necessary to identify the liquids of a wide range of viscosity. For example, a larger actuation frequency at ~7 Hz could improve the sensitivity to a low-viscosity liquid (<200 cP). Therefore, the range of the magnetic actuation frequency should be maximized for improving the prediction accuracy while considering its maximum actuation frequency before “step-out”. The maximum actuation frequencies of magnetic cilia in the literature are summarized in *SI Appendix*, Table S6 as a comparison with our SIC. It is worth noting that the sensor P2P value is consistent with the cilia beating contour area, as shown in Fig. 3*H* and *SI Appendix*, Fig. S8. This suggests that the signal feature selected via LDA has a physical indication which captures the effect of liquid viscosity on the cilia motions. Each cilium could be individually calibrated assuming that the coupling effect is negligible when neighboring cilia are sufficiently far from each other.

The proposed prediction strategy also enables sensing the viscosity of non-Newtonian fluids, which are commonly found in biological systems and difficult to sense (33, 34). We use a hyaluronic acid aqueous solution (6.3 mg/mL) as synthetic mucus (35) to mimic the shear thinning property of biological mucus to verify the ability of sensing the viscosity of non-Newtonian fluids. The prediction model is first trained on a dataset of Newtonian fluids of different viscosities by mixing glycerol and water in different mass ratios. The apparent viscosity is characterized with a rheometer (Discovery-HR3, TA Instruments) and compared with the predicted values by the SIC as shown in Fig. 3*I* and *SI Appendix*, Fig. S8. The sensed apparent viscosities decrease from 502 cP to 119 cP when the magnetic field actuation frequency increases from 1 Hz to 7 Hz, similar to the trend of the measured values by the rheometer. The proposed SIC could be potentially used for quantifying the mucus shear-thinning property and detecting its viscosity change. Please note that we assume there is no background fluid flow in these scenarios. Theoretically, it is still possible for the SICs to detect fluid viscosity even in the presence of an external background flow, assuming that the fluid drag force introduced by the external background flow $F_{f}$ is significantly smaller than that introduced by the viscous resistance of liquid $F_{v}$ due to the active deformation of the SIC. Please see *SI Appendix, Note S1* for further detailed explanation.

With the sensing ability of each SIC in an array, the SIC array could be utilized to simultaneously manipulate fluid and sense the viscosity in a distributed manner. As shown in Fig. 3 *J* and *K*, the SIC array is placed in glycerol (987.6 cP) when a small droplet of low-viscosity liquid (mixture of glycerol and water; volume ratio, 1:1; viscosity, 7.1 cP) with fluorescein sodium is added to one end of the cilia array. With the SIC array beating with metachronal coordination, the liquid droplet is mixed inside the glycerol for approximately 3 min, while the viscosity of the mixture could be monitored in the process. The integration of cilia-like mechanosensory in-flow control and distributed viscosity sensing could be potentially used for biomedical applications (36).

### Sensing Environmental Boundaries in Fluid-Filled Confined Environments.

We further demonstrate the capability of sensing the boundary of fluidic environments using our actuation-enhanced sensing mechanism. Our approach enables sensing fluidic environmental boundaries in a noncontact manner, which can potentially help prevent the damage of a device when operating in confined fluidic spaces. This function is challenging to achieve using a passive sensing method as shown in previous studies (37). Compared to traditional proximity sensors such as capacitive proximity sensors (38) that require conductive liquids or ultrasonic proximity sensors (39) that are bulky and rigid, our proposed SIC offers the ability of working in various fluidic environments, especially in complex and narrow spaces, such as integrated into microfluidic devices or medical tools operating inside the body. Several active object-sensing methods have been reported in recent studies (19, 40), but they are limited either by the optical imaging-based methods or by their bulky size and nonaddressable property. In contrast, our proposed SIC arrays are compact, soft, robust, and safe to contact with delicate structures.

**Fig. 4.**
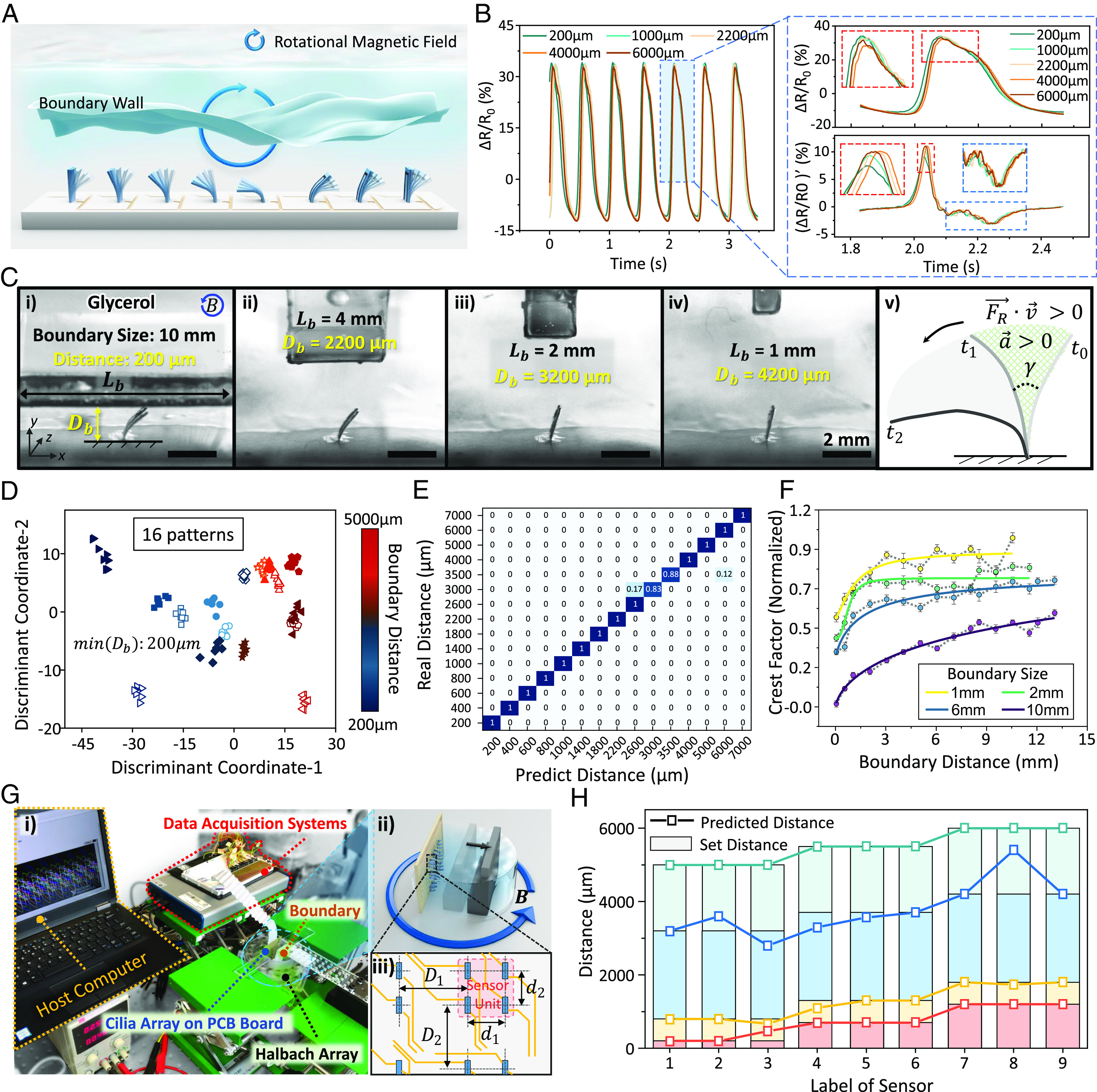
Demonstration of sensing the solid environmental boundaries. (*A*) Schematic of the distributed active sensing of boundary conditions by SIC array with metachronal coordination. (*B*) Comparison of signals of a cilium in a glycerol environment with different boundary distances. (*C*) Optical images of a 2×1 cilia array while sensing different boundaries with different distances. The marked area in (v) indicates the acceleration period of the SIC during the recovery stroke. (Scale bar, 2 mm.) (*D*) The two dimensional visualization of classification result of the different signal categories after dimensionality reduction with LDA. Each category corresponds to a different boundary distance, with a boundary size of 10 mm. The features of the signal include both time domain and frequency domain information. (*E*) The confusion matrix of classification results in boundary distance perception using the LDA method. (*F*) The changes in the Crest factor of the signals under different boundary size and distance conditions. Tested under glycerol environment with $\begin{array}{ccc} B_{m} & = & 30\;\mathrm{mT} \end{array}$ and $\begin{array}{ccc} f & = & 4\;\mathrm{Hz} \end{array}$ . (*G*) The setup for distributed boundary sensing with SIC array (array size, 6×6), in which *D*_1_ = 5.5 mm, *D*_2_ = 5.5 mm, *d*_1_ = 2.5 mm, and *d*_2_ = 2.5 mm. (*H*) Prediction results of the distributed sensing on the complex boundary. A SIC array with 3×3 sensing units is occupied, and each unit contains four cilia for high reliability. Each SIC holds its own LDA model that is trained by the test dataset.

The Fig. 4*A* depicts the schematic of our innovative distributed active sensing method for detecting environment boundaries. Our approach leverages a rotating magnetic field to drive the SIC array in a liquid environment, while the integrated sensors record the signals of cilia motion, enabling the extraction of boundary information with a no-slip boundary condition. To calibrate the SICs, a series of obstacles with varying size and distances (size ranging from 1 to 10 mm and distance ranging from 200 to 13,000 μm) are used as the training dataset (shown in Movie S6), and the recorded signals are selectively shown in Fig. 4*B*. Unlike the influence of viscosity on fluid drag force that acts on the cilium in Eq. 1 in *SI Appendix, Note S1*, which is continuous and uniform along the motion, the existence of remote objects will change the stream field (especially the stream near obstacle), and the cilium motion is perturbed mainly when approaching an obstacle, and the effect decreased dramatically when SIC tip swings away from the obstacle. The high-speed camera recordings reveal this phenomenon as the duration of the acceleration phase ( $\vec{F_{R}}\cdot \vec{v}>0$ ) in the recovery stroke is changed, which is characterized by γ in Fig. 4*C* and *SI Appendix*, Fig. S14.

Similar to the analyzing method for viscosity sensing, the LDA method is also utilized to identify the critical features of recorded signals that are sensitive to changes in boundary conditions. The classification results of the dataset with 16 different boundary distances (ranging from 200 to 5,000 μm) are presented in Fig. 4*D*, which demonstrates a high degree of intraclass aggregation and interclass distance, indicating the effectiveness of our approach. The trained model that learns the optimal linear discriminant function enables accurate classification of the signals at different boundary distances, which is a critical requirement for precise boundary detection. For the test dataset, a classification accuracy more than 97% can be achieved using only 3 features (as demonstrated in *SI Appendix*, Table S7 and Fig. S15), and the confusion matrix is shown in Fig. 4*E*.

For the prediction of unknown boundary distances using nonlinear regression, the Crest factor (41) is chosen as the critical feature of the signals after evaluating the classification quality (*SI Appendix*, Figs. S16 and S17). The Crest factor values of recorded signals under different boundary sizes were plotted against the boundary distance in Fig. 4*F*. It is observed that the Crest factor values increase when increasing boundary sizes and reducing the boundary distances (boundary size, 1 to 10 mm; boundary distance, 50 to 13,000 μm). The Crest factor also indicates that the SIC array holds a lower perception ability for further distances beyond its sensing range (>7 times of the body length) and a higher sensing quality for larger boundary conditions with less signal jitter ( $\begin{array}{ccc} R_{2} & = & 0.85 \end{array}$ for 1 mm boundary, $\begin{array}{ccc} R_{2} & = & 0.97 \end{array}$ for a 10 mm boundary). The boundary sensing ability could also be extended to multiple SICs with similar performance through individual calibration.

To further demonstrate the distributed boundary sensing ability, a 6×6 SIC array is used in the testing experiments. The pixel fusion technique is utilized where the adjacent 2×2 SIC array is treated as a single sensing unit to enhance accuracy and minimize errors, resulting in an enhanced SIC array with 3×3 sensing pixels. This data-driven approach ensures proper operation of the SIC array after calibration, leading to improved accuracy and stability of the sensing system. The schematic is shown in Fig. 4*G*. All SICs are calibrated using a training dataset with 10 different distance settings, and the signals are collected simultaneously with a multichannel data acquisition system. A 3D-printed 3-step ladder model with a 0.5-mm height difference is used as the test obstacle. Driven by a manual translation stage, four different distance settings (200 μm, 800 μm, 3,200 μm, and 5,000 μm) are tested, and the results of the predicted distance at each sensing unit are shown in Fig. 4*H*.

### Deep Learning-Assisted Recognition of Liquid Viscosity and Environmental Boundary in Confined Fluid-Filled Environments.

**Fig. 5.**
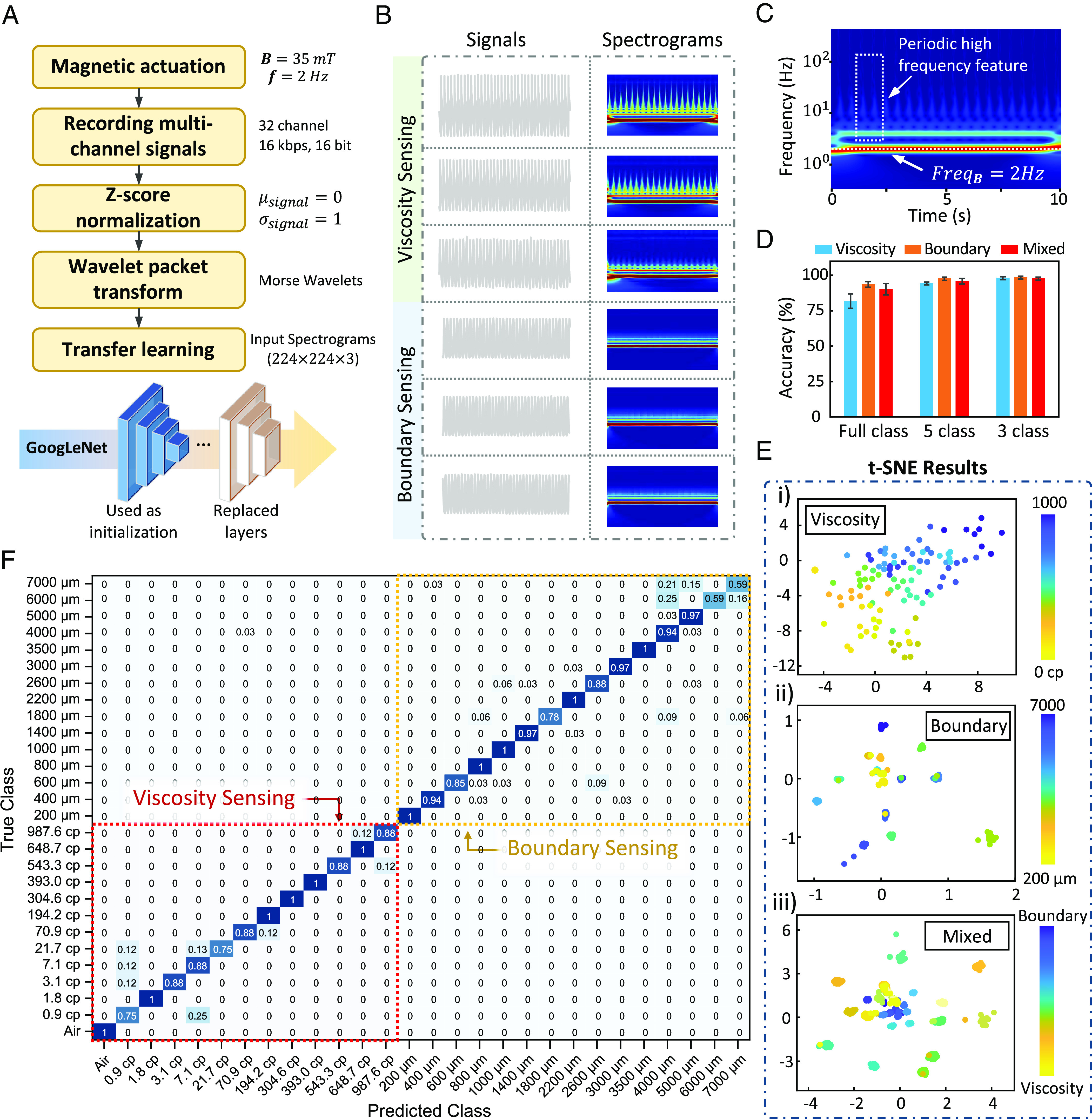
Flow chart of the signal processing and the metrics of recognition-related performance in the sensing of viscosity and boundary conditions. (*A*) The basic process of using GoogLeNet to classify the dataset of signals collected from the SIC array. (*B*) The sample signals and corresponding spectrograms of SICs under different viscosity and boundary conditions. (*C*) A spectrogram of the signal in viscosity sensing, showing periodic features in different frequencies. $\begin{array}{ccc} B_{m} & = & 30\;\mathrm{mT} \end{array}$ , $\begin{array}{ccc} f & = & 2\;\mathrm{Hz} \end{array}$ . (*D*) Recognition accuracies of the signals in the mode of viscosity sensing, boundary sensing, and mixed sensing (independent SIC samples of n = 40 used in viscosity sensing, and n = 160 in boundary sensing). The full class recognition of viscosity includes 13 sets of different viscosity conditions ranging from $10^{-2}$ cP to $10^{3}$ cP, and 15 sets of different boundary conditions ranging from 200 μm to 7,000 μm. (*E*) The results of t-distributed stochastic neighbor embedding (t-SNE) for the signals in viscosity, boundary, and mixed sensing mode. (*F*) Confusion matrix of the mixed sensing mode with changes in both viscosity and boundary (overall accuracy of 90%).

By establishing a mechanical model of the influence of the fluid environment on ciliary movement, we analyze its impact on integrated sensor signals. Based on this analysis, the LDA model–based critical feature selection and the nonlinear regression method enable high accuracy and continuous prediction of the liquid environmental properties compared to conventional classification methods. However, a more efficient calibration method such as deep neural network (DNN) is further needed for detecting the complex fluid environment in more practical applications, as the LDA models and regression equation parameters are unique for each SIC. The DNN has already shown superior performance in voice signal recognition tasks than conventional classification models in recent years (42). To balance the accuracy and efficiency in practical applications, we apply well-known neural network models that have delivered excellent performance in computer vision in our spectrogram recognition tasks via transfer learning, as shown in Fig. 5. The training parameters of the network are listed in *SI Appendix*, Table S8. We preprocess the raw signals from the various SICs by applying Z-score normalization and transforming them into logarithmic-scale spectrograms through wavelet packet transformation, and the spectrograms are used as a training dataset for transfer learning using GoogLeNet (43), shown in Fig. 5*A*. The spectrograms in Fig. 5 *B* and *C* indicate that the signals from integrated sensors reveal the motion of SICs at low-to-mid frequencies with different strengths. The signals of viscosity sensing and boundary sensing are trained and tested first separately and then mixed. The classification results and visualizations of the network are shown in Fig. 5 *D* and *E* and *SI Appendix*, Figs. S18–S24. By analyzing the sensing signals from a group of different SICs (n = 40 in viscosity sensing and n = 160 in boundary sensing), the SICs were able to recognize different environmental properties in a relatively good accuracy of 90% (Fig. 5*F* and *SI Appendix*, Fig. S24). In addition, we find that grouping data into larger categories is helpful in improving the efficiency of model training and the accuracy of recognition, and the classification accuracy increased from 90.8 to 95.2% and 98.1% when categories are grouped from 13 to 5 and 3 in viscosity sensing (Fig. 5*D* and *SI Appendix*, Figs. S19 and S23). Furthermore, the recognizable features of the signals are analyzed and explained by the gradient-weighted class activation mapping, implying that the critical recognizable features of the signals are mainly located in the low-frequency region (from actuation frequency to around 50 Hz) in both viscosity (*SI Appendix*, Figs. S19 and S20) and boundary sensing (*SI Appendix*, Figs. S21 and S22) conditions. The test dataset evaluation demonstrates that the signals collected by the SIC array in different environmental conditions exhibit the characteristics of generalization among different SICs. In specific applications that demand long-term stability, such as continuous sensing for months, periodic recalibration may be required due to the gradual degradation of LIG-based sensors or changes in the material properties of the magnetic cilia. Nevertheless, *SI Appendix*, Fig. S25 shows that when incorporating extra data into the training set, the recognition accuracy significantly improves during model training. This demonstrates that the deep-learning model can be efficiently applied to SIC arrays, where the capability of incremental learning can further enhance the recalibration process for improved performance. In addition, to further improve the stability of sensor performance and prolong the working life of SICs, the SICs can be cleaned with deionized water after usage and stored in a dry environment. The performance stability and service life of the SICs could also be improved by improving the packaging process and quality.

### Sensing Fluid Flows with Active Tunable Sensing Range and Accuracy.

**Fig. 6.**
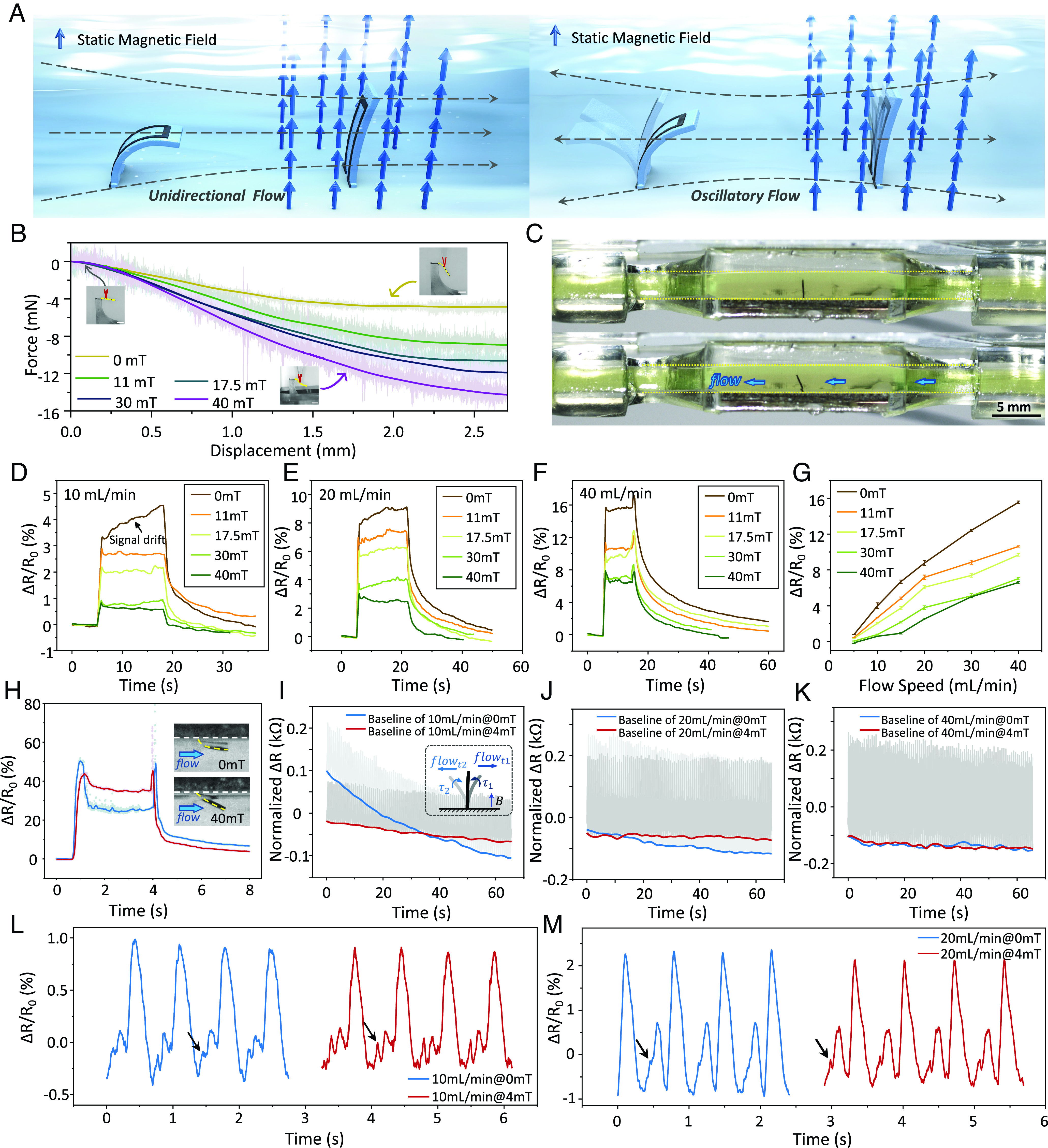
Demonstration of sensing the environmental fluid flows with reconfigurable range and sensitivity. (*A*) The illustration of magnetic field-assisted flow sensing with adjustable stiffness. (*B*) The result of the stiffness test under different magnetic fields; the test sample has the same stiffness as the SIC we demonstrated before. (*C*) Images of the SIC inside a chamber for flow sensing. The dimension of the channel cross-section is 2.6 mm × 1.5 mm ( height×width ). (Scale bar, 5 mm.) (*D*–*G*) Signals of the SIC under different magnetic field strengths when sensing flow fields with different flow rates. The flow speeds are set as 10, 20, and 40 mL/min in *D*–*F*. The magnetic field with different strengths in each flow rate situation are 0, 11, 17.5, 30, and 40 mT. The signal shows a smaller amplitude (lower sensitivity) and smaller drift under the larger magnetic field when sensing the same flow rate. (*H*) The sensing signal of a flow at 600 mL/min, and the magnetic field strength are set as 0 mT and 40 mT. The signal is distorted and has more fluctuations in the absence of a magnetic field. (*I*–*K*) The signals of the SIC under the periodic oscillating flow generated by a heartbeat pump, and the flow speeds are set as 10, 20, and 40 mL/min at 80 bpm. Compared to a nonmagnetic field situation, the signal of the sensor-integrated cilium under 4 mT shows less drift, especially when sensing low-speed flows. (*L* and *M*) The detailed signals when sensing the mimetic heartbeat flows with 10 mL/min and 20 mL/min flow rate under magnetic field of 0 mT and 4 mT. The signals of SIC under magnetic field preserve the small peaks in the sensing of oscillatory flows which is mainly due to the magnetic torque applied that help to eliminate the response hysteresis of the ciliary soft body.

To demonstrate our proposed actuation-enhanced sensing mechanism, we further propose a SIC array with a tunable stiffness by controlling external magnetic fields. Traditional strain-based sensors typically have a fixed sensing range and accuracy due to limitations in their material and design (44–46). In contrast, our device enables adaptive sensitivity and sensing range for fluid flow sensing, as well as signal drift suppression. The schematic of our proposed sensing method is shown in both unidirectional and oscillatory flows in Fig. 6*A*. As the static magnetic field is applied, the SIC with a designed magnetization profile M(s) will align to the static magnetic field B due to the magnetic torque τ . Meanwhile, the SIC will tend to recover to the initial position due to elastic stress and magnetic torque, when the SIC is deformed by the fluid drag. Due to the noncontact magnetic torque, the stiffness of the SIC can be remotely reconfigured to adjust the sensing range and sensitivity to adapt to fluid flows in different ranges. By programming M(s) of the SICs in an array, the angle of each SIC can be adjusted by applying a simple uniform magnetic field, which could potentially enhance the flow sensing for the detection of fluid flow direction.

As depicted in Fig. 6*B* and *SI Appendix*, Fig. S26, an increase in magnetic field from 0 to 40 mT resulted in the stiffness of the test sample being increased approximately three times (with a displacement of 2.8 mm, the force increased from 4.84 to 14.38 mN). The apparent stiffness of the SIC is tested under different magnetic fields using a scaled-up sample with the same stiffness and mechanical response to external magnetic field as the millimeter-scale cilium (in a bending beam situation), detailed in *SI Appendix*, Supporting Note 12. In the flow sensing experiment, the SIC with active tunable stiffness is placed in a 3D printed tube which connects to a syringe pump, as shown in Fig. 6*C* and *SI Appendix*, Fig. S27. Liquid with a similar viscosity of the human blood (viscosity: 4.26 cP) prepared by mixing glycerol and deionized water is used and visualized by fluorescein sodium. Signal differences are observed when applying magnetic fields of different magnitudes, as shown in Fig. 6 *D*–*F*. The sensor signal shows a relatively large drift at a flow speed of 10 mL/min (0.05 mm/s) with $\begin{array}{ccc} B_{m} & = & 0\;\mathrm{mT} \end{array}$   , while the drifting is eliminated when $\begin{array}{ccc} B_{m} & = & 11\;\mathrm{mT} \end{array}$   . The effects of tunable stiffness on the sensing range and sensitivity are further verified at different flow rates (5 mL/min, 20 mL/min, and 40 mL/min) with magnetic field of different magnitudes, as shown in Fig. 6*G*. The range of $\Delta R/R_{0}$   decreased from 14.73% to 6.71% under the flow of 40 mL/min when $B_{m}$   increases from 0 mT to 40 mT, significantly expanding the flow speed sensing range of the SICs. The performance of SIC for sensing a high-speed fluid flow is also tested as shown in Fig. 6*H* and Movie S7. With a flow rate of 600 mL/min, the signal drops significantly and fluctuates after reaching the peak with $\begin{array}{ccc} B_{m} & = & 0\;\mathrm{mT} \end{array}$   ( $\Delta R/R_{0}$   drops from 50.18 to 24.86%, and stabilized at 25.44% ± 1.3%), which gives false readout as the flow is already beyond its sensing limit. As shown in Fig. 6*H*, the bending angle *θ* almost reached 90°. However, the performance of SIC is greatly improved with an enlarged sensing range when $\begin{array}{ccc} B_{m} & = & 40\;\mathrm{mT} \end{array}$ compared with $\begin{array}{ccc} B_{m} & = & 0\;\mathrm{mT} \end{array}$ . The bending angle under this high-speed flow is decreased from 90° to 48°. Meanwhile, the signal drop-down is decreased from 25.32 to 9.02%, and the fluctuation is also reduced due to the increased stiffness.

It should be noted that different from the previous stiffness-changing methods, such as temperature-controlled alloys (47) and structure-controlled origami designs (48), the actuation-enhanced sensing mechanism can not only adjust the sensing range but also reduce the signal drift by exerting magnetic torque τ under dynamic loads. Tested under the periodic flow (80 beat per minute, Harvard Apparatus), the signals drifting under $\begin{array}{ccc} B_{m} & = & 0\;\mathrm{mT}\;\mathrm{and}\;4\;\mathrm{mT}\; \end{array}$ with different flow speeds are shown in Fig. 6 *I*–*K*. It is clear that the signal drift has been reduced in long-term sensing, especially at a low flow speed (Fig. 6*I*). Furthermore, the SIC under $\begin{array}{ccc} B_{m} & = & 4\;\mathrm{mT} \end{array}$ preserves and enhances the sensing ability of small dynamic changes as marked in Fig. 6 *L* and *M* under oscillatory flows, which shows the advantage of actuation-enhanced sensing on reducing the response hysteresis of the ciliary body.

## Discussion

In summary, we have developed an actuation-enhanced sensing mechanism for artificial cilia, which can accurately extract and recover the advanced cues of the surrounding fluidic environment enabled by the controlled cilia-fluid interaction and machine learning-based information recognition method. We have demonstrated three functions including sensing the apparent viscosities of Newtonian and non-Newtonian fluids (viscosity, 1.8 ~ 987.6 cP), environment boundaries with different sizes (boundary width, 1 ~ 10 mm) and distances (200 ~ 7,000 μm), and fluid flows with a reconfigurable speed range (10 ~ 600 mL/min). The ability to manipulate fluid flow while sensing the advanced properties of fluidic environment is also a major advance over previous passive sensing methods (44, 49, 50), which is demonstrated on the liquid mixing with distributed viscosity sensing (51). These demonstrations prove that the actuation-enhanced sensing mechanism in a SIC array can bring significant sensing ability beyond existing artificial cilia devices. It is noteworthy that our proposed actuation-enhanced sensing mechanism is not limited to the demonstrated magnetic actuation and strain-gauge sensing but can potentially be applied to diverse flexible sensors (13) and soft actuators (52, 53). Moreover, the types of perceived environmental properties can be potentially enlarged by varying the actuation and sensing method (54).

In the future, efforts should be made to scale down the size of the SIC for improving the spatial sensing resolution using multiple SICs. The magnetization process could also be further simplified for a larger-scale SIC array. Microfabrication methods, such as inkjet printing, two-photon polymerization 3D printing, atomic layer deposition, photolithography, and mold-based fabrication, could be potentially used to fabricate the artificial cilia at the micron length scale, as well as faster magnetization (9, 24, 55–57). It is interesting to investigate how the active adjustment of the SIC’s apparent stiffness could enhance sensing not only fluid flow speeds but also other environmental cues. As shown in *SI Appendix*, Fig. S28, by combining the static magnetic field with a rotational uniform magnetic field, the cutoff frequency for the magnetic cilia actuation could be further enlarged and the sensing ability of the SIC could be further improved. To sense the properties of biofluids such as blood and mucus, we can use our calibrated model on synthetic fluids as a baseline. The training data on biofluids and the corresponding machine learning model could further improve the accuracy in ex vivo and in vivo environments. For potential biomedical applications, the complexity of the in vivo environment may lead to a large number of uncontrollable environmental variables, such as the deformation of biological tissue and the complex structure, so that the in vivo biomedical application of the proposed SICs will require more experimental data for calibration. However, extracting a small amount of biofluid and testing them in a microfluidic device using the SIC array may be a feasible approach.

The wireless power supply and signal transmission could be potentially included inside devices with actuation-enhanced sensing capabilities. For example, a paradigm-shift wireless sensing solution based on the breakdown discharge–induced displacement current has been reported to be used for self-powered wireless sensing e-sticker (58). Moreover, a study has shown that a fully implantable wireless vascular electronics with printed soft sensors can achieve real-time in vivo monitoring of blood flows (59). In addition, ultrasound actuation could be added for remote charging and actuation, which has also been proved to achieve wireless recharging and communication simultaneously in implantable medical devices, as well as the actuation of ciliary bands (60, 61). In summary, we envision the proposed SIC array and the actuation-enhanced sensing mechanism are promising for enabling microfluidic devices and miniature robots for sensing in dynamic fluid environments. The proposed methods thus allow great potential for environment monitoring (62) and biomedical (52) applications in the future.

## Materials and Methods

### Generating LIG.

A three-step laser-based method was used to fabricate all the components of this LIG-based sensor: the generation of the LIG layer, the high-resolution patterning of the LIG-based sensor, and the cutting of mass production of cilia. For the generation of the LIG layer, a single-side PI tape (63 µm, Kapton^®^, Electron Microscopy Sciences) was attached onto a supporting glass substrate by a sacrificial layer of polyvinyl alcohol (thickness, ~10 µm; PVA, 99+% hydrolyzed, Sigma-Aldrich) in a 150-W CO_2_ laser cutter (wavelength, 10.6 µm; beam size, ~120 µm, Universal Laser Systems, PLS6-150D). The optimized parameters in a raster mode for the LIG-based strain sensor include laser power 4.1 W, laser speed 260 mm/s, laser points-per-inch 1,000, and Pulse Repetition Frequency 10.5 kHz.

### Transferring LIG.

After the nonpatterned porous LIG layer was scribed, the uncured PDMS mixed with NdFeB hard magnetic microparticles in a 1:1 weight ratio (5 µm in average diameter, Magnequench GmbH, MQP-15-7), that was prepared by planetary mixer (KK-250S, Mazerustar), was coated onto the engraved PI tape and cured at 85 °C for 6 h, and the thickness of PDMS-NdFeB layer was controlled to around 120 µm by the thickness of spacer tapes (63 µm per layer). The top surface of the mixed material was scraped by a single-edge razor blade. Because of the weak bonding between the sacrificial layer and the glass substrate, the PI tape can be easily peeled off from the glass substrate after curing. Then, the PDMS-NdFeB layer was peeled off together with the LIG layer from the PI tape, due to the strong bonding between PDMS and the LIG layer where the uncured PDMS can easily infiltrate into the porous LIG structure (26).

### PCB-Like Laser Structuring of LIG Sensor and Cilia Array.

For the fabrication of the SIC array, we developed a PCB-like fabrication method. An ultraviolet laser (UV laser) system (wavelength: 355 nm, beam size: 15 µm, Accuracy: 2 µm, LPKF ProtoLaser U3, LPKF Laser & Electronics AG) was used for the patterning of the micrometer-scale LIG sensor array and millimeter-scale cilia array. By introducing an engraving method to pattern the LIG-based sensor, the sensor with a line width of ~20 µm can be achieved. In general, the graphene layer was selectively sintered and removed by a small power laser beam (power, 2.3 W; speed, 600 mm/s) in engraving mode according to a designed line width of 60 µm (taking into account the sensitivity and stability of the sensor), and a large power laser beam (power, 3.5 W; speed, 350 mm/s) in cutting mode was used to cut the cilia array from this two-layer ferromagnetic-elastic sheet according to a designed dimension of 1.8 mm × 0.8 mm × 120 µm ( $L\times w\times t_{b}$ ). By this PCB-like fabrication method, we reduced the feature size of LIG (from 120 µm to 20 µm, according to the beam size of the CO_2_ laser) in a high resolution and convenient way (production efficiency, 5 min for 100 cilia array), which is also fully compatible with a laser cutting–based fabrication method for millimeter-scale soft robots.

### Magnetizing the Artificial Cilia Array.

We used a jig-assisted method reported in our previous work (23) for a simultaneously encoding of the desired different M(s) of a series of sensor-integrated ferromagnetic cilia using one magnetizing jig. *SI Appendix*, Fig. S3 illustrates the process and the jig we used. To obtain the desired magnetization profile, the sensor-integrated ferromagnetic cilia were placed into the jig with a series of cutout parts in designed angles and shapes. They were then magnetized by applying a large magnetizing B (magnitude, 1.8T) in the +x direction inside a vibrating sample magnetometer (EZ7 VSM, MicroSense LLC). The different phase of the cutout part corresponding to each cilium was parameterized with a relative angle to the +x direction (the direction of the magnetic field). For a cilia array with a different number of phases, the phases of the SIC were always evenly distributed between 360°.

## Supplementary Material

Appendix 01 (PDF)

Movie S1. **Flow control and particle transportation at low Reynolds number by the SIC array with magnetically controlled metachronal coordination.** This video shows our proposed sensor integrated cilia array can pump the flow to desired direction and transport the particles at low Reynolds number environment (glycerol) by changing the rotational direction of the applied magnetic field.

Movie S2. **The sensing mechanism of the proposed SIC array with metachronal coordination.** This video shows the mechanism of the sensor-integrated artificial cilia including the active motion driven by rotating magnetic field and the sensing of cilia deflection using the integrated LIG-based strain gauge sensor.

Movie S3. **Magnetically actuated motion with second-order oscillation of the SIC array during the power stroke in the air.** This video shows the movement of second-order oscillation of the sensor integrated cilia when actuated in the atmospheric environment, revealed in both the recorded video and the saved signals. The video is taken by a high-speed camera with a frame rate of 7,700 frame per second (fps).

Movie S4. **Simultaneous and distributed viscosity sensing and liquid mixing by the SIC array.** This video shows the recorded videos, raw signals and predicted viscosities of our proposed sensor integrated cilia array, which can simultaneously sense the viscosity of the liquid environment and mixing the liquids with different viscosities. The distributed sensing ability also shows advantage in detecting the dynamic changes in viscosity in localized areas.

Movie S5. **Viscosity sensing with the SIC array.** This video shows the schematic of the proposed viscosity sensing mechanism, the recorded videos and raw signals during the experiment under liquid environment with different viscosities. Both the videos and signals clearly show that the amplitude in both the power stroke and the recovery stroke is decreasing as viscosity increased.

Movie S6. **Boundary sensing with the SIC array.** This video shows the schematic of the proposed boundary sensing mechanism, the recorded videos and raw signals during the experiment with different boundary distance. The liquid we used is glycerol with viscosity of 987.6 cP.

Movie S7. **Sensing environmental fluid flows with reconfigurable sensing ranges and sensitivity.** This video shows the comparison of deformation and recorded signal of the SIC between different strength of magnetic field in same liquid flow. The cilium with larger applied magnetic field shows smaller shape deformation and more stable signal, which reveals the superiority of tuneable stiffness in sensing extreme flows. The liquid we used is simulated blood that prepared by a mixed solution of glycerol and deionized water (volume ratio of water to glycerol, 65: 44; viscosity, 4.26 cP).

## Data Availability

All study data are included in the article and/or supporting information.

## References

[1] W. Gilpin, M. S. Bull, M. Prakash, The multiscale physics of cilia and flagella. Nat. Rev. Phys. **2**, 74–88 (2020).

[2] O. H. Shapiro , Vortical ciliary flows actively enhance mass transport in reef corals. Proc. Natl. Acad. Sci. U.S.A. **111**, 13391–13396 (2014). 25192936 10.1073/pnas.1323094111 PMC4169935

[3] S. L. Tamm, Ciliary motion in Paramecium: A scanning electron microscope study. J. Cell Biol. **55**, 250 (1972). 4569410 10.1083/jcb.55.1.250 PMC2108756

[4] R. Faubel, C. Westendorf, E. Bodenschatz, G. Eichele, Cilia-based flow network in the brain ventricles. Science **353**, 176–178 (2016). 27387952 10.1126/science.aae0450

[5] R. Lyons, E. Saridogan, O. Djahanbakhch, The reproductive significance of human Fallopian tube cilia. Hum. Reprod. Update **12**, 363–372 (2006). 16565155 10.1093/humupd/dml012

[6] M. Fliegauf, T. Benzing, H. Omran, When cilia go bad: Cilia defects and ciliopathies. Nat. Rev. Mol. Cell Biol. **8**, 880–893 (2007). 17955020 10.1038/nrm2278

[7] W. A. Abou Alaiwi, S. T. Lo, S. M. Nauli, Primary cilia: Highly sophisticated biological sensors. Sensors **9**, 7003–7020 (2009). 22423203 10.3390/s90907003 PMC3290460

[8] X. Zhang, J. Guo, X. Fu, D. Zhang, Y. Zhao, Tailoring flexible arrays for artificial cilia actuators. Adv. Intell. Sys. **3**, 2000225 (2021).

[9] W. Wang , Cilia metasurfaces for electronically programmable microfluidic manipulation. Nature **605**, 681–686 (2022). 35614247 10.1038/s41586-022-04645-w

[10] X. Dong , Bioinspired cilia arrays with programmable nonreciprocal motion and metachronal coordination. Sci. Adv. **6**, eabc9323 (2020). 33158868 10.1126/sciadv.abc9323 PMC7673722

[11] T. U. Islam , Microscopic artificial cilia-a review. Lab on a Chip **22**, 1650–1679 (2022). 35403636 10.1039/d1lc01168e PMC9063641

[12] K. Rajasekaran, H. D. Bae, S. Bergbreiter, M. Yu, Flow separation sensing on airfoil using a 3D printed biomimetic artificial hair sensor. Bioinspiration Biomimetics **17**, 046003 (2022). 10.1088/1748-3190/ac61e9 35349985

[13] B. Shih , Electronic skins and machine learning for intelligent soft robots. Sci. Rob. **5**, eaaz9239 (2020). 10.1126/scirobotics.aaz9239 33022628

[14] B. Rivkin , Shape-controlled flexible microelectronics facilitated by integrated sensors and conductive polymer actuators. Adv. Intell. Sys. **3**, 2000238 (2021).

[15] M. Sitti, Physical intelligence as a new paradigm. Extreme Mech. Lett. **46**, 101340 (2021). 35475112 PMC7612657

[16] A. Alfadhel, J. Kosel, Magnetic nanocomposite cilia tactile sensor. Adv. Mater. **27**, 7888–7892 (2015). 26487397 10.1002/adma.201504015

[17] M. Asadnia , From biological cilia to artificial flow sensors: Biomimetic soft polymer nanosensors with high sensing performance. Sci. Rep. **6**, 1–13 (2016). 27622466 10.1038/srep32955 PMC5020657

[18] A. S. Shah, Y. Ben-Shahar, T. O. Moninger, J. N. Kline, M. J. Welsh, Motile cilia of human airway epithelia are chemosensory. Science **325**, 1131–1134 (2009). 19628819 10.1126/science.1173869 PMC2894709

[19] E. Judd, G. Soter, J. Rossiter, H. Hauscr, “Sensing through the body-non-contact object localisation using morphological computation” in 2019 2nd IEEE International Conference on Soft Robotics (RoboSoft) (IEEE, Seoul, South Korea, 2019), pp. 558–563.

[20] M. G. Stanford , High-resolution laser-induced graphene. Flexible electronics beyond the visible limit. ACS Appl. Mater. Interfaces **12**, 10902–10907 (2020). 32039573 10.1021/acsami.0c01377

[21] D. X. Luong , Laser-induced graphene composites as multifunctional surfaces. ACS nano **13**, 2579–2586 (2019). 30730702 10.1021/acsnano.8b09626

[22] Z. Xue , Assembly of complex 3D structures and electronics on curved surfaces. Sci. Adv. **8**, eabm6922 (2022). 35947653 10.1126/sciadv.abm6922 PMC9365271

[23] G. Z. Lum , Shape-programmable magnetic soft matter. Proc. Natl. Acad. Sci. U.S.A. **113**, E6007–E6015 (2016). 27671658 10.1073/pnas.1608193113 PMC5068264

[24] S. Hanasoge, M. Ballard, P. J. Hesketh, A. Alexeev, Asymmetric motion of magnetically actuated artificial cilia. Lab on a Chip **17**, 3138–3145 (2017). 28805871 10.1039/c7lc00556c

[25] M. Tyagi, J. Pan, E. W. Jager, Novel fabrication of soft microactuators with morphological computing using soft lithography. Microsys. Nanoeng. **5**, 44 (2019). 10.1038/s41378-019-0092-z PMC6799821 31636933

[26] Z. Zheng , Electrodeposited superhydrophilic-superhydrophobic composites for untethered multi-stimuli-responsive soft millirobots. Adv. Sci. **10**, e2302409 (2023). 10.1002/advs.202302409 PMC10427389 37288527

[27] Z. Ren , Soft-robotic ciliated epidermis for reconfigurable coordinated fluid manipulation. Sci. Adv. **8**, eabq2345 (2022). 36026449 10.1126/sciadv.abq2345 PMC9417179

[28] C. Wang, Y. Wu, X. Dong, M. Armacki, M. Sitti, In situ sensing physiological properties of biological tissues using wireless miniature soft robots. Sci. Adv. **9**, eadg3988 (2023). 37285426 10.1126/sciadv.adg3988 PMC7614673

[29] S. A. Peters, M. Woodward, A. Rumley, H. D. Tunstall-Pedoe, G. D. Lowe, Plasma and blood viscosity in the prediction of cardiovascular disease and mortality in the Scottish Heart Health Extended Cohort Study. Eur. J. Prevent. Cardiol. **24**, 161–167 (2017). 10.1177/2047487316672004 27798361

[30] F. Younan, J. Pearson, A. Allen, C. Venables, Changes in the structure of the mucous gel on the mucosal surface of the stomach in association with peptic ulcer disease. Gastroenterology **82**, 827–831 (1982). 7060905

[31] S. Khaderi, J. Den Toonder, P. Onck, Microfluidic propulsion by the metachronal beating of magnetic artificial cilia: A numerical analysis. J. Fluid Mech. **688**, 44–65 (2011).

[32] A. Tharwat, T. Gaber, A. Ibrahim, A. E. Hassanien, Linear discriminant analysis: A detailed tutorial. AI Commun. **30**, 169–190 (2017).

[33] A. Mustafa , A micropillar-based microfluidic viscometer for Newtonian and non-Newtonian fluids. Anal.Chim. Acta **1135**, 107–115 (2020). 33070846 10.1016/j.aca.2020.07.039

[34] A. Agoston, F. Keplinger, B. Jakoby, Evaluation of a vibrating micromachined cantilever sensor for measuring the viscosity of complex organic liquids. Sens. Actuators A **123**, 82–86 (2005).

[35] Z. Ren , Soft-bodied adaptive multimodal locomotion strategies in fluid-filled confined spaces. Sci. Adv. **7**, eabh2022 (2021). 34193416 10.1126/sciadv.abh2022 PMC8245043

[36] C. Atuma, V. Strugala, A. Allen, L. Holm, The adherent gastrointestinal mucus gel layer: Thickness and physical state in vivo. Am. J. Physiol. Gastrointest. Liver Physiol. **280**, G922–G929 (2001). 11292601 10.1152/ajpgi.2001.280.5.G922

[37] R. Bouffanais, G. D. Weymouth, D. K. Yue, Hydrodynamic object recognition using pressure sensing. Proc. R. Soc. A **467**, 19–38 (2011).

[38] Y. Ye , A review on applications of capacitive displacement sensing for capacitive proximity sensor. IEEE Access **8**, 45325–45342 (2020).

[39] S. D. Min , Noncontact respiration rate measurement system using an ultrasonic proximity sensor. IEEE Sensors J. **10**, 1732–1739 (2010).

[40] Z. Yu , Bioinspired, multifunctional, active whisker sensors for tactile sensing of mobile robots. IEEE Rob. Autom Lett. **7**, 9565–9572 (2022).

[41] C. Pachaud, R. Salvetat, C. Fray, Crest factor and kurtosis contributions to identify defects inducing periodical impulsive forces. Mech. Sys. Signal Process. **11**, 903–916 (1997).

[42] Q. Yang , Mixed-modality speech recognition and interaction using a wearable artificial throat. Nat. Mach. Intell. **5**, 169–180 (2023).

[43] C. Szegedy , “Going deeper with convolutions” in Proceedings of the IEEE Conference on Computer Vision and Pattern Recognition (IEEE, Boston, MA, USA, 2015), pp. 1–9.

[44] F. Ejeian , Design and applications of MEMS flow sensors: A review. Sens. Actuators A **295**, 483–502 (2019).

[45] A. M. Kamat, X. Zheng, B. Jayawardhana, A. G. P. Kottapalli, Bioinspired PDMS-graphene cantilever flow sensors using 3D printing and replica moulding. Nanotechnology **32**, 095501 (2020). 10.1088/1361-6528/abcc96 33217747

[46] K. Kwon , A battery-less wireless implant for the continuous monitoring of vascular pressure, flow rate and temperature. Nat. Biomed. Eng., 1–14 (2023). 10.1038/s41551-023-01022-4. 37037964

[47] H. Liu , Shape-programmable, deformation-locking, and self-sensing artificial muscle based on liquid crystal elastomer and low–melting point alloy. Sci. Adv. **8**, eabn5722 (2022). 35584225 10.1126/sciadv.abn5722 PMC9116885

[48] Z. Zhai, Y. Wang, K. Lin, L. Wu, H. Jiang, In situ stiffness manipulation using elegant curved origami. Sci. Adv. **6**, eabe2000 (2020). 33208377 10.1126/sciadv.abe2000 PMC7673814

[49] A. G. P. Kottapalli, M. Asadnia, J. Miao, M. Triantafyllou, Touch at a distance sensing: Lateral-line inspired MEMS flow sensors. Bioinspiration Biomimetics **9**, 046011 (2014). 25378298 10.1088/1748-3182/9/4/046011

[50] S. Ghosh, A. Sood, N. Kumar, Carbon nanotube flow sensors. Science **299**, 1042–1044 (2003). 12532025 10.1126/science.1079080

[51] J. M. den Toonder, P. R. Onck, Artificial Cilia (Royal Society of Chemistry, 2013), **vol. 30**.

[52] L. Hines, K. Petersen, G. Z. Lum, M. Sitti, Soft actuators for small-scale robotics. Adv. Mater. **29**, 1603483 (2017). 10.1002/adma.201603483 28032926

[53] M. Li, A. Pal, A. Aghakhani, A. Pena-Francesch, M. Sitti, Soft actuators for real-world applications. Nat. Rev. Mater. **7**, 235–249 (2022). 35474944 10.1038/s41578-021-00389-7 PMC7612659

[54] Y. Hao , A review of smart materials for the boost of soft actuators, soft sensors, and robotics applications. Chin. J. Mech. Eng. **35**, 1–16 (2022).

[55] C. L. Van Oosten, C. W. Bastiaansen, D. J. Broer, Printed artificial cilia from liquid-crystal network actuators modularly driven by light. Nat. Mater. **8**, 677–682 (2009). 19561599 10.1038/nmat2487

[56] H. Gu , Magnetic cilia carpets with programmable metachronal waves. Nat. Commun. **11**, 2637 (2020). 32457457 10.1038/s41467-020-16458-4 PMC7250860

[57] S. Zhang , 3D-printed micrometer-scale wireless magnetic cilia with metachronal programmability. Sci. Adv. **9**, eadf9462 (2023). 36947622 10.1126/sciadv.adf9462 PMC7614626

[58] G.-Z. Yang , The grand challenges of Science Robotics. Sci. Rob. **3**, eaar7650 (2018). 10.1126/scirobotics.aar7650 33141701

[59] R. Herbert, H.-R. Lim, B. Rigo, W.-H. Yeo, Fully implantable wireless batteryless vascular electronics with printed soft sensors for multiplex sensing of hemodynamics. Sci. Adv. **8**, eabm1175 (2022). 35544557 10.1126/sciadv.abm1175 PMC9094660

[60] P. Jin , A flexible, stretchable system for simultaneous acoustic energy transfer and communication. Sci. Adv. **7**, eabg2507 (2021). 34586839 10.1126/sciadv.abg2507 PMC8480923

[61] C. Dillinger, N. Nama, D. Ahmed, Ultrasound-activated ciliary bands for microrobotic systems inspired by starfish. Nat. Commun. **12**, 6455 (2021). 34753910 10.1038/s41467-021-26607-y PMC8578555

[62] R. Baines , Multi-environment robotic transitions through adaptive morphogenesis. Nature **610**, 283–289 (2022). 36224418 10.1038/s41586-022-05188-w

