# Supplementary material for: Actuation-enhanced multifunctional sensing and information recognition by magnetic artificial cilia arrays
Source: Proc Natl Acad Sci U S A. 2023 Oct 4;120(42):e2308301120. doi: 10.1073/pnas.2308301120 (PMC10589697; doi:10.1073/pnas.2308301120)
Supplement: Supplementary file 1 — Appendix 01 (PDF) [file pnas.2308301120.sapp.pdf]

PNAS

Supporting Information for

Actuation-Enhanced Multifunctional Sensing and Information Recognition by Magnetic Artificial Cilia Arrays

Jie Han[1,3,4#], Xiaoguang Dong[2#,*], Zhen Yin[1,5,6], Shuaizhong Zhang[1,7,8,9], Meng Li[1], Zhiqiang Zheng[1], Musab Cagri Ugurlu[1], Weitao Jiang[3,4], Hongzhong Liu[3,4], Metin Sitti[1,10,11*]

[1] Physical Intelligence Department, Max Planck Institute for Intelligent Systems, 70569 Stuttgart, Germany

[2] Department of Mechanical Engineering, Vanderbilt University, Nashville, TN 37212, USA

[3] State Key Laboratory for Manufacturing Systems Engineering, Xi'an Jiaotong University, Xi'an 710054, China

[4] School of Mechanical Engineering, Xi'an Jiaotong University, Xi'an 710054, China

[5] Department of Control Science and Engineering, Tongji University, Shanghai 201800, China

[6] Shanghai Research Institute for Intelligent Autonomous Systems, Shanghai 200120, China

[7] School of Mechanical Engineering, Yanshan University, Qinhuangdao 066004, China

[8] National Key Laboratory of Hoisting Machinery Key Technology, Yanshan University, Qinhuangdao 066004, China

[9] Hebei Key Laboratory of Heavy Machinery Fluid Power Transmission and Control, Yanshan University, Qinhuangdao 066004, China

[10] Institute for Biomedical Engineering, ETH Zürich, 8092 Zürich, Switzerland

[11] School of Medicine and College of Engineering, Koç University, 34450 Istanbul, Turkey

[#] These authors contributed equally to this work as co-first authors

* Corresponding authors: Xiaoguang Dong, Metin Sitti; xiaoguang.dong@vanderbilt.edu, sitti@is.mpg.de

This PDF file includes:

- Supporting text
- Figures S1 to S30
- Tables S1 to S8
- Legends for Movies S1 to S7
- SI References

Other supporting materials for this manuscript include the following:

- Movies S1 to S7

Supporting Information Text

Supporting Note 1

Fluid-structure-interaction model for the magnetically actuated cilia. To describe the mechanical behaviors of a magnetic cantilever beam (our proposed SIC) swinging in (i) an open fluid space with fluid velocity $\boldsymbol{v}_f = \boldsymbol{v}_f(y)$ and (ii) a fluid space with obstacles so that $\boldsymbol{v}_f = \boldsymbol{v}_f(x, y)$, under the influence of the external magnetic field $\mathbf{B}$ (shown in **fig. S31**). While our theoretical model works for situations with water flow, the flow rate is set to 0 ($\mathbf{v}_f = 0$) to focus on the influence of viscosity and boundary obstacles. The SIC receives external influences from fluid and magnetic field, and the fluid drag force $\boldsymbol{F_D}$ that covers both pressure and skin friction is expressed with the drag equation ($Re < 1$), which is given by

$$\boldsymbol{F}_D = \frac{v_r}{|v_r|}\int_A \frac{1}{2}\rho_f|\boldsymbol{v}_r|^2 C_d dA = \frac{1}{2}\rho_f|\boldsymbol{v}_r|\boldsymbol{v}_r C_d A_{ref}, \quad (1)$$

where $\boldsymbol{v}_r$ is the relative velocity between the element and fluid, ρ_f is the fluid density, C_d is the dimensionless drag coefficient assumed to be uniform across the cilium, and A_{ref} is the reference area of the element, shown in **Fig. 3B**.

The relative velocity can be expressed as:

$$\boldsymbol{v}_r = \boldsymbol{v}_{ce} - \boldsymbol{v}_f, \quad (2)$$

where $\mathbf{v}_{ce}$ is the velocity of the cilium element; $\mathbf{v}_f$ is the fluid velocity. $\mathbf{v}_{ce}$ can be expressed as:

$$\boldsymbol{v}_{ce} = \boldsymbol{v}_{ce}(s, t) = \frac{\partial \boldsymbol{r}_{ce}}{\partial t}, \quad (3)$$

where $\boldsymbol{r}_{ce} = \left[r_x, r_y, 0\right]^T$ is the Cartesian position vector of the element. Hence, we have

$$\boldsymbol{v}_r = \frac{\partial \boldsymbol{r}_{ce}}{\partial t} - \boldsymbol{v}_f. \quad (4)$$

As shown in Fig. 3B, r_{ce} can be expressed as a function of s and t:

$$\boldsymbol{r}_{ce} = \begin{pmatrix} r_x \\ r_y \\ 0 \end{pmatrix} = \begin{pmatrix} \int_0^s cos\theta(\xi, t)d\xi \\ \int_0^s sin\theta(\xi, t)d\xi \\ 0 \end{pmatrix}. \quad (5)$$

The reference area A_{ref} is usually defined as the orthographic projection of the object on a plane perpendicular to the direction of relative motion. Therefore, for the element ds, the reference area can be obtained throught the following set of equations:

$$\begin{cases} \boldsymbol{v}_r \cdot \boldsymbol{b} = 0 \\ A_{ref} = \omega \frac{\boldsymbol{n}_s \cdot \boldsymbol{b}}{|\boldsymbol{n}_s| \cdot |\boldsymbol{b}|} ds \end{cases}, \tag{6}$$

where $\mathbf{b}$ is an in-plane vector perpendicular to the relative motion, and $\mathbf{n}_s = [\cos\theta, \sin\theta, 0]^T$ is the unit vector of the element orientation.

Determining the value of C_d is generally difficult. At low Reynolds number, the drag coefficient is asymptotically proportional to Re^{-1}. Here we use an approximation used by Dong et al.(1),

$$C_d = -1.5 \ln(Re) + 7. \tag{7}$$

Let cilium with ω as the characteristic dimension, Reynolds number is:

$$Re = \frac{\rho_f v_r \omega}{\mu(\dot{\gamma})} = \left| \frac{\partial}{\partial t} \begin{pmatrix} \int_0^s \cos\theta(\xi, t) d\xi \\ \int_0^s \sin\theta(\xi, t) d\xi \\ 0 \end{pmatrix} - \boldsymbol{v}_f \right|, \tag{8}$$

where μ is the apparent viscosity of the fluid, and μ is a function of shear rate $\dot{\gamma}$ for non-Newtonian fluid (such as shear-thinning fluid).

Substitute Equation (6), Equation (7) and Equation (8) into Equation (1), we have the drag equation as a function of s and t.

For the element ds, the net magnetic moment is:

$$\boldsymbol{m} = \boldsymbol{R}_\theta \boldsymbol{M} dV = \boldsymbol{R}_\theta \boldsymbol{M} \omega t_c ds, \tag{9}$$

where $\mathbf{M} = \mathbf{M}(s)$ is the magnetization profile of the cilium,

$$\boldsymbol{M}(s) = \begin{pmatrix} M \cos\phi(s) \\ M \sin\phi(s) \\ 0 \end{pmatrix}, \tag{10}$$

where $\phi(s) = \phi(0) + s[\phi(0) - \phi(L)]/L$, and $\mathbf{R}_\theta$is the rotation matrix given by,

$$\mathbf{R}_\theta = \begin{pmatrix} \cos\theta & -\sin\theta & 0 \\ \sin\theta & \cos\theta & 0 \\ 0 & 0 & 1 \end{pmatrix}. \tag{11}$$

The magnetic flux density of the external magnetic field is

$$\boldsymbol{B} = \begin{pmatrix} B \cos(\omega t) \\ B \sin(\omega t) \\ 0 \end{pmatrix}, \tag{12}$$

where ω is the rotational angular velocity of the magnetic field.

The magnetic force is given by

$$\boldsymbol{F}_m = \nabla(\boldsymbol{m} \cdot \boldsymbol{B}) = \begin{pmatrix} \frac{\partial(\boldsymbol{m}\cdot\boldsymbol{B})}{\partial x} \\ \frac{\partial(\boldsymbol{m}\cdot\boldsymbol{B})}{\partial y} \\ 0 \end{pmatrix}, \tag{13}$$

where

$$\begin{pmatrix} \frac{\partial x}{\partial s} = cos\,\theta \\ \frac{\partial y}{\partial s} = sin\,\theta \\ 0 \end{pmatrix}. \tag{14}$$

The magnetic torque is given by,

$$\boldsymbol{\tau}_m = \boldsymbol{m} \times \boldsymbol{B}. \tag{15}$$

In the end, all the forces and torques have been expressed by s, t and $\theta(s,t)$.

The equilibrium is achieved by fluid drag, magnetic torque and force, and cilium deformation. As illustrated in **Figure. 3B**, force and moment balance of the infinitesimal element ds can be established. Firstly, moment on the cilium is balanced by beam bending (internal forces and bending moment), magnetic moment, and the moment induced by drag forces (later eliminated as it is a second-order infinitesimal).

$$\boldsymbol{m} \times \boldsymbol{B} + d\boldsymbol{s} \times \left(\boldsymbol{F}_{ey} + d\boldsymbol{F}_{ey}\right) + d\boldsymbol{s} \times (\boldsymbol{F}_{ex} + d\boldsymbol{F}_{ex}) + \frac{1}{2} d\boldsymbol{s} \times \boldsymbol{F}_D + d\boldsymbol{M}_b = 0, \tag{16}$$

where $\boldsymbol{F}_{ex}$ and $\boldsymbol{F}_{ey}$ are the internal forces of the cilium (in global xx and in yy direction). $\boldsymbol{F}_{ex} = [F_{ex}, 0,0]^T$ and $\boldsymbol{F}_{ey} = \left[0, F_{ey}, 0\right]^T$ (shown in **Fig. 3B**).

Eliminating second-order infinitesimal quantities and combing with Euler-Bernoulli beam theory, Equation (16) becomes:

$$\boldsymbol{m} \times \boldsymbol{B} + d\boldsymbol{s} \times \boldsymbol{F}_{ey} + d\boldsymbol{s} \times \boldsymbol{F}_{ex} = -d\boldsymbol{M}_b = -EI\frac{\partial^2\theta}{\partial s^2}\boldsymbol{n}_z. \tag{17}$$

The above moment balancing equation can be written into a scalar mode ($z - \mathrm{direction}$):

$$\boldsymbol{n}_z \cdot (\boldsymbol{m} \times \boldsymbol{B}) + F_{ey}\,cos\,\theta\,ds + F_{ex}\,sin\,\theta\,ds = -EI\frac{\partial^2\theta}{\partial s^2}. \tag{18}$$

where E is the Young's modulus of cilium materials and I is the second moment of area.

Secondly, force on the element is balanced by magnetic force (negligible in a uniform magnetic field), fluid drag force and cilium internal forces. Let the element internal forces $\boldsymbol{F}_{ei} = [F_{ex}, F_{ey}, 0]^T$. The force balancing equation:

$$d\boldsymbol{F}_{ei} + \nabla(\boldsymbol{m} \cdot \boldsymbol{B}) + \boldsymbol{F}_D = 0. \tag{19}$$

For the initial conditions:

$$\theta(s,0) = \theta_0, \frac{\partial\theta}{\partial s}(s,0) = 0, F_x(s,0) = \begin{pmatrix} F_x(s,0) \\ F_y(s,0) \\ 0 \end{pmatrix} = \begin{pmatrix} 0 \\ 0 \\ 0 \end{pmatrix}. \tag{20}$$

The boundary conditions are given by,

$$\theta(0,t) = \theta_0, \frac{\partial\theta}{\partial s}(L,t) = 0, F_x(L,t) = \begin{pmatrix} F_x(L,t) \\ F_y(L,t) \\ 0 \end{pmatrix} = \begin{pmatrix} 0 \\ 0 \\ 0 \end{pmatrix}. \tag{21}$$

With this model, we can understand the actuation-enhanced sensing mechanism for measuring fluid viscosity and flow by applying a magnetic field at different magnitudes and frequencies. For remote objects in the fluid environment, the stream field will be influenced by the boundary, and thus will alter the local fluid velocity and relative velocity $\boldsymbol{\tau}_m$. Therefore, the cilium can sense the distance and size of the object by essentially sensing the distribution of stream field. To know how the stream field is changed by the object can be further computed numerically.

For the situation with external background flow, the fluid drag introduced by external background flow $\boldsymbol{F}_f$ and the fluid drag introduced by liquid viscosity $\boldsymbol{F}_v$ are working in different modes to influence the movement of SIC, while $\boldsymbol{F}_f$ is in the direction of the flow and $\boldsymbol{F}_v$ is collinear with the velocity direction of the cilium element. Once $\boldsymbol{F}_f$ is steady and not the dominant force over $\boldsymbol{F}_m = \nabla(\boldsymbol{m} \cdot \boldsymbol{B})$ and $\boldsymbol{F}_v$, it would be possible for the proposed SIC array to detect the fluid viscosity with the existence of external background flow.

Supporting Note 2

The calculation of Reynolds number in different conditions. Fluid pumping motion by the proposed SICs entail typical length-scales L of $\sim 10^{-3}$ m and velocities u of $\sim 10^{-2}\ \mathrm{ms^{-1}}$, with typical fluid densities around $\rho = \sim 10^{3}\ \mathrm{kgm^{-3}}$ and fluid viscosities μ of $\sim(10^{-3} - 10^{0})\ \mathrm{Pa \cdot s}$ (a series of liquid environment with viscosity ranging from 1.8 cp to 987 cp). As $Re = \rho u L/\mu$, the Reynolds number of the flow we consider is $Re = 10^{-2} - 10^{1}$, which indicates that the cilia experienced the switching of dominant force from viscous forces to inertial forces in the viscosity sensing mode, while the viscous force is greater than the inertial force in the boundary sensing mode ($Re = 10^{-2} \ll 1$), and $Re > 10$ in the flow sensing mode.

Supporting Note 3

Fabrication of the PCB. The customized PCB for the data collection system was designed by a free online electronic design automation (EDA) software (Lichuang EDA, China) and fabricated by the UV laser system (LPKF ProtoLaser U3, LPKF Laser & Electronics AG). Except for the circuit part, an array of through holes on the PCB was also designed for inserting the two electrodes of each cilium, which was connected to the data acquisition system for signal collection. The PCB we used was flame retardant (FR4) laminated circuit board (0.8 mm, copper 35 μm, C.I.F., France). For connecting the PCB with the DAQ device, the foldable flat flexible cable (Grid, 0.5 mm, WR-FFC, Wurth Elektronik) and flexible printed circuit (FPC) connector socket (pitch, 0.5 mm, Easy-On, Molex) with different numbers of pins were used. All the PCB designs are shown in **fig. S27, S29**.

Supporting Note 4

Fabrication of soft substrates with embedded liquid metal circuits. A PDMS film with a thickness of 600 μm was used as the soft substrate, and fumed colloidal silica particles (0.1 wt%; average diameter, 0.2 to 0.3 μm; Sigma-Aldrich) were added as a dye for high-efficiency laser processing. First, a patterned precursory Cr/Au (thickness: 10 nm and 200 nm) layer was sputtered (EM ACE600, Leica) on top of the PDMS film using a thin PI film mask (thickness, 20 μm; fabricated by laser cutting). Second, the PDMS film was processed by a UV laser in both cutting and raster mode (LPKF U3, LPKF Laser & Electronics AG) to have the desired shape with holes in the corresponding positions to accommodate the cilia electrodes. After bonding the cilia onto the soft substrate, the liquid metal droplet (Gallium Indium eutectic, Ga: In, 75.5 : 24.5 wt%, Alfa Aesar) was dragged across the Cr/Au trace using a syringe, and a thin liquid metal trace would be accumulated on top of the Cr/Au circuit due to the intermetallic wetting effect (2). After connecting the liquid metal circuit to thin copper wires (diameter, 120 μm), uncured PDMS (thickness, ~ 200 μm) was cast onto the soft substrate and cured at 65 °C for 6 h to encapsulate the soft circuit and fix the copper wires for a robust connection.

Supporting Note 5

Bonding the electrodes. For fixing and connecting the SIC array to the circuit on a PCB, first, the two pins of an artificial cilium were inserted into the through holes of the PCB from the back (the side without copper). Then, we filled the voids between the electrodes and the holes with silicone adhesive (SIL-POXY™, Smooth-on, Inc.) for sealing. Furthermore, silver conductive paint (Electrolube, UK) was used to connect the electrodes and circuit with the help of acupuncture needles (SEIRIN J15 type, $\emptyset 0.1 \times 15\ \mathrm{mm}$, 3B Scientific®). Finally, clear protective lacquer (Electrolube, UK) was sprayed to cover and protect the circuit when the silver conductive paint completely dried.

Supporting Note 6

Data collection system. A USB multifunction I/O device (USB-6343, National Instruments™) was used for data collection. The sampling rate was set as 1 kHz for most of the time considering both the signal accuracy and data size, and 20 kHz was used when comparing the signals with the image analysis results from a high-speed camera.

Supporting Note 7

Rotating magnetic field generation system. A 2-layer Halbach array system was used for generating a uniform magnetic field, and the design and simulation results on the magnetic system are shown in **fig. S30**. In general, a direct current (DC) geared motor (DCX22S, Maxon, Switzerland) was used to control the rotation of the Halbach array, and the rotating speed can be controlled by the input voltage of the DC motor, which has been calibrated before the experiment.

Supporting Note 8

Characterization of the cilia sensor and actuator. As shown in **fig. S2**, the material of the ferromagnetic-elastic layer of the SIC had a density of $(1.83 \pm 0.052) \times 10^3 \mathrm{kg} \cdot \mathrm{m}^{-3}$ and an average Young's modulus of 530 kPa measured by a tensile testing machine (5940 series, Instron GmbH). The remnant magnetization was 32.8 emu/g measured by a vibrating-sample magnetometer (VSM, EZ7, Microsense). The ferromagnetic-elastic sheet body was superhydrophobic with a contact angle of ~ 128° that was measured by a contact angle goniometer (DSA100 series, KRÜSS GmbH). The sheet resistance of the integrated LIG sensor was 35.98 ± 0.91 Ω/square measured by four-point probe system equipped with soft contact probes (Ossila BV).

Supporting Note 9

Preparation of the boundary walls and fluid channels. The boundary walls used in the experiment were prepared by a fused deposition modeling 3D printer (Ultimaker S5, Cura) using polylactic acid filament. The boundary walls used in the experiment of distributed obstacle sensing were designed with a width of 25 mm, and the height difference between adjacent steps was 0.5 mm. The tubes used in flow speed sensing were prepared by a stereolithography 3D printer (Form 3B+, Formlabs) using transparent resin (Clear V4, Formlabs). The inner tube size was 2.3 mm × 1.5 mm (height by width), and the length is 15 mm. The tube was connected to a medical silicone hose with an inner diameter of 2 mm for the flow speed test (**fig. S27**).

Supporting Note 10

Sensor signal acquisition and data analysis. We measured and recorded the partial voltage of the SIC through a simple voltage divider circuit with a paired resistor, the 5-V power supply was provided by USB-6343 (National Instruments™), and the paired resistor was selected as 5 kΩ since the resistance of the integrated sensors is 3 kΩ ~ 6.5 kΩ. Then, the saved signal data were transformed from voltage to resistance by $R_s = (V_s \times R_{paired})/(V_0 - V_s)$, where R_s is the resistance of the LIG-based sensor, V_s is the measured raw sensor voltage, R_{paired} is the resistance of the paired resistor, and V_0 is the voltage of the power supply. **Figure S10** shows the schematic view of the LDA model and the workflow. The continuously collected signal (~ 2 min) was segmented into several segment signals as the dataset for the relevant setting environment (the peak positions are used as marks to split the signal, and each piece of the data contains the signal of resistance change for around 12 complete actuating cycles (changed according to the actuation frequency). As the maximum and minimum value of the signal also contains important information from the detected environment, we normalized the signal data by setting the median value of each segment to zero. With labels attached to each signal, we extracted the features and processed the signal classification and signal prediction with the LDA method and curve fitting. For evaluating the quality of the LDA classification model, we built the training set and testing set (80% data for training and 20% for testing) based on the calibration feature dataset and then save the trained LDA model. Then, we extracted the prototypical features of each category and calculated the distance between the feature of the query set and prototypical features to predict the classification of new data. All the signal processing processes were done by customized code in python 3.8 with Scikit-learn and NumPy (3, 4).

Supporting Note 11

Image acquisition and analysis. The images of SICs were taken by an industrial complementary metal oxide semiconductor (CMOS) camera (Blackfly S-U3-32S4C-C, Teledyne Flir™) and a high-speed digital camera (VisionResearch, Phantom v641). The high-speed camera was used for recording and analyzing the movement of second-order oscillation of the SIC when actuated in the ambient environment, and the frame rate was set as 7,700 frame per second (fps). The other image sequences are taken by the industrial COMS camera at a frame rate of 200 fps. The image processing part on the captured cilia image sequences utilized Python 3.8 and the OpenCV library (5). After a series of processing such as image binarization, contour recognition and curve fitting, the information on cilia motions (e.g., the position, bending angle and speed at each moment) was obtained and used for subsequent data analysis. ImageJ was also used during the image analysis process (6).

Supporting Note 12

Equivalent stiffness and performance of the test sample in stiffness characterization. A cantilever beam model was used for calculating the stiffness of SIC and the scaled-up sample, and the parameters are shown in **fig. S26**. As the stiffness $K = F/\delta = 3EI/L^3$, where F is the total load, δ is the bending deflection, E is the Young's modulus, I is the bending moment of inertia of the beam, and L is the length of the beam. And $I = \frac{1}{12}bt^3$, where b is the width and t is the thickness of the beam. So $K \sim Ebt^3/L^3$, and the test beam shows equivalent stiffness as the SIC beam when K is the same. As the size of the proposed SIC is b_{SIC} = 0.8 mm, t_{SIC} = 0.12 mm, and L_{SIC} = 1.8 mm, the relevant sample in the stiffness test is prepared with b_{test} = 10.8 mm, t_{test} = 0.56 mm, and L_{test} = 20 mm for same stiffness. For the coefficient of the applied magnetic and elastic forces acting on the SIC, they can be characterized by a magnetic number $Mn = BL(\frac{bt}{\mu_0 EI})^{0.5}$ since the test sample is magnetized along the long beam. Here, $B = |\mathbf{B}|$ is the magnitude of the external-applied magnetic flux density, and μ_0 is the permittivity of free space. As $Mn \sim BL/t$, and L_{test}/t_{test} is about two time of L_{SIC}/t_{SIC}, the performance of tested beam is comparable to that of the SIC under half of the applied magnetic fields. It is worth noting that the magnetoelastic number with similar definition has been used in previous numerical simulations to describe kinematics of magnetic cilia (7).

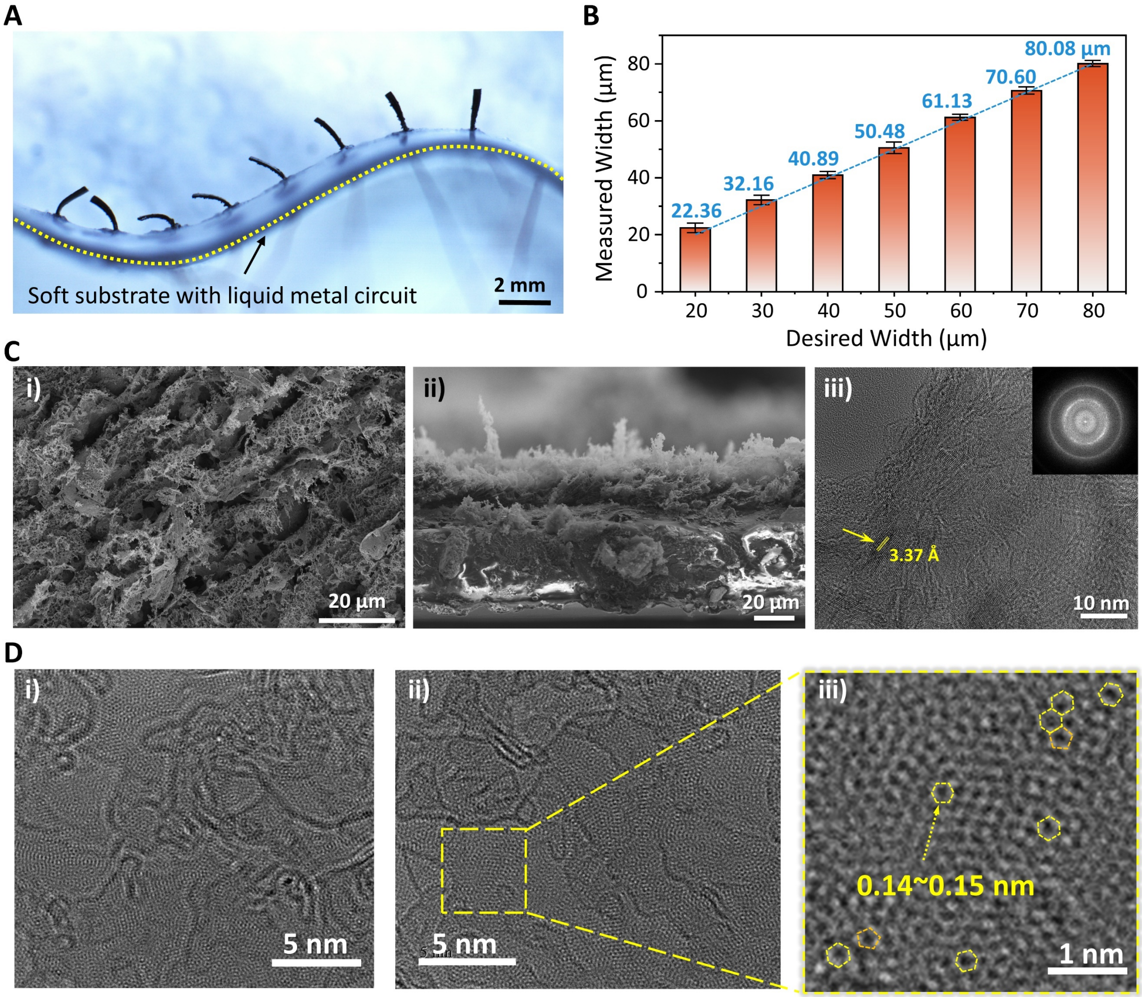

Fig. S1. Characterization of the dimensions and other material properties of the SIC. (A) SIC array with a soft substrate and embedded liquid metal-based circuit, which allows the artificial cilia array to be conveniently deployed on complex shaped surfaces. Scale bar, 2 mm. **(B)** The fabrication accuracy of the LIG line width with laser engraving method. **(C)** The SEM and HRTEM images of LIG before transferred to the magnetic elastomer layer. (i) and (ii) show the grass-like LIG holds numerous micro/nano structures with fluffy forms due to the laser engraving process with a thickness of ~20 μm. iii) shows few-layer features and highly wrinkled structures (marked by arrow) and average lattice space of $\sim\mathbf{3.4}$ Å. Insert image shows FFT of the LIG sample image. Scale bar, 20 μm, 20 μm, 10 nm. **(D)** The high-resolution TEM (HRTEM) image of LIG taken at the edge of a LIG flake. (i) shows the ultra-polycrystalline property with grain boundaries and (ii) and (iii) shows the structure of hexagons and pentagons; scale bar, 5 nm, 5 nm, 1 nm.

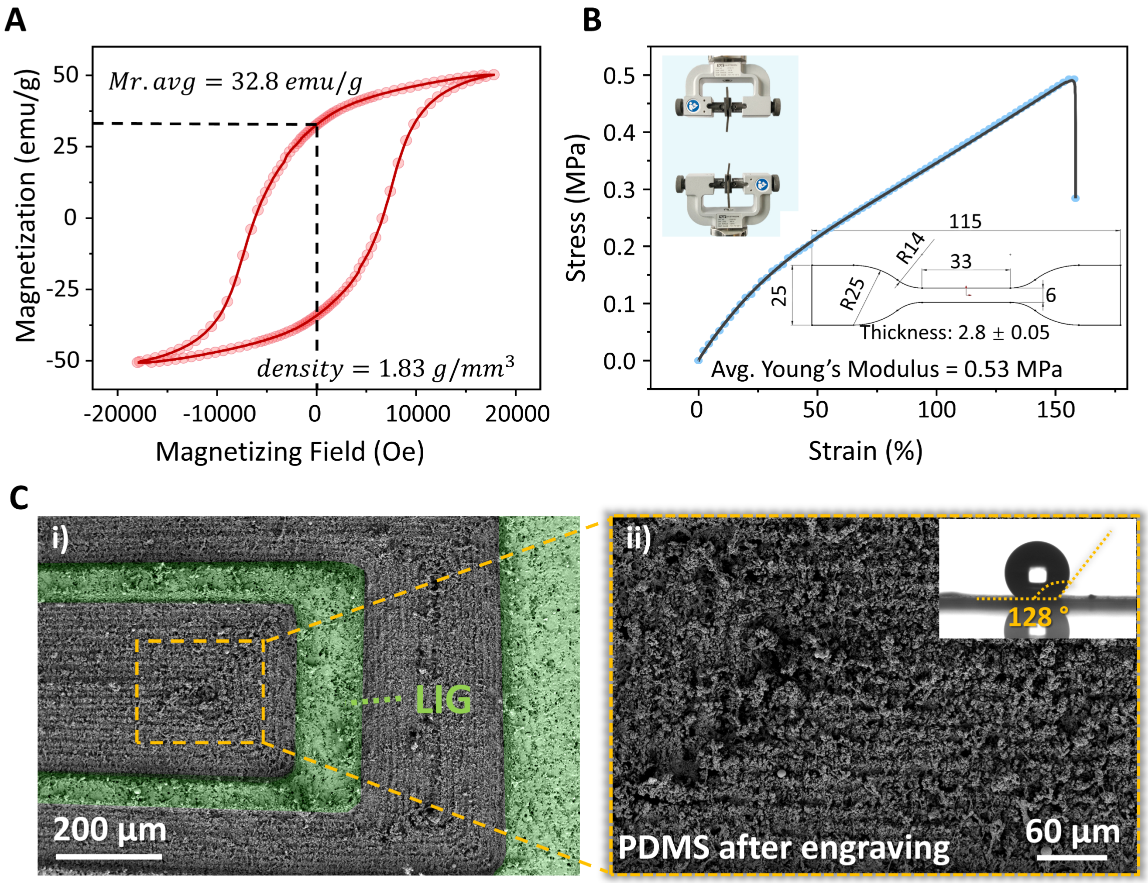

Fig. S2. Material characterization of the fabricated ferromagnetic elastomeric sheet and LIG. (A) The magnetic hysteresis loop of the magnetic elastomer matrix sample (weight ratio of PDMS to NdFeB, $1:1$; average density, $1.83\ \mathrm{g/mm^3}$), and the average remanent magnetization ($\boldsymbol{M_r}$) is $32.8\ \mathrm{emu/g}$. **(B)** The strain-stress test on the magnetic elastomer matrix sample (ASTM-D412-C type), and the average Young's modulus is $0.53\ \mathrm{MPa}$. **(C)** The SEM image of the SIC after laser engraving. The green part in (i) is LIG and gray part is the PDMS after removing LIG layer by laser engraving. (ii) shows the details of the PDMS after engraving, which is superhydrophobic (contact angle is $128°$) due to the micro structure on the surface; scale bar, $200\ \mu\mathrm{m}, 60\ \mu\mathrm{m}$.

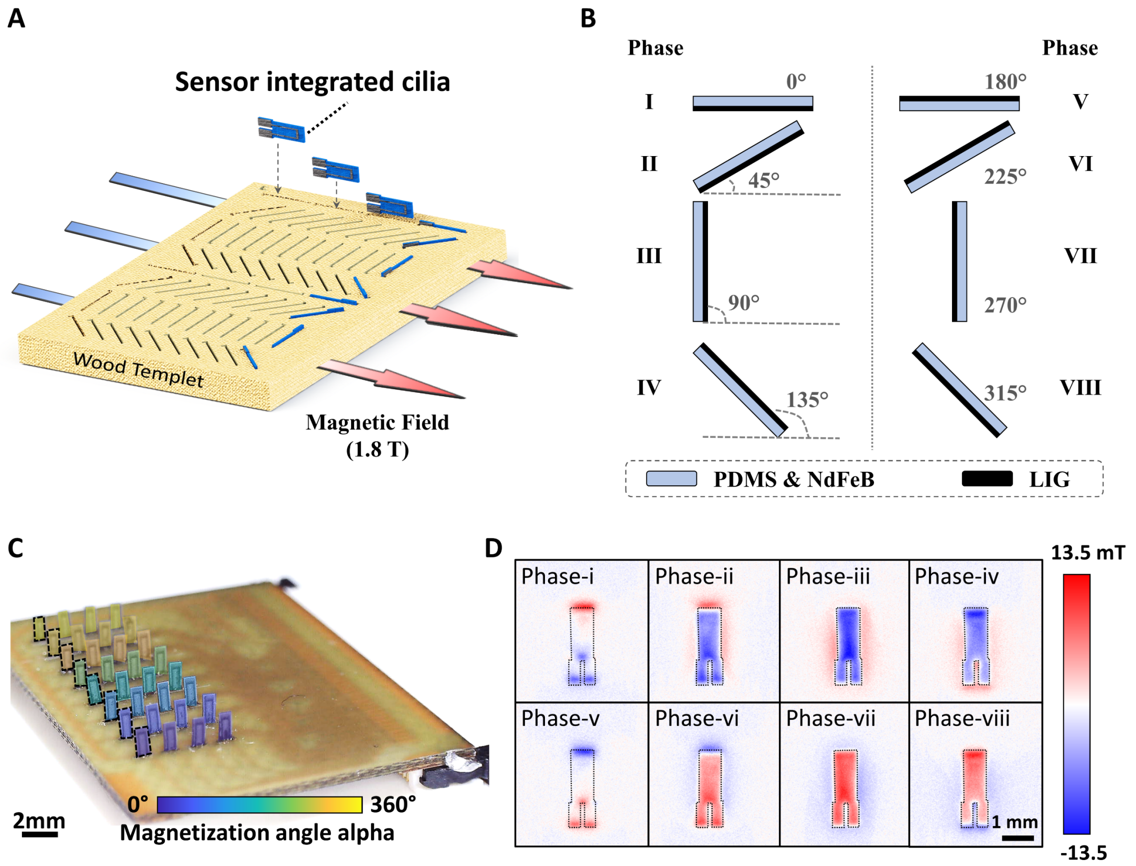

Fig. S3. Design and jig-assisted magnetization of the artificial cilia with a programmable phase. (A) The schematic of the jig-assisted magnetization process. A wood jig (medium-density fiberboard, MDF; thickness, 2 mm) designed with holes at different phases were prepared by laser cutting (LPKF U3), and the cilia were placed into the holes with tweezers for magnetization. The cilia were magnetized by a uniform magnetic field (B_m = 1.8 T) in a direction parallel to the short side of the jig. **(B)** The schematic of cilia with different phases, taking the design with $45°$ phase difference as an example. **(C)** Image of a 4×8 SIC array with different magnetization profiles $\boldsymbol{M}(s)$ that integrated on a PCB board for signal acquisition. The SIC array can response to the external magnetic field $\boldsymbol{B}$ in designed sequency with metachronal coordination. Scale bar, 2 mm. **(D)** Magnetic flux density measurements of the magnetically programmed cilia samples with phase difference of $\Delta\phi = 45°$ between adjacent cilia. Scale bar, 1 mm.

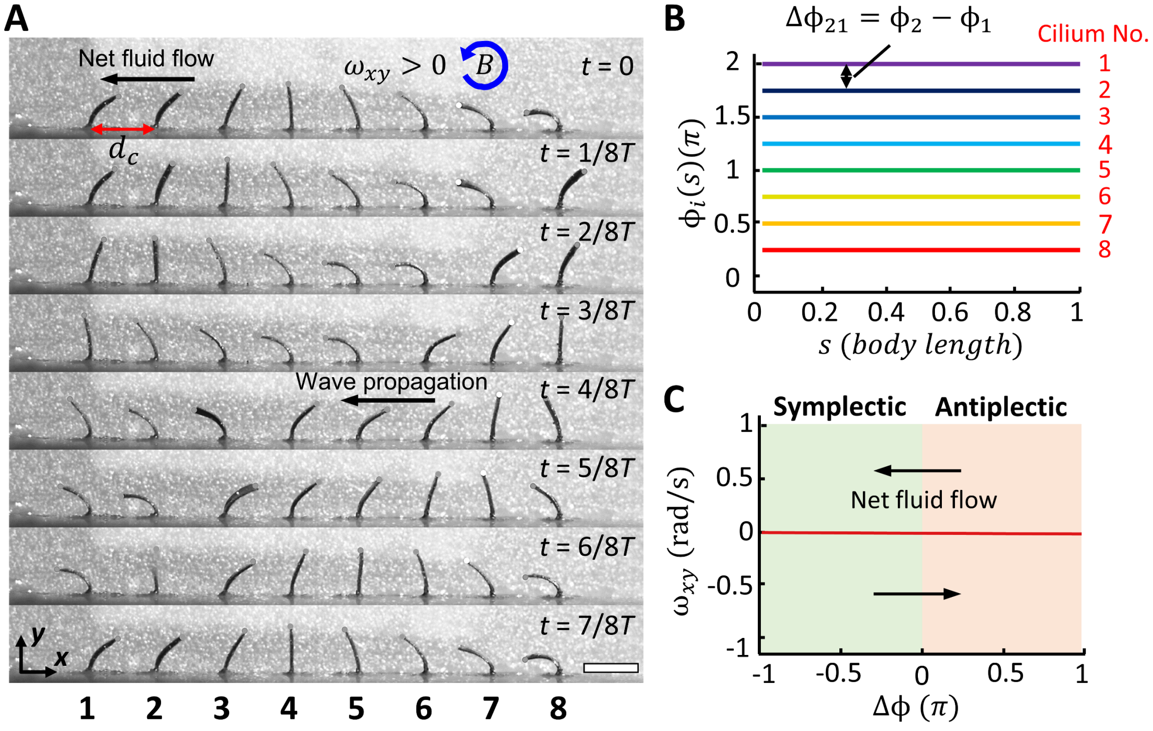

Fig. S4. SIC array with programmable nonreciprocal motions and metachronal waves. (A) Video snapshots of a SIC array with metachronal waves pumping viscous fluids (glycerol). $\boldsymbol{B}(t)$: f = 2 Hz and B_m = 35 mT. Scale bar, 2 mm. **(B)** The magnetization phase profiles $\phi(s)$ for the SIC in (A). The neighboring cilia have the same constant magnitude in their $\boldsymbol{M}(s)$. **(C)** The linear mapping from $\Delta\phi_x$ to ω_{xy} in the metachronal waves.

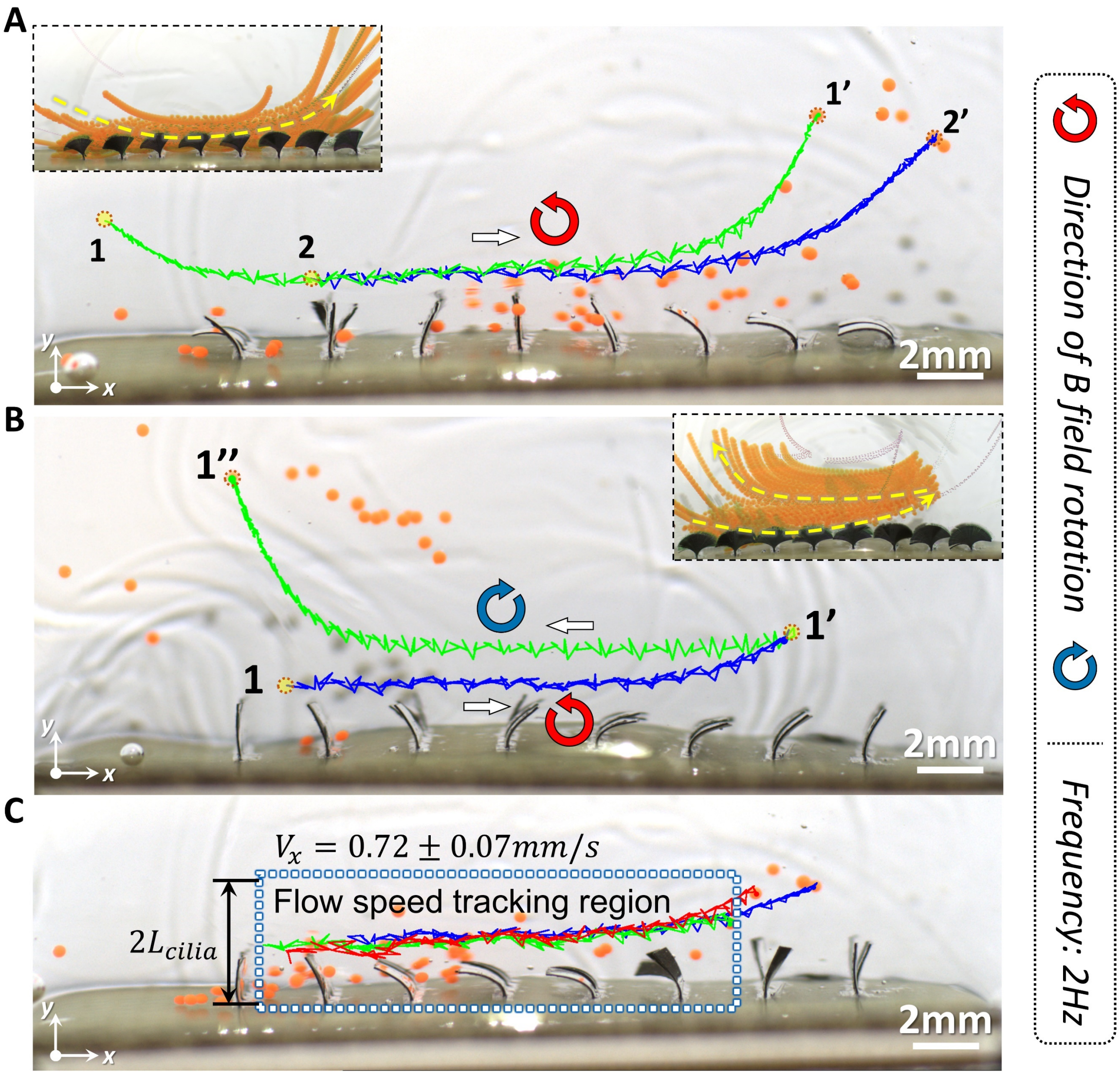

Fig. S5. Flow control and particle transport at low Reynolds number using magnetically controlled cilia array with metachronal coordination. Snapshots and overlapped trajectories of transporting neutrally buoyant particles by a 4×8 cilia array at glycerol environment. Fluorescent orange polyethylene microspheres were used as cargo and tracer of the flow direction. The flow direction can be tuned by the rotation orientation and frequency of the magnetic field. **(A)** One direction transportation. Insert shows the stacked image represented the immigration trend of all the particles. **(B)** Bi-direction particle transportation by changing the rotation orientation of the magnetic field. **(C)** By tracking the tracer particles, the flow generated by the SIC array holds a velocity of 0.72±0.07 mm/s. $\boldsymbol{B(t)}$: $\boldsymbol{f}$ = 2 Hz and $\boldsymbol{B_m}$ = 30 mT. Liquid environment, Glycerol. Scale bar, 2 mm.

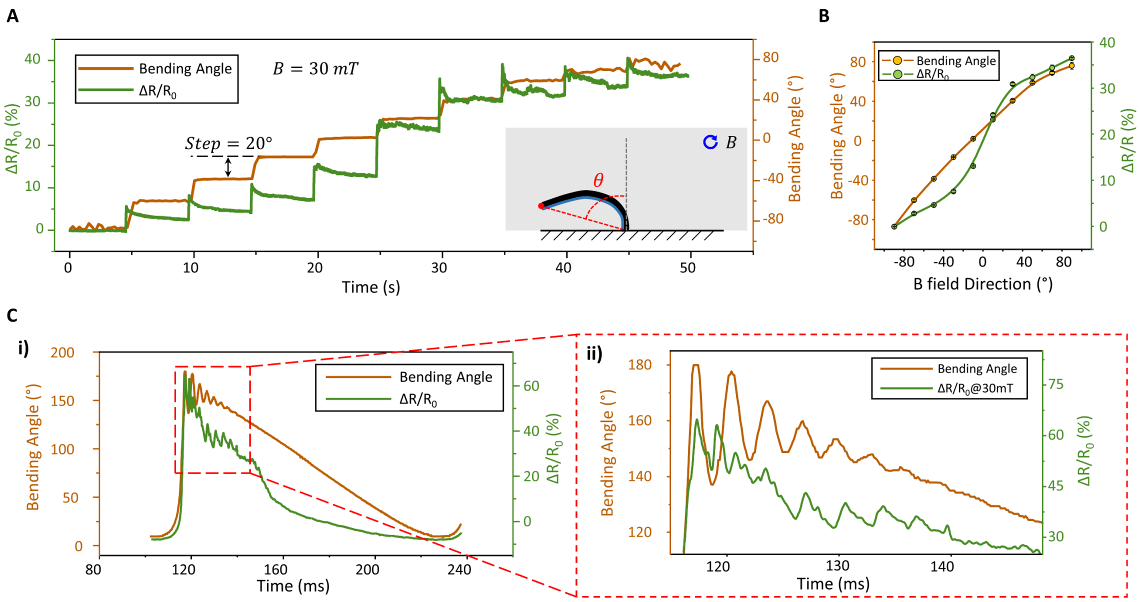

Fig. S6. Performance of the LIG-based sensor. (A) The comparison between the relative resistance changes and the bending angle change of the sensor-integrated cilium that responded to an external magnetic field. The bending angle is measured from a high-speed camera image sequence and the cilium is tested in air with B_m = 30 mT. **(B)** The bending angle and resistance change of the SIC as a function of the applied magnetic field direction. **(C)** Changes on the sensor signal and bending angle show a single cilium exhibit natural vibration during the change from power stroke to recover stroke and (ii) shows the recorded signals agreed well with the bending angles.

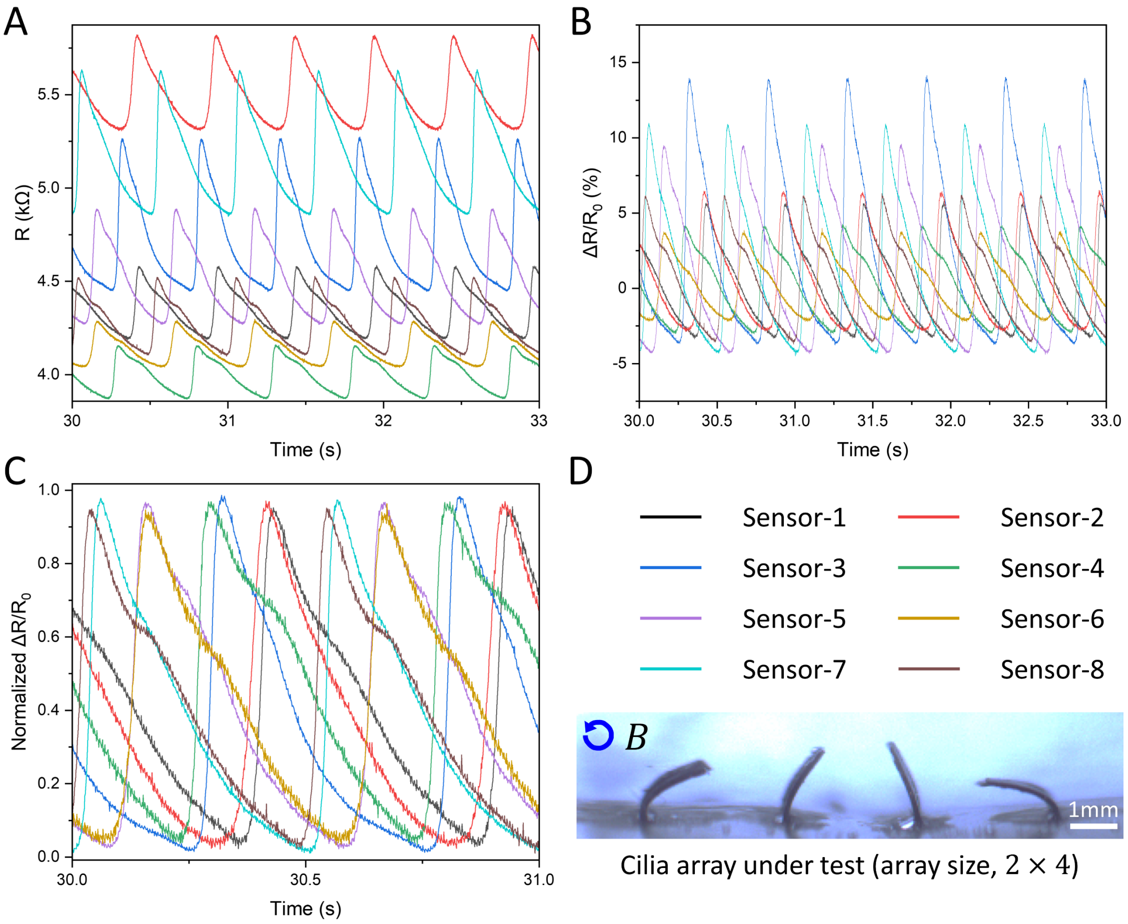

Figure S7. Raw signals and the related normalization process of a 2×4 SIC array (A) The recorded raw signals R during the magnetic actuation without noise reduction. The initial resistance of SICs ranges from 3.7 kΩ to 5.4 kΩ. Sampling frequency, 1000 sample/s. **(B)** The relative resistance changes $\Delta R/R_0$ of the SICs. **(C)** The normalized relative resistance changes of the SICs, normalization by scaling between 0 and 1. **(D)** The tested SIC array during actuation in a liquid environment. $\boldsymbol{B}(t)$:f = 2 Hz, B_m = 30 mT. Liquid viscosity, 195 cP. Scale bar, 1 mm.

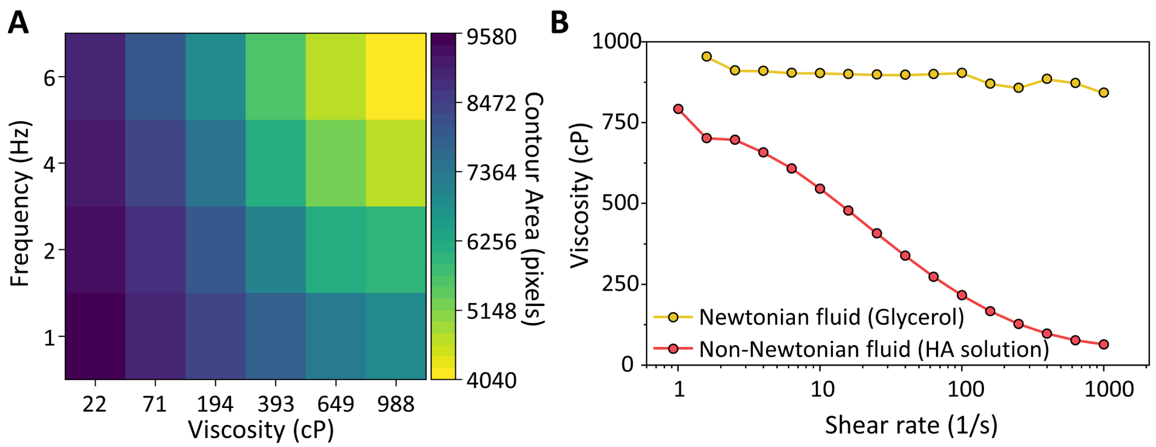

Fig. S8. Viscosity sensing ability of the SIC. (A) The plots of contour area as the sensor-integrated cilium actuated under different frequencies in a liquid environment with various viscosities. **(B)** The viscosity characterization of the non-/Newtonian fluids at different shear rate. The two fluids have similar viscosities at a low shear rate, while the non-Newtonian fluid shows a significant shear-thinning property.

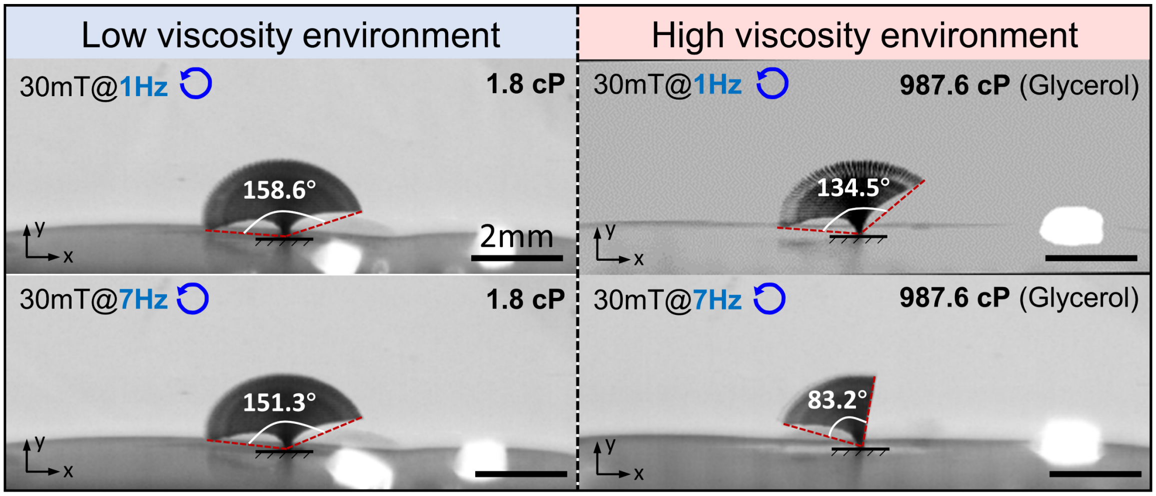

Fig. S9. The comparison of swing angle of SIC under different actuation frequencies and liquid environment with different viscosities. While the magnetic field strength is set as 30 mT, the swing angle of SIC decreased less than 5% under low viscosity liquid environment (1.8 cP) when actuation frequency increased from 1 Hz to 7 Hz, and the swing angle decreased around 38% under high viscosity liquid environment (987.6 cP). Scale bar, 2 mm.

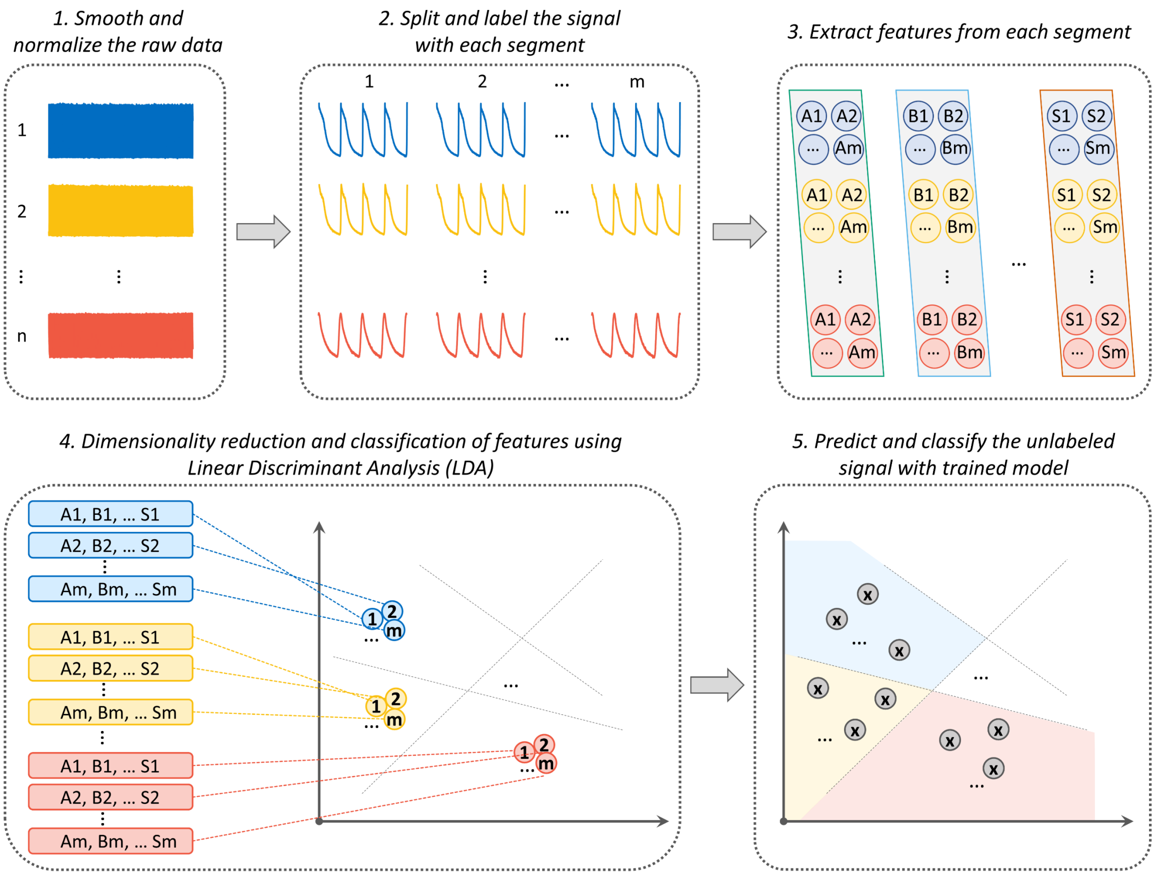

Fig. S10. The schematic view of the machine learning model using LDA for the sensing signal analysis. Firstly, the data of resistance change that containing the motion and posture information of the sensor integrated cilia was recorded and saved after smooth and normalize. Secondly, the signals were split into small segments with labels. Thirdly, the features were extracted from each segment and formed a multidimensional vector to describe the relevant signal segment. Then, dimensionality reduction and classification of the features were performed using LDA. Next, the trained LDA model was saved for unknown signal classification and prediction.

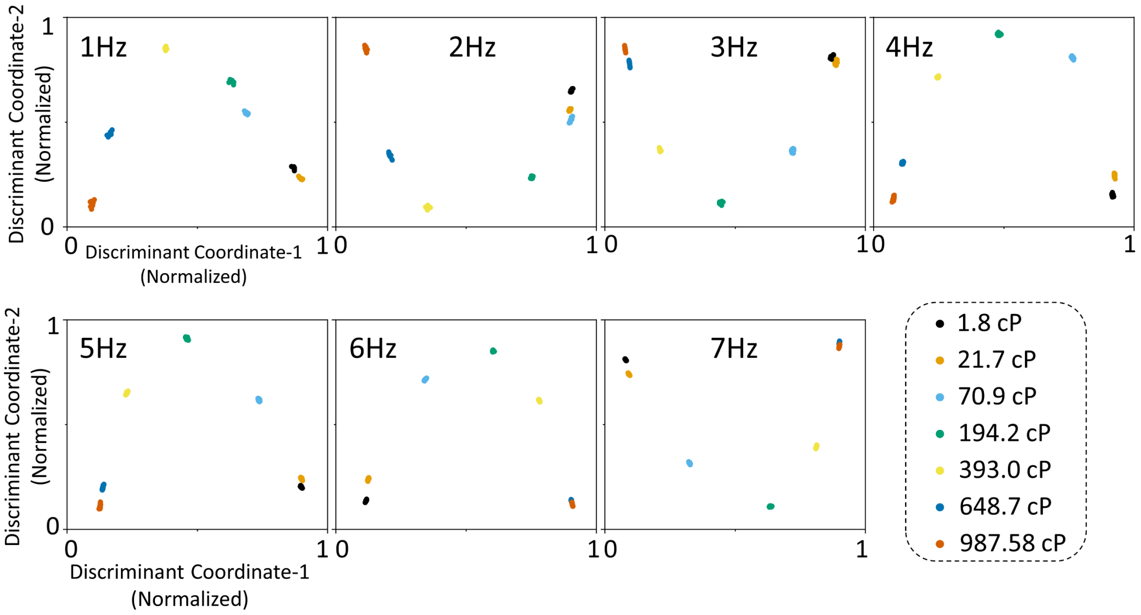

Fig. S11. Classification and visualization of the signals under different actuation frequencies when sensing different fluid viscosity using LDA method. The sensor integrated cilia were tested under different viscosity environment (the mixture of glycerol and water at different weight ratio), and the signals of each setting were split into several segments, with 10 - 20 full drive cycle signals in each segment. After extracting features in both time and frequency domains, the signals were dimensionally reduced and classified using linear discriminant analysis (LDA). Clusters of points with the same color were signals in the same viscosity environment. $\boldsymbol{f}$ = 1 to 7 Hz, and $\boldsymbol{B_m}$ = 30 mT.

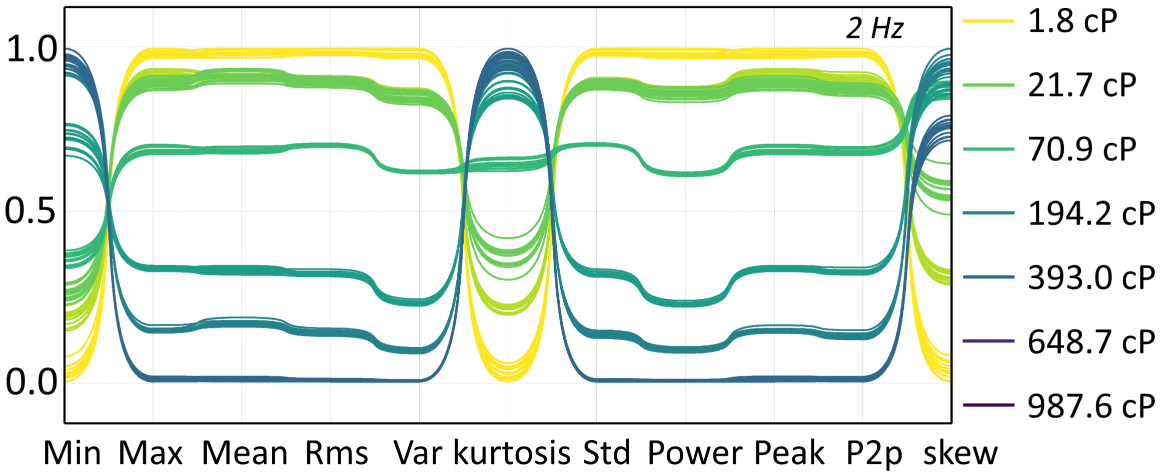

Fig. S12. Classification quality and contribution of each feature extracted from raw signals in viscosity sensing. The selected feature plotted with parallel coordinates, reveals the correlation of each eigenvalue with the viscosity change. f = 2 Hz, and B_m = 30 mT

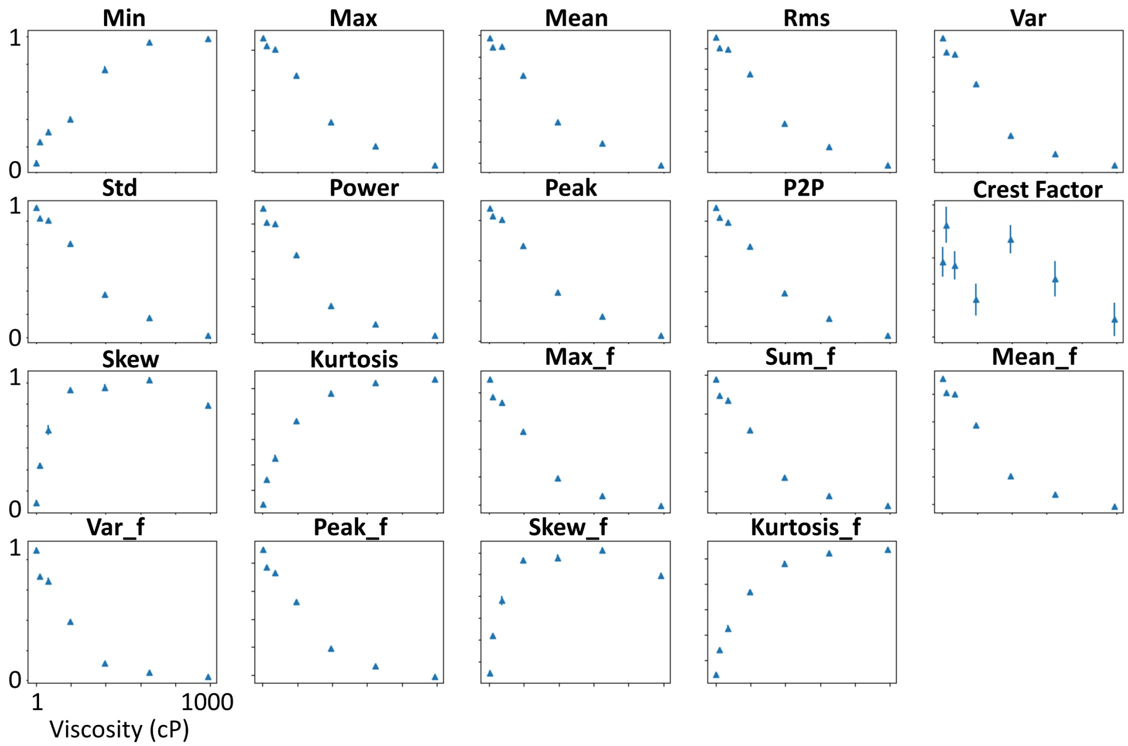

Fig. S13. Trends of all extracted signal features when sensing different environmental fluid viscosities. 12 time-domain features and 7 frequency-domain features are used in revealing the signal change in different viscosity environment. The features shown a high correlation with changes in the viscosity were chosen for curve fitting and the prediction of the viscosity in unknown environment. $\boldsymbol{f}$ = 2 Hz and $\boldsymbol{B_m}$ = 30 mT.

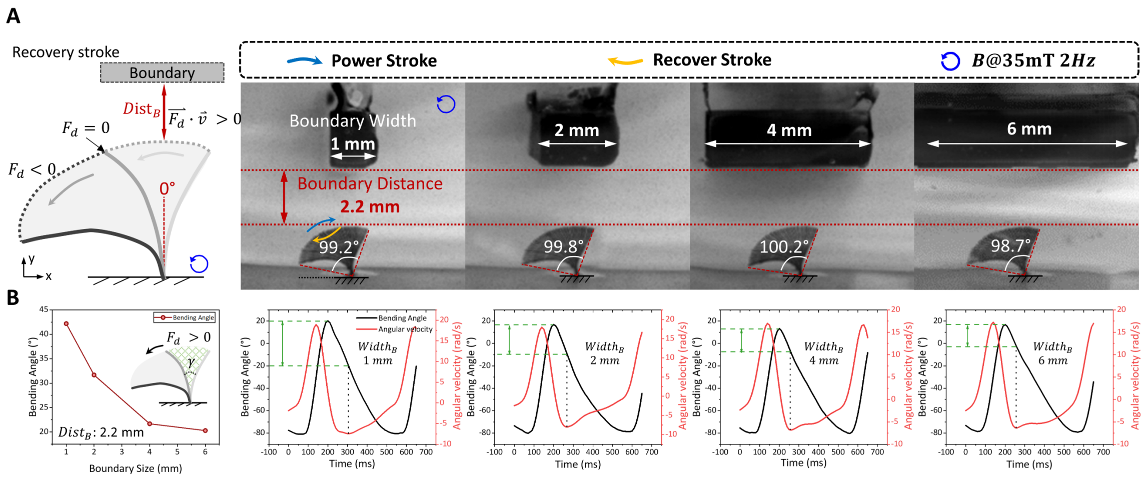

Fig. S14. Imaging-based analysis of SIC motions under different boundary conditions. (A) The stage of acceleration movement in the power stroke ($\overrightarrow{F_d} \cdot \vec{v} > 0$) is chosen in analyzing the influence of boundary condition to the ciliary motion. **(B)** The swing angle γ during acceleration movement in the power stroke is measured and analyzed, in which the boundary distance is fixed to 2.2 mm and the boundary size ranges from 1 mm to 6 mm.

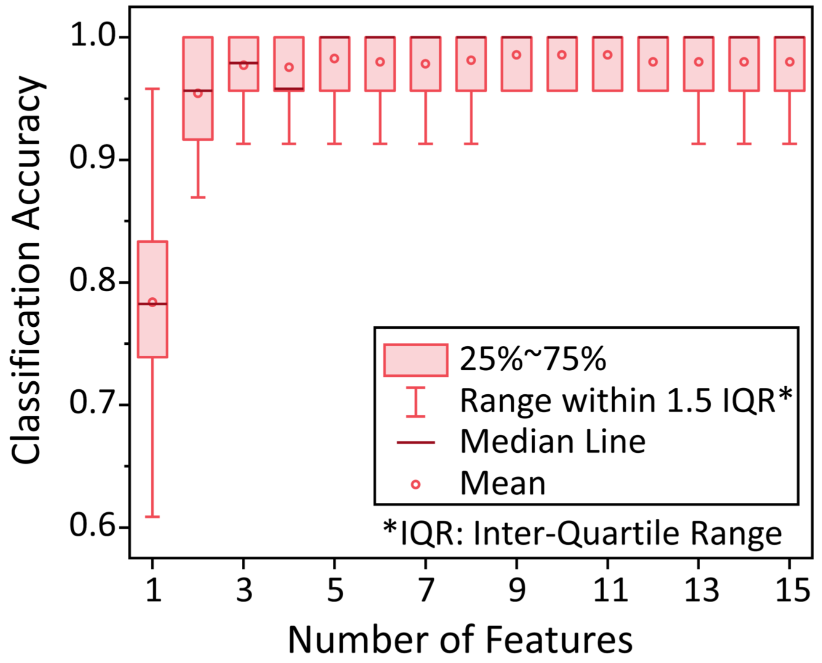

Fig. S15. Classification accuracy of the LDA model with different number of features in sensing the solid boundary distance. The classification accuracy is tested by changing the number of features that used in training the LDA model for boundary distance prediction. Repeated stratified K-fold cross validator (number of folds, 10; number of time cross-validator needs to be repeated, 3) is utilized to evaluate the LDA model. A dataset of 234 labeled samples with 15 classes are used in this test. The median of classification accuracy is 0.783,0.957,0.979,0.958,1.0 when the number of utilized features change is increased from 1 to 5. Boundary length, 10 mm; boundary distance, from 200 μm to 5,000 μm; liquid environment, glycerol.

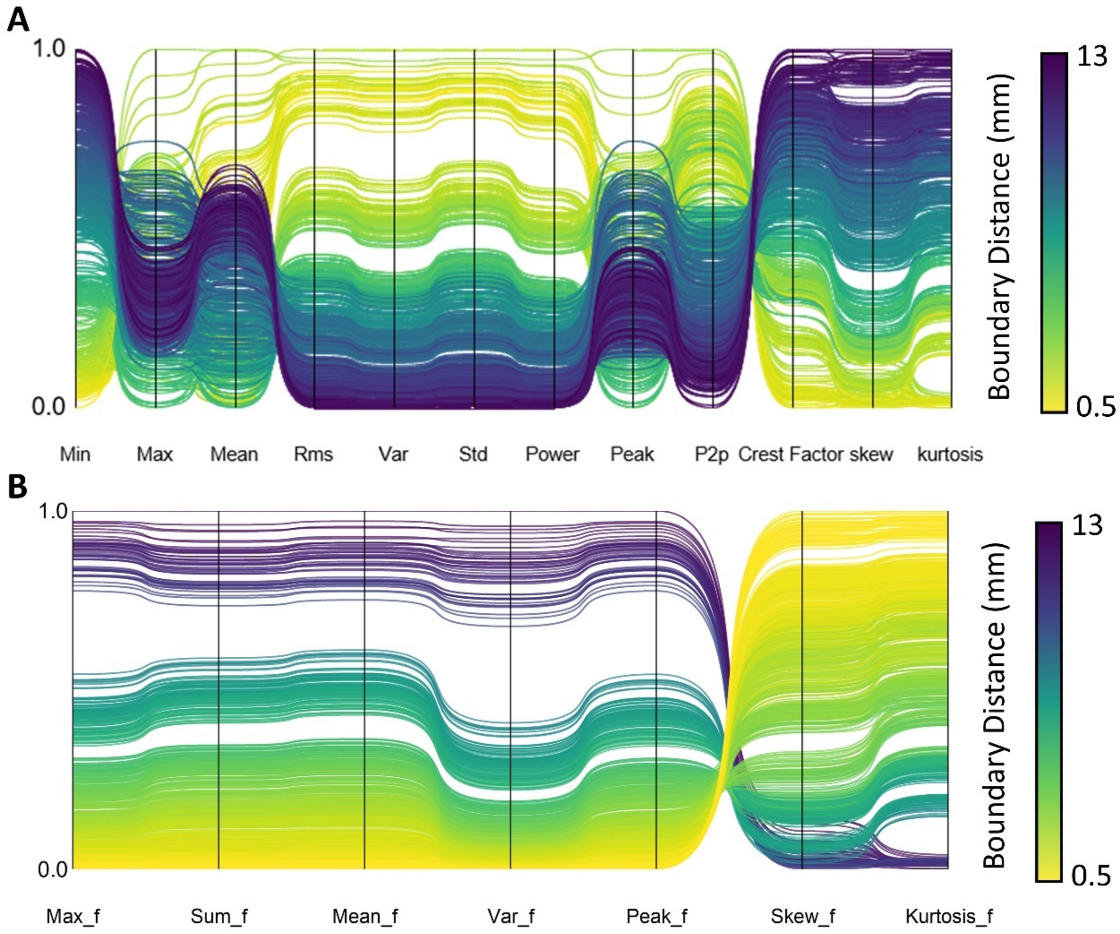

Fig. S16. Classification quality and contribution of each feature extracted from raw signals in boundary distance sensing. A parallel coordinates plot was used to visualize and compare the features that used in boundary distance sensing. the values in each feature were normalized to 0-1. The intra-class aggregation and inter-class discrimination of each feature was well visualized. **(A)** shows the changes in time-domain features and **(B)** shows the changes in frequency-domain features. Boundary length, 10 mm; boundary distance, from 0.5 mm to 13 mm; liquid environment, glycerol.

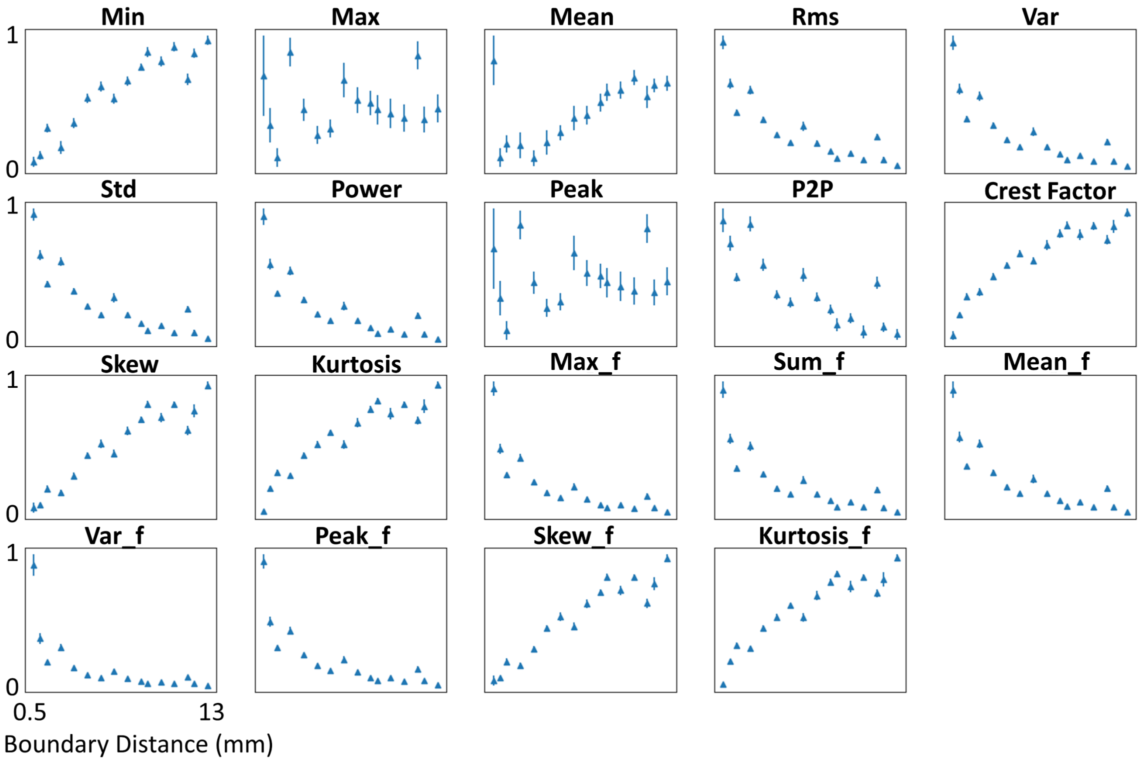

Fig. S17. Trends of all extracted signal features when sensing the solid boundary distance. The trending is visualized by using different signal features operator as a function of the boundary distances. 12 time-domain features and 7 frequency-domain features are used in revealing the signal change in different boundary distance situation. The features that show a high correlation with changes in the boundary distance will be chosen for curve fitting and the prediction of unknown distance object. $\boldsymbol{B}(\boldsymbol{t})$: $\boldsymbol{f}$ =2 Hz and $\boldsymbol{B_m}$ =30 mT.

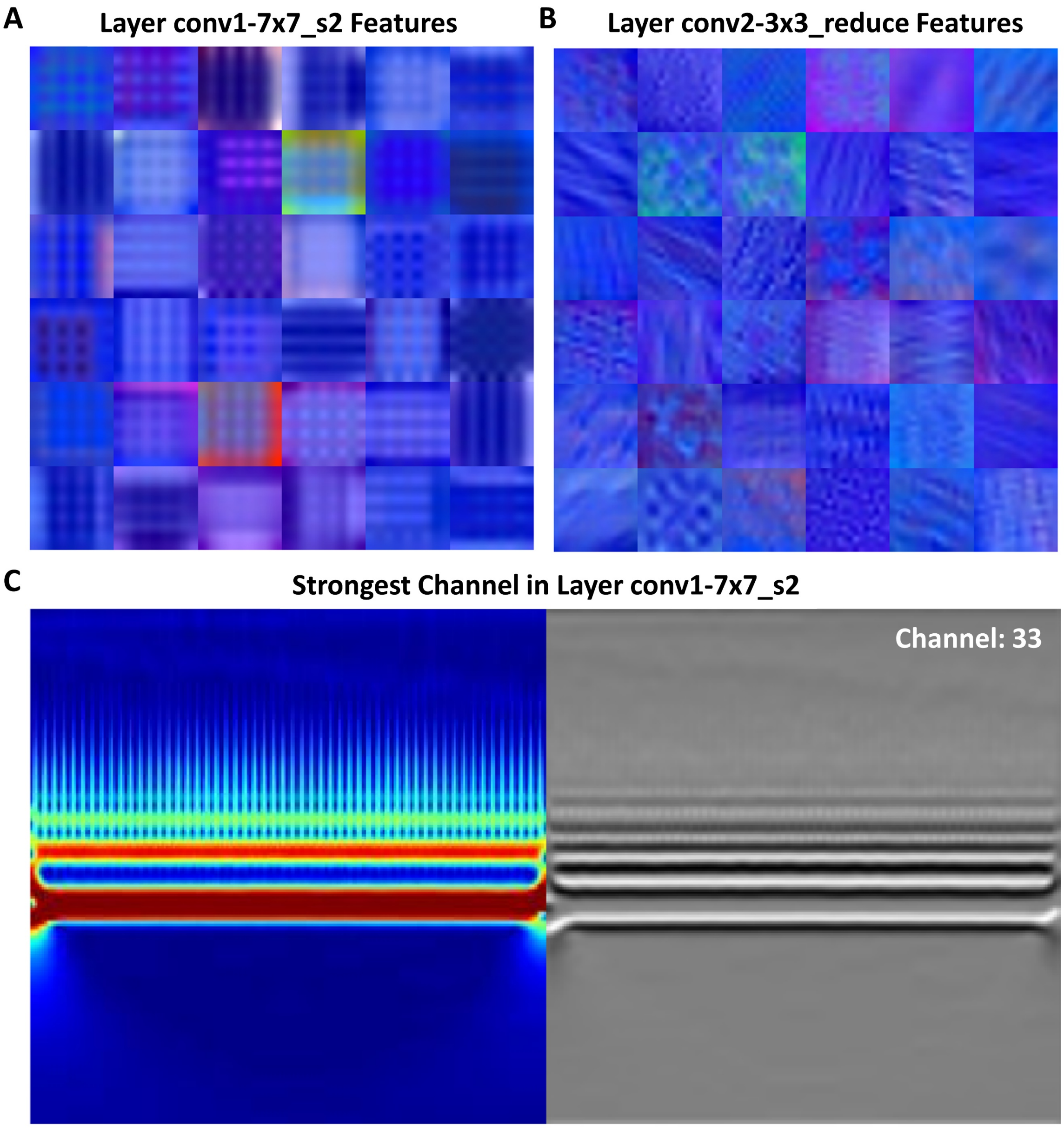

Fig. S18. Visualization of the trained conventional neural network. As there are multiple convolutional layers in the GooLeNet we employed, the convolutional layers towards the beginning mainly learn small and low-level features, while the layers towards the end learn larger features. **(A)** Visualization of the first convolutional layer by network filter weights, and the learned first 36 features are selected as shown, which mostly contain edges and colors. **(B)** The selected first 36 features that learned in second convolutional layer mainly detect more complex patterns. **(C)** The original image and the strongest active channel in the first conventional layer on the selected image.

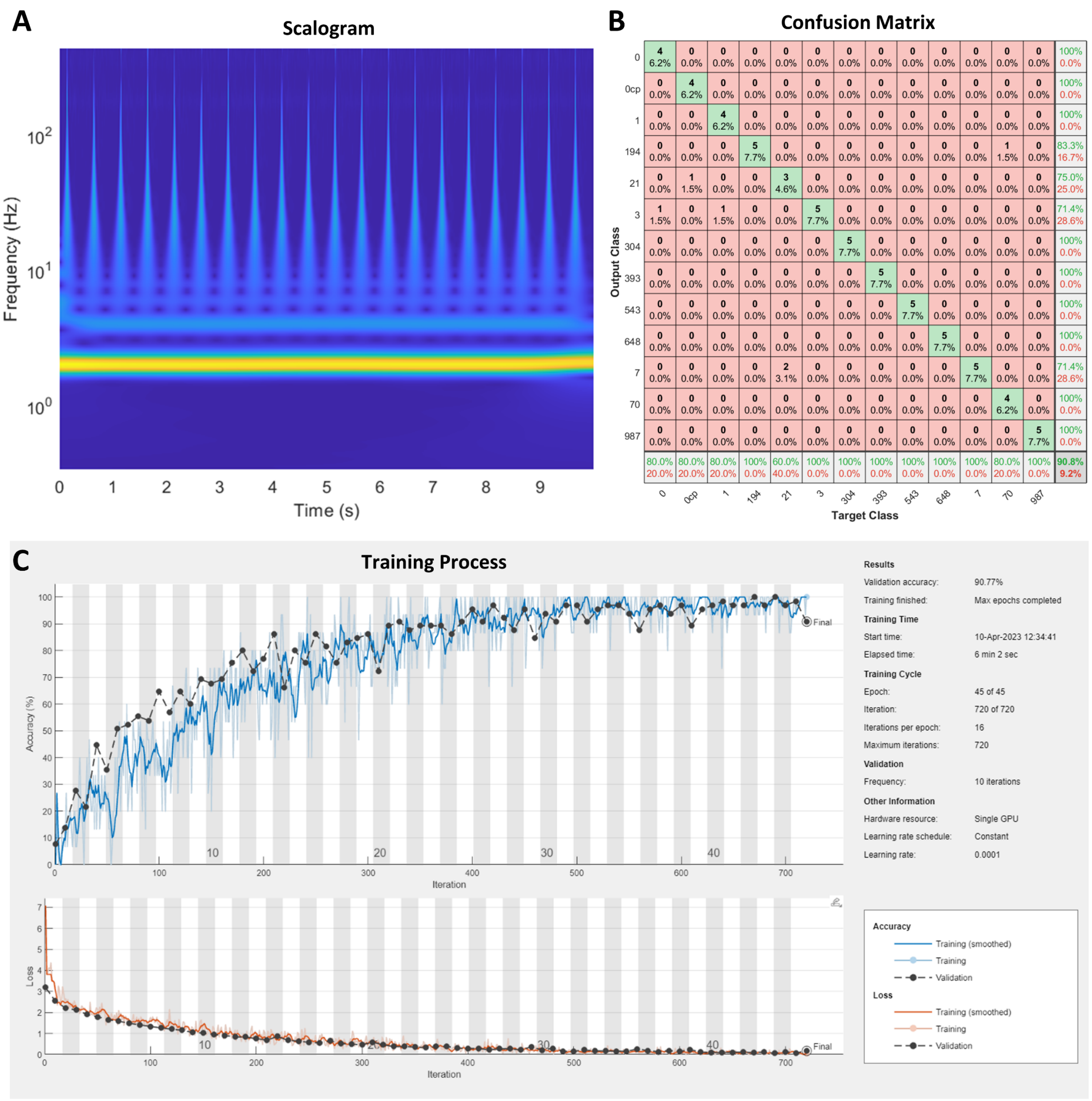

Fig. S19. Recording of the model training in viscosity sensing. (A) The sample spectrogram in the training dataset. **(B)** The confusion matrix of test dataset with 13 categories of different viscosities. **(C)** Plot of the training process with an iteration of 720 and validation accuracy of 90.77%.

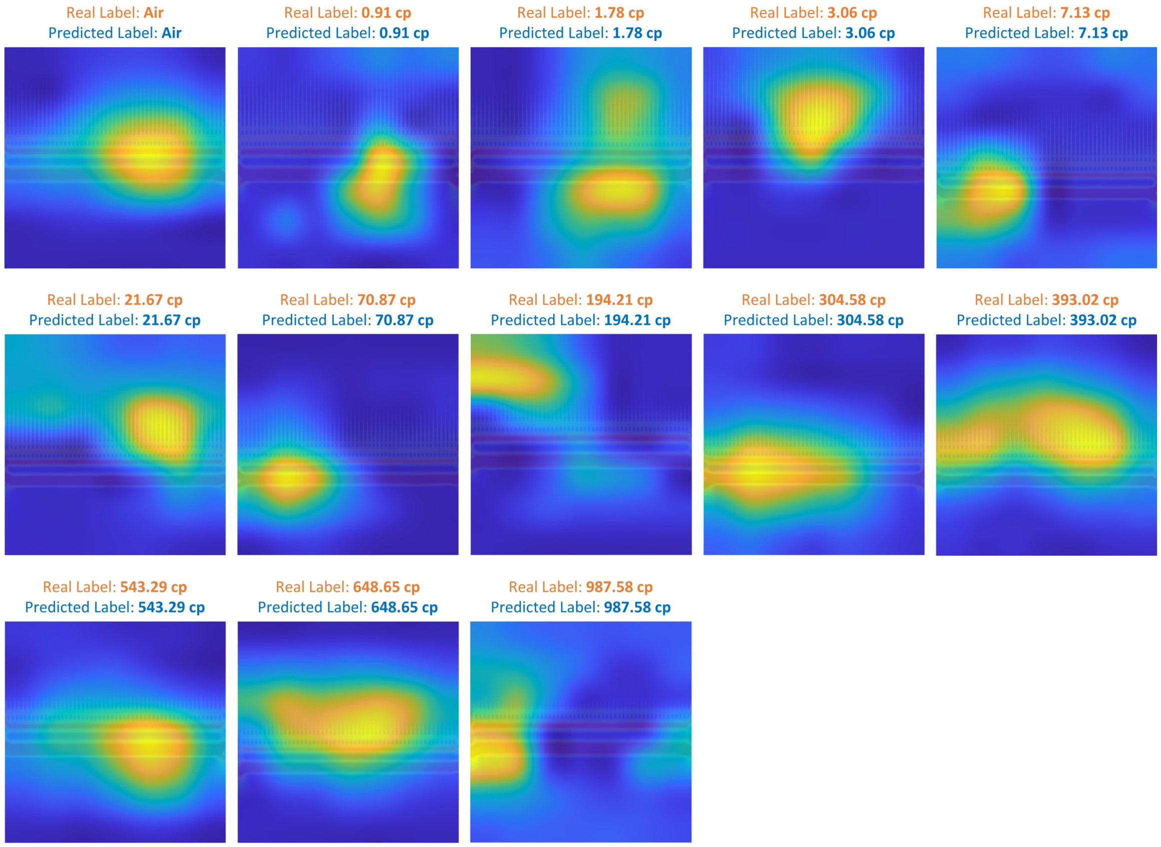

Fig. S20. Visualization of the trained network for viscosity sensing with gradient-weighted class activation mapping (Grad-CAM). Grad-CAM uses the gradient of the classification score with respect to the convolutional features to understand which part of an observation are most important for classification. For viscosity sensing, it shows that the important regions in the spectrograms mainly distributed around or above the frequency of magnetic actuation f = 2 Hz.

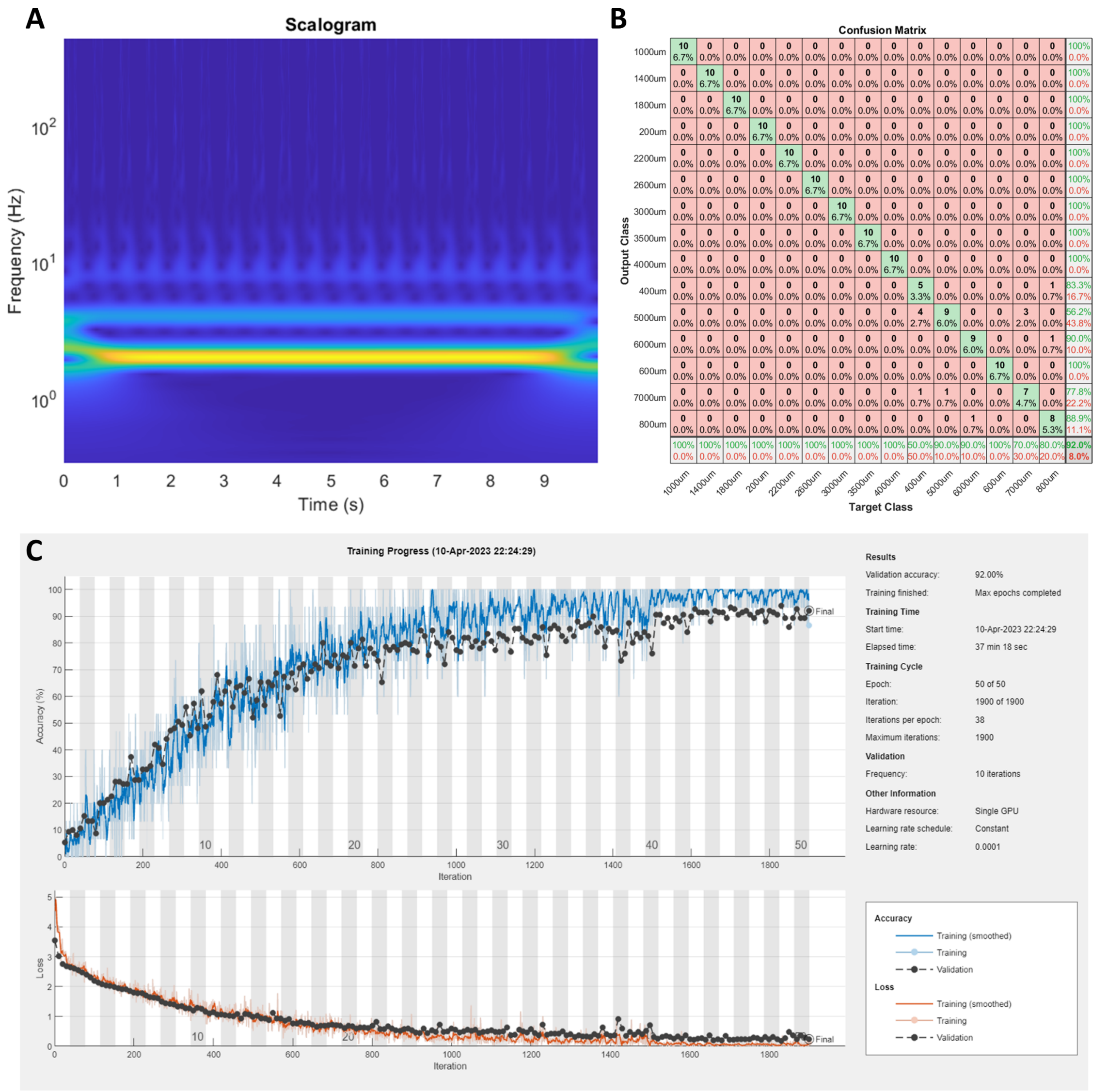

Fig. S21. Recording of the model training in boundary sensing. (A) The sample spectrogram in the training dataset. **(B)** The confusion matrix of test dataset with 15 categories of different boundary distance. **(C)** Plot of the training process with an iteration of 1,900 and validation accuracy of 92.0%.

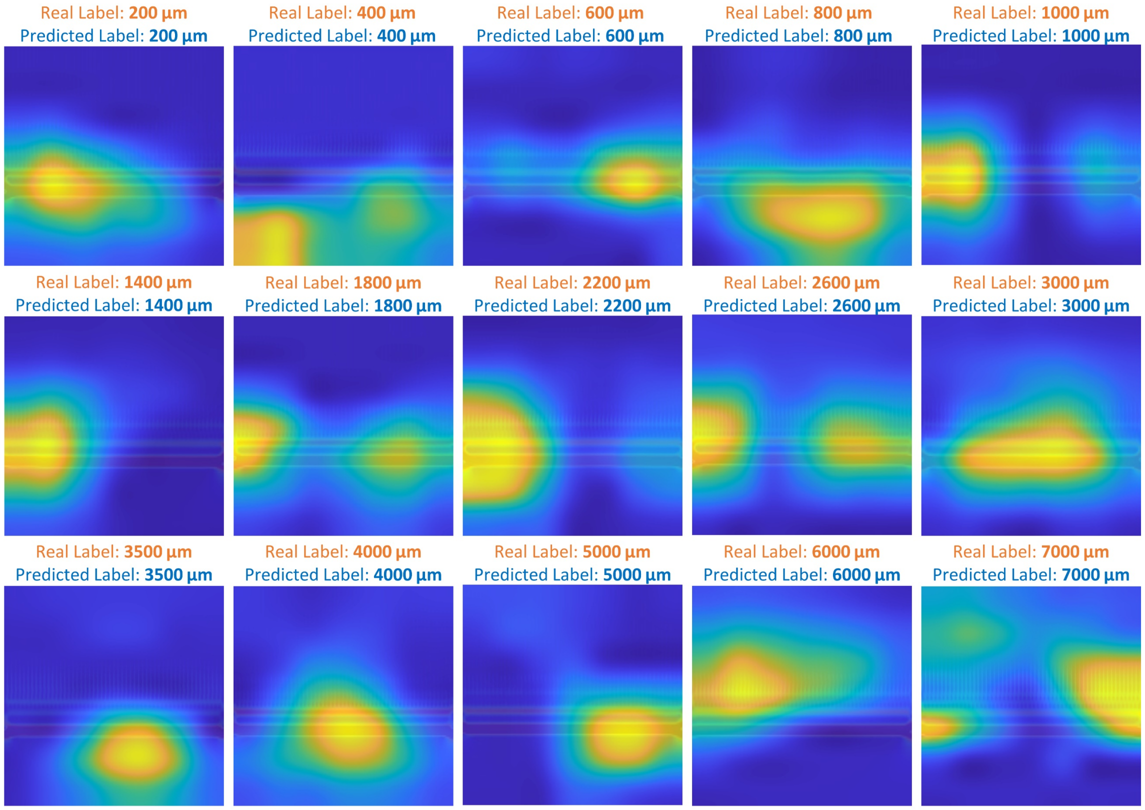

Fig. S22. Visualization of the trained network for boundary sensing with Grad-CAM. Grad-CAM results show that, for boundary sensing, the important regions in the spectrograms also mainly distributed around or above the frequency of magnetic actuation f = 2 Hz, and most of them require only one core interesting area.

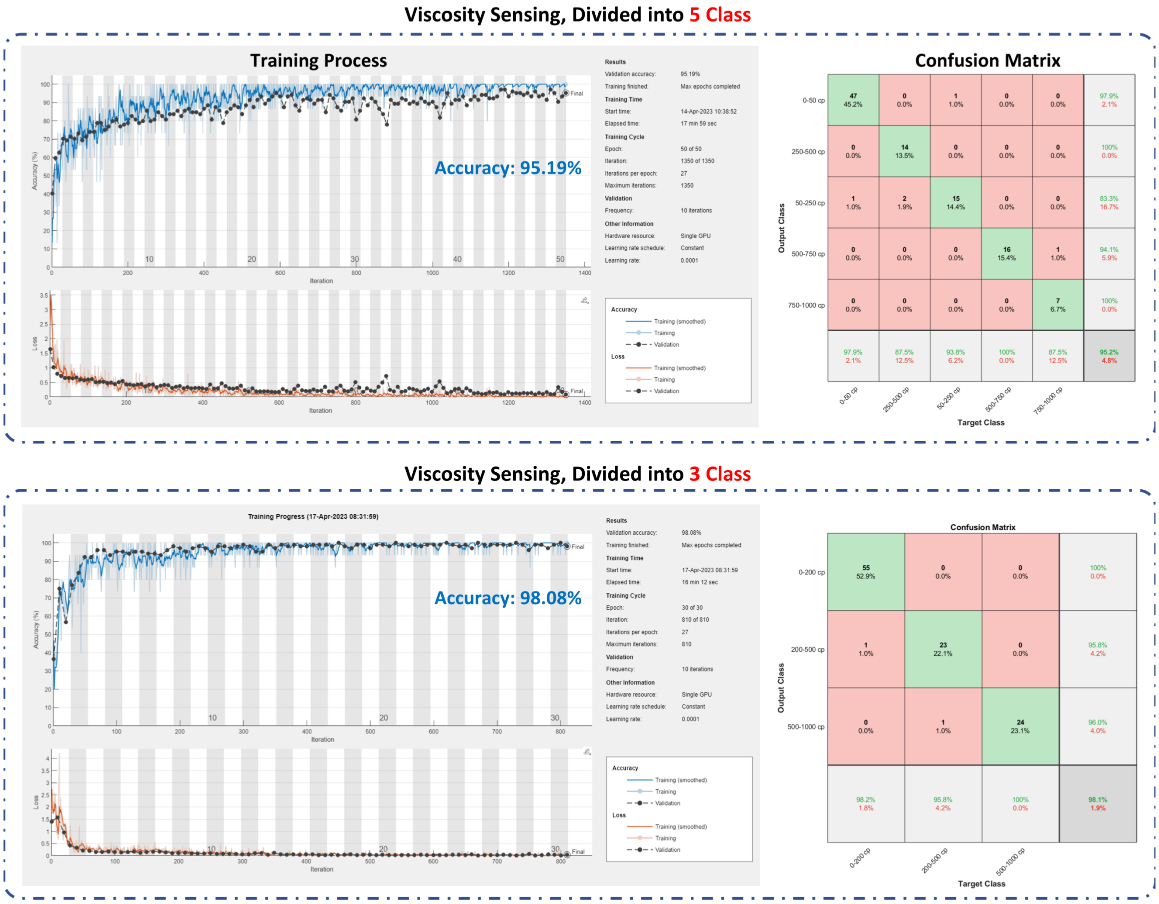

Fig. S23. Plot of the model training with different regrouped category size in viscosity sensing. (A) The plots of training process and confusion matrix of the dataset of 5 categories, which is regrouped from original dataset with 13 different viscosities. The validation accuracy is 95.19% at training iteration of 1,350. **(B)** The plots of training process and confusion matrix of the dataset of 3 categories, which is regrouped from original dataset with 13 different viscosities. The validation accuracy is 98.08% at training iteration of 810.

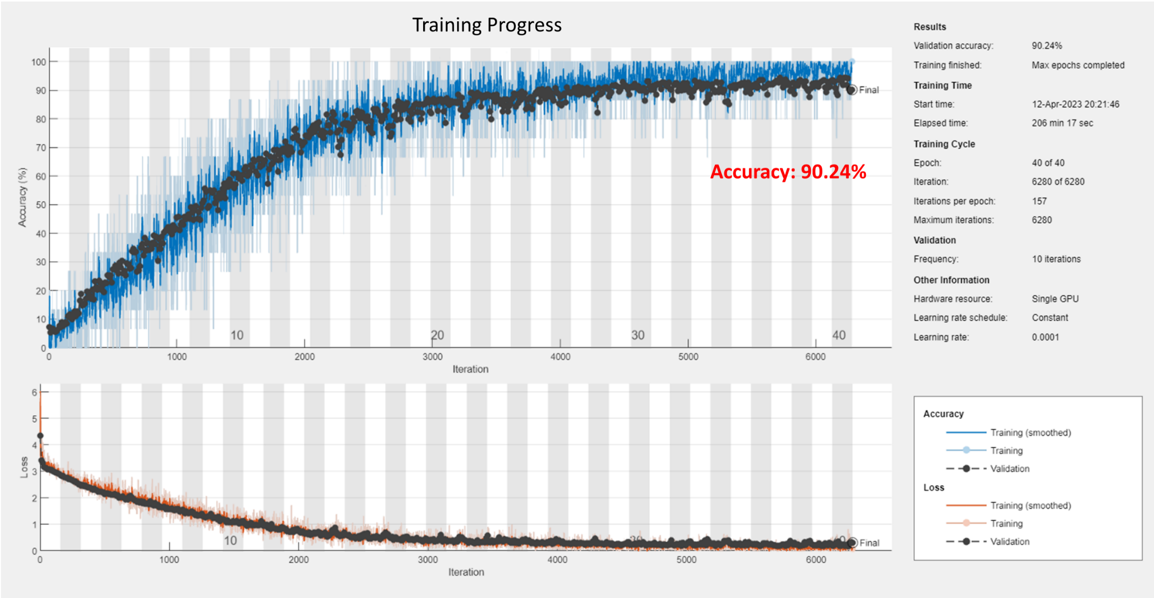

Fig. S24. Plot of the model training process for mixed situation with both viscosity and boundary condition changes. The training dataset includes 13 categories of different viscosity conditions with individual SICs of n = 40, and 15 categories of different boundary conditions with individual SICs of n = 160. The trained model with an iteration of 6,280 shows a validation accuracy of 90.24%.

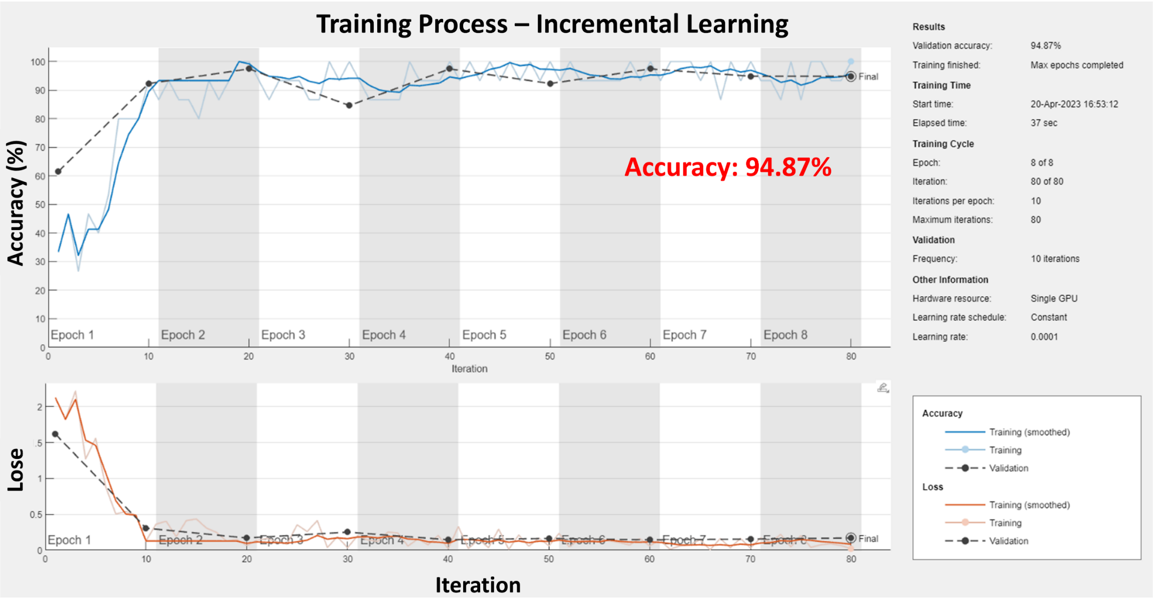

Fig. S25. training process of the incremental learning using trained model. The out-of-sample data with different boundary conditions (four categories of boundary distance, 200, 600, 1000, 1400 μm, and n = 40 in each category) were tested with the trained model for mixed sensing conditions. The validation accuracy reached its peak at around the 10th iteration and stabilized at 94.87%.

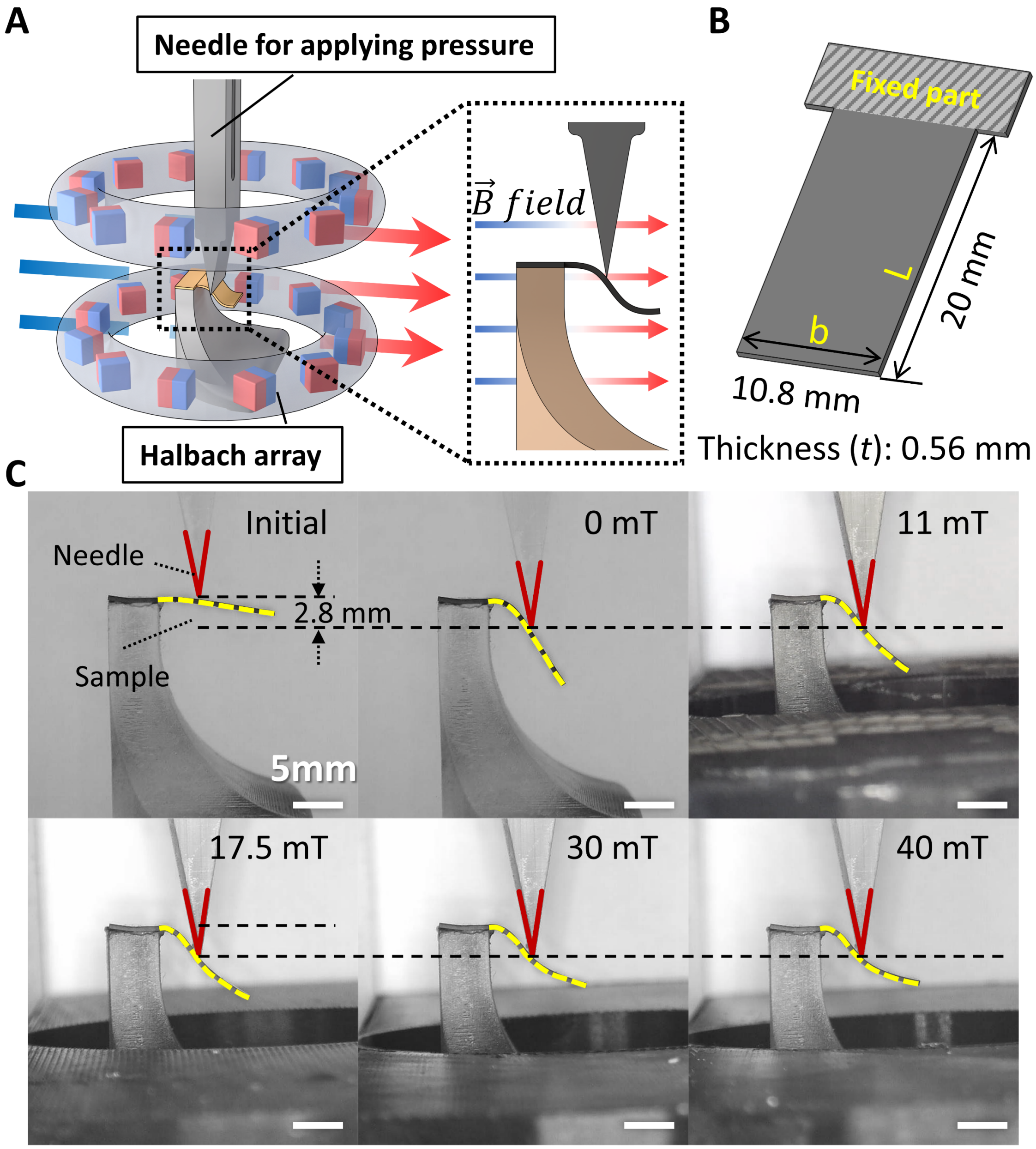

Fig. S26. Setup and experimental images of the magnetic field-enabled adjustable stiffness test. (A) The schematic of the setup for stiffness test under uniform magnetic field. The sample designed with same stiffness as the cilia used for actuation-enhanced sensing was mounted on the stand as a cantilever beam in uniform magnetic field with different strength, and a 3D printed tip mounted on a force sensor (Instron GmbH) was utilized to apply pressure to the sample in a quasi-static manner (speed of the tip, 0.01 mm/s). **(B)** The shape and parameters of the sample used in stiffness test ($20 \times 10.8 \times 0.56$ mm, length × width × thickness). **(C)** The experiment images of the sample before and after being pressed down by 2.8 mm. The uniform magnetic field was applied horizontally. Scale bar, 5 mm.

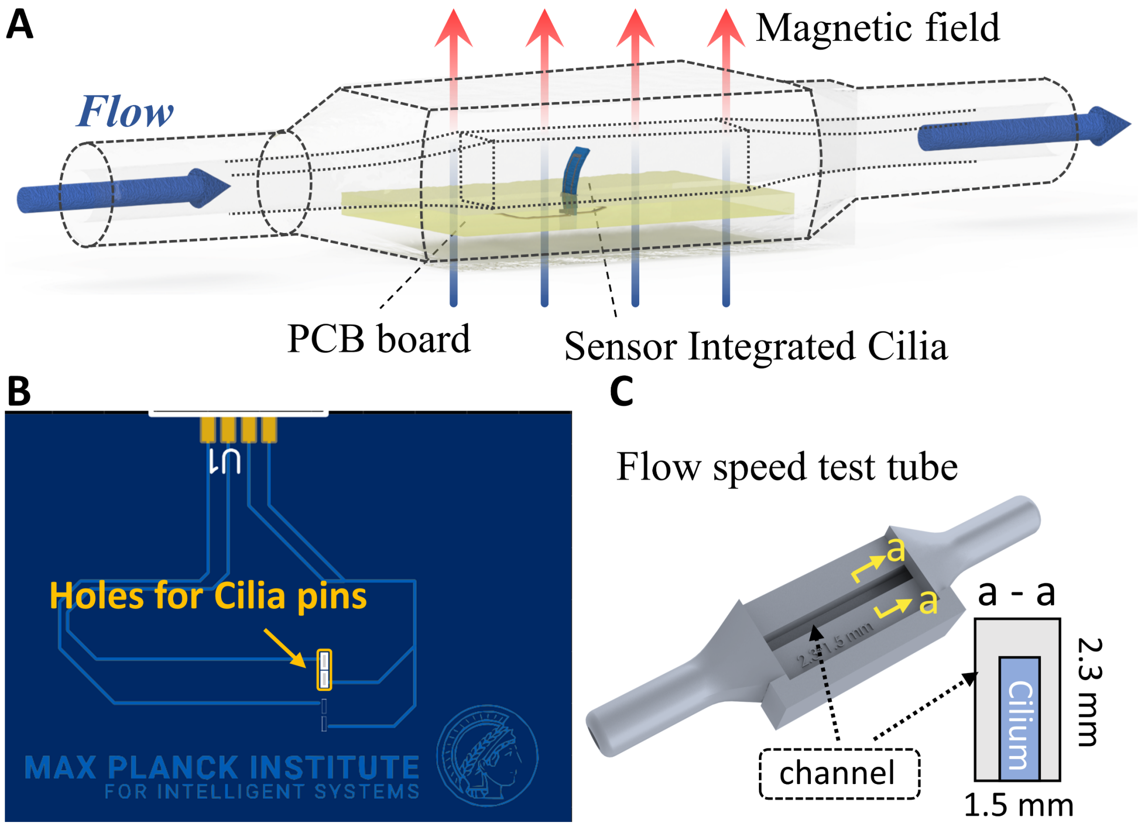

Fig. S27. Setup for flow sensing by the sensor integrated cilia array with adjustable stiffness. **(A)** The schematic of the setup for the sensor integrated cilia with adjustable stiffness in flow sensing. The SIC on PCB board was placed into a 3D printed cavity, and a uniform magnetic field was applied vertically, which along with the original direction of the cilium. Both ends the cavity were connected to the silicone hoses for the liquid flow in and out, and the flow speed was controlled by a syringe pump. **(B)** The PCB board designed for the data acquisition in flow speed sensing. **(C)** The design parameters of the channel of the cavity. Cross-sectional size, 1.5 mm × 2.3 mm (width by height).

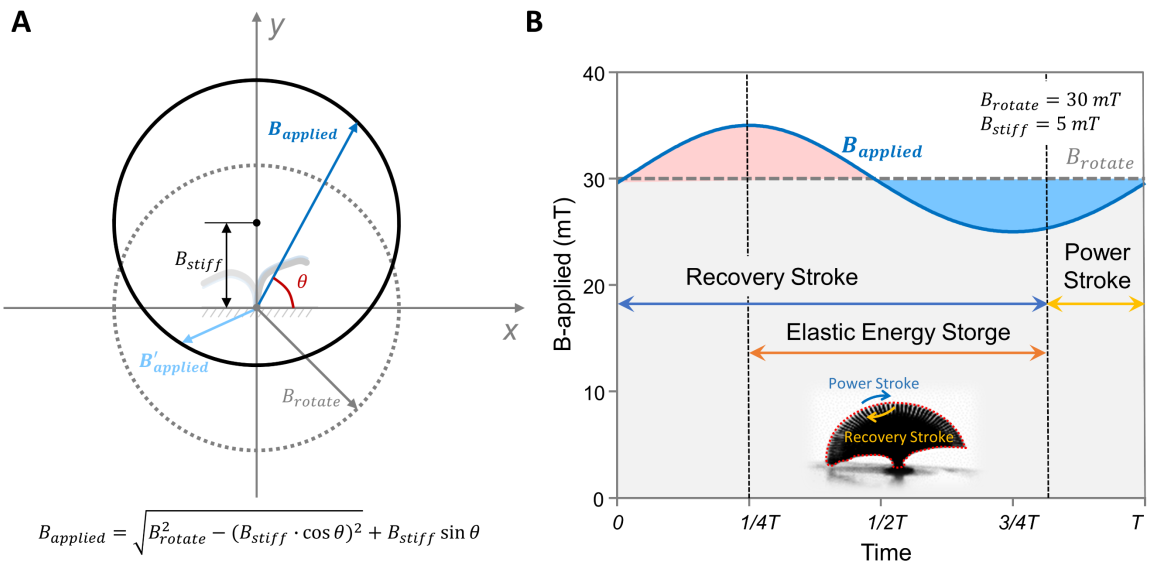

Figure S28. Schematic of the effect of time-varying intensity magnetic field on the movement of SIC. (A) A mixed magnetic field composed of a rotating uniform magnetic field and a y-direction static magnetic field. **(B)** The corresponding magnetic field strength for different states of ciliary movement.

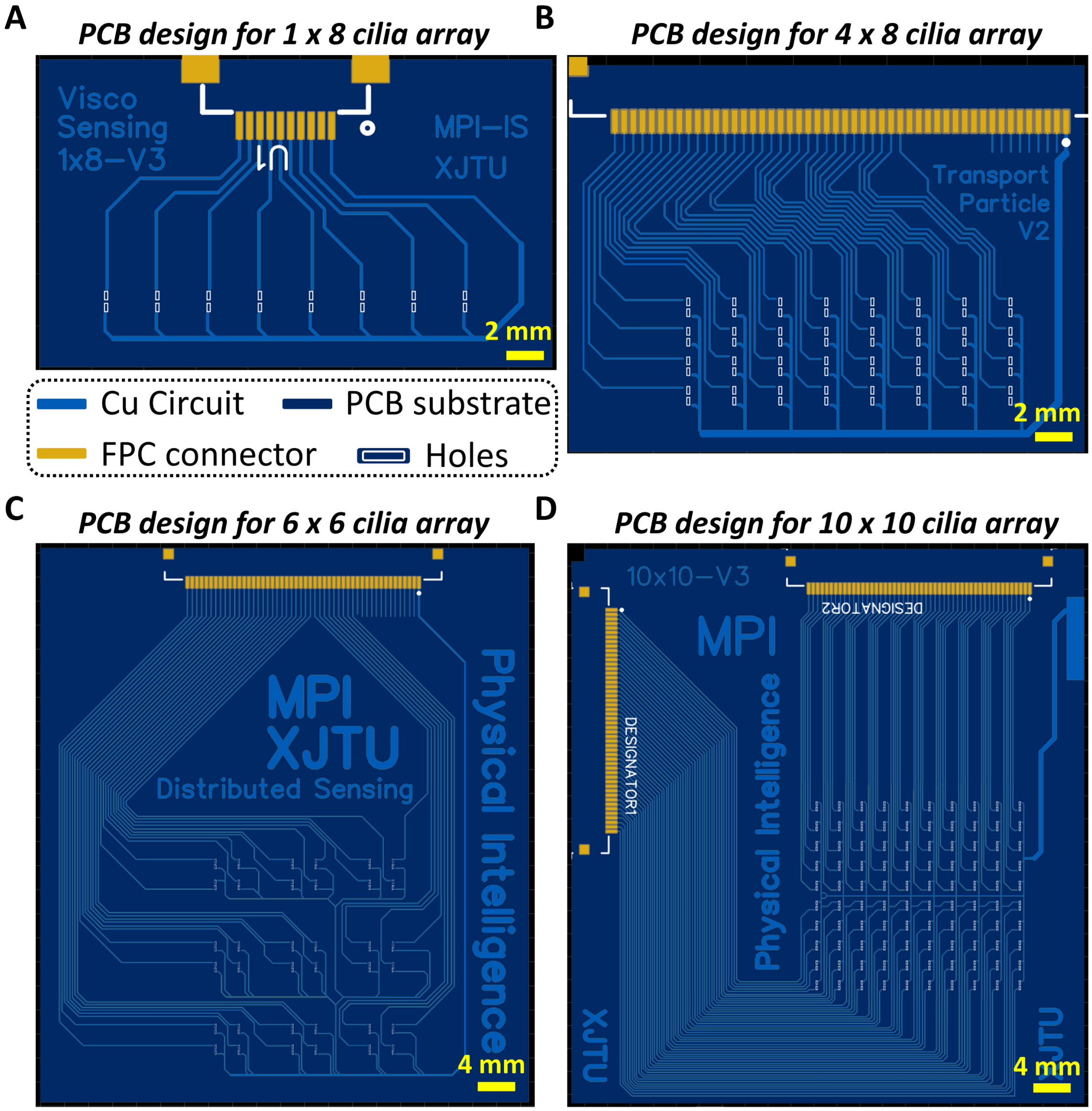

Fig. S29. PCB designs for data acquisition using SIC array with different array sizes. (A) The PCB designed for 1×8 cilia array. **(B)** The PCB designed for 4×8 cilia array. **(C)** The PCB designed for 6×6 cilia array. **(D)** The PCB designed for 10×10 cilia array.

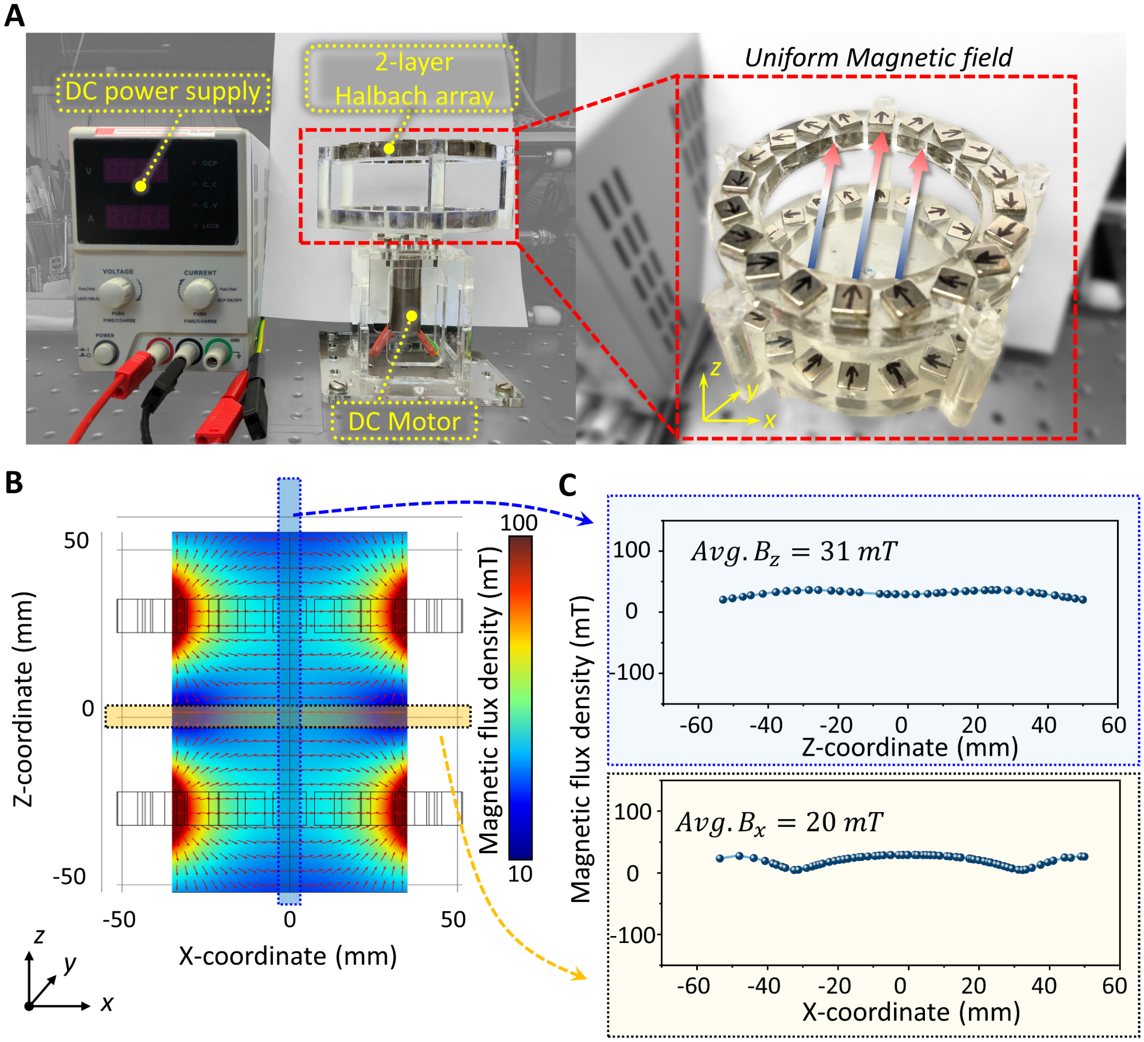

Fig. S30. Setup of the magnetic actuation system and the simulation results of the applied magnetic field. (A) An overview of the whole setup, including DC power supply (0-30 V, 0-5 A, RS PRO), DC motor (DCX brush gear motor, 12 V, 22.7 W, translation, 21:1, MAXON), two-layer Halbach array (20 magnetic cubes for each layer). **(B)** The distribution of the magnetic flux density inside the two-layer Halbach array. **(C)** The distribution of magnetic flux density along x- and y-axis in this two-layer Halbach array system.

Table S1. Comparison of cilia-like devices.

Materials	Stimulus	Actuation ability	Sensing ability	Sensing type	Sensing Method	Cilium Size (Length)	Ref
NiFe	Magnetic field	●	○	○	○	~400 µm	(8)
FePt JMP	Magnetic field	●	○	○	○	< 100 µm	(9)
Nickel particle	Magnetic field	●	○	○	○	~ 30 µm	(10)
NdFeB+elastomer	Magnetic field	●	○	○	○	Millimeter scale	(1, 11)
PPy	Electric field	●	○	○	○	Millimeter scale	(12)
Pt+Ti	Electric field	●	○	○	○	~50 µm	(13)
liquid crystal elastomer	light	●	○	○	○	150 µm – 1 mm	(14, 15)
PDMS	Pneumatic pump	●	○	○	○	Centimeter scale	(16)
PEG	Ultrasound	●	○	○	○	~100 µm	(17)

Materials	Stimulus	Actuation ability	Sensing ability	Sensing type	Sensing Method	Cilium Size (Length)	Ref
Hydrogel+PDMS+PVDF	○	○	●	Fluid flow	Resistance	< 1 mm	(18)
GNPs+PDMS/PUA	○	○	●	Air flow	Resistance	~ 300 μm	(19)
PDMS+iron nanowires/NdFeB	○	○	●	Fluid flow<br>tactile force	GMI	500 μm – 5 mm	(20, 21)
PDMS	○	○	●	Fluid viscosity	Visual Analysis	Millimeter scale	(22)
plastic	○	○	●	Fluid flow	Resistance	Millimeter scale	(23)
PDMS+Au+PE+Cu	○	○	●	vibration	Triboelectric voltage	< 1 mm	(24)
Hydrogel+CNT	○	○	●	Fluid flow	Piezoelectric voltage	Millimeter scale	(25)
**PDMS+LIG**	**Magnetic field**	●	●	**Fluid viscosity<br>Fluid boundary<br>Fluid flow**	**Resistance**	**Millimeter scale**	**Our work**

Table S2. Sizes of the sensor-integrated cilium.

	Length L (mm)	Width w (mm)	Thickness t (μm)
Cilium body	1.8	0.8	110
Sensor-sensing part	1.5	0.06	20
Sensor-electrode part	0.8	0.4	20

Table S3. Comparison of the pumping capability of artificial cilia in liquid environments.

Actuation Method	Scaled fluid velocity $v_{scaled} = \bar{v}_x/L$	Fluid Velocity $\bar{v}_x$	Cilia Length L	Actuation frequency f	Re	Reference
Magnetic	0.32	8 μm/s	25 μm	34 Hz	0.025	(26)
Magnetic	0.02	83 μm/s	4 mm	0.083 Hz	0.002 *	(11)
Electric	1.2	60 μm/s	50 μm	40 Hz	0.002	(13)
Electric	0.92	1.38 mm/s	1.5 mm	1.67 Hz	30	(12)
Acoustic	50	~ 5 mm/s	100 μm	20-100 kHz	31.4-62.8 (Re_f)	(17)
Light-driven	-	-	1 mm	0.04 Hz	-	(14)
Pneumatic	5.9e-5	0.83 μm/s	14-16 mm	0.125-0.5 Hz	0.02-0.04	(16, 27)
**This Work**	**0.39**	**0.7 mm/s**	**1.8 mm**	**2 Hz**	**0.03**	

* Estimated value

Table S4. Definition of the signal features in the time domain

**Time domain**	Minimum	$\mathbf{min}\ (\boldsymbol{x_i})$
	Maximum	$\mathbf{max}\ (\boldsymbol{x_i})$
	Mean	$\overline{\boldsymbol{X}} = \frac{\mathbf{1}}{\boldsymbol{N}}\sum_{\boldsymbol{i}=\mathbf{1}}^{\boldsymbol{N}} \boldsymbol{x_i}$
	Root mean square (RMS)	$\sqrt{\frac{\mathbf{1}}{\boldsymbol{N}}\sum_{\boldsymbol{I}=\mathbf{1}}^{\boldsymbol{N}} \boldsymbol{x_i^2}}$
	Variance	$\frac{\sum_{i=1}^{N}(\boldsymbol{x_i} - \overline{\boldsymbol{X}})^2}{\boldsymbol{N}}$
	Standard deviation	$\boldsymbol{\sigma} = \sqrt{\frac{\sum_{i=1}^{N}(\boldsymbol{x_i} - \overline{\boldsymbol{X}})^2}{\boldsymbol{N}}}$
	Power	$\frac{\mathbf{1}}{\boldsymbol{N}}\sum_{\boldsymbol{i}=\mathbf{1}}^{\boldsymbol{N}} \boldsymbol{x_i^2}$
	Peak	$\boldsymbol{P_m} = \mathbf{max}(\lvert\boldsymbol{x_i}\rvert)$
	Peak to Peak	$\boldsymbol{P_k} = \mathbf{max}(\boldsymbol{x_i}) - \mathbf{min}(\boldsymbol{x_i})$
	Crest factor	$\frac{\boldsymbol{P_m}}{\boldsymbol{RMS}}$
	Skewness	$\frac{\sum_{i=1}^{N}(\boldsymbol{x_i} - \overline{\boldsymbol{X}})^3}{\boldsymbol{N} \times \boldsymbol{\sigma}^3}$
	Kurtosis	$\frac{\sum_{i=1}^{N}(\boldsymbol{x_i} - \overline{\boldsymbol{X}})^4}{\boldsymbol{N} \times \boldsymbol{\sigma}^4}$

Table S5. Definition of the signal features in the frequency domain

**Frequency domain**	Maximum of band power spectrum	$\boldsymbol{S_{MAX} = \max(S(f)_i)}$
	Sum of total band power	$\boldsymbol{S_{SBP} = \sum_{i=1}^{N} S(f)_i}$
	Mean of band power spectrum	$\boldsymbol{S_{\mu} = \frac{1}{N}\sum_{i=1}^{N} S(f)_i}$
	Variance of band power	$\boldsymbol{S_V = \frac{\sum_{i=1}^{N}\left(S(f)_i - S_{\mu}\right)^2}{N}}$
	Peak of band power	$\boldsymbol{S_P = \max(\lvert S(f)_i \rvert)}$
	Skewness of band power	$\boldsymbol{S_S = \frac{1}{N}\frac{\sum_{i=1}^{N}\left(S(f)_i - S_{\mu}\right)^3}{S_V^{3/2}}}$
	Kurtosis of band power	$\boldsymbol{S_K = \frac{1}{N}\frac{\sum_{i=1}^{N}\left(S(f)_i - S_{\mu}\right)^4}{S_V^{4/2}}}$

Table S6. Comparison of the actuation frequency of magnetic cilia and cilia-like magnetic robots under liquid environment.

Type	Typical size	Actuation Frequency	Liquid Environment	Reference
Magnetic cilia	$\phi 50\mu m$, $L = 350\mu m$	40 Hz	Water	(28)
Magnetic cilia	$\phi 50\mu m$, $L = 350\mu m$	100 Hz	Water	(29)
Magnetic cilia	1μm × 1μm × 10μm	100 Hz	Water	(9)
Magnetic cilia	$\phi 2\mu m$, $L = 23\mu m$	80 Hz	Ethanol and Water	(30)
Cilia like magnetic robots	$L_{beam} = 1.5mm$ Thickness=65μm	30 Hz	Water	(31)
Magnetic Cilia	$L_{beam} = 1.8mm$ Thickness=120μm	7 Hz	Glycerol	**This Work**

Table S7. Classification accuracy of the boundary distance sensing using LDA with different number of features (boundary size, 10 mm; actuation frequency, 2 Hz).

Number of Features*	mean of accuracy**	std of accuracy
1	0.783816	0.081778
2	0.954408	0.038047
3	0.977234	0.024082
4	0.975725	0.024023
5	0.982729	0.026451
6	0.979952	0.028766
7	0.978502	0.028814
8	0.981341	0.024116
9	0.985628	0.020329
10	0.985628	0.020329
11	0.985628	0.020329
12	0.979891	0.021503
13	0.979952	0.026485
14	0.979952	0.026485
15	0.979952	0.026485

* The number of signal features that occupied in the signal classification.
** The accuracy is dimensionless. The range of accuracy is from 0 to 1.

Table S8. The essential parameters used in model training.

Name of parameter	Essential parameters
GoogLeNet	
Input image size	224 by 224 by 3
splitEachLabel	0.8
InitialLearnRate	0.0001
LearnRateSchedule	None
LearnRateDropFactor	0.5
WeightLearnRateFactor	16
BiasLearnRateFactor	10
MiniBatchSize	15
MaxEpochs	30-50
ValidationFrequency	10
Verbose	1

Legends for Movie S1-S7

Movie S1. Flow control and particle transportation at low Reynolds number by the SIC array with magnetically controlled metachronal coordination. This video shows our proposed sensor integrated cilia array can pump the flow to desired direction and transport the particles at low Reynolds number environment (glycerol) by changing the rotational direction of the applied magnetic field.

Movie S2. The sensing mechanism of the proposed SIC array with metachronal coordination. This video shows the mechanism of the sensor-integrated artificial cilia including the active motion driven by rotating magnetic field and the sensing of cilia deflection using the integrated LIG-based strain gauge sensor.

Movie S3. Magnetically actuated motion with second-order oscillation of the SIC array during the power stroke in the air. This video shows the movement of second-order oscillation of the sensor integrated cilia when actuated in the atmospheric environment, revealed in both the recorded video and the saved signals. The video is taken by a high-speed camera with a frame rate of $7{,}700$ frame per second (fps).

Movie S4. Simultaneous and distributed viscosity sensing and liquid mixing by the SIC array. This video shows the recorded videos, raw signals and predicted viscosities of our proposed sensor integrated cilia array, which can simultaneously sense the viscosity of the liquid environment and mixing the liquids with different viscosities. The distributed sensing ability also shows advantage in detecting the dynamic changes in viscosity in localized areas.

Movie S5. Viscosity sensing with the SIC array. This video shows the schematic of the proposed viscosity sensing mechanism, the recorded videos and raw signals during the experiment under liquid environment with different viscosities. Both the videos and signals clearly show that the amplitude in both the power stroke and the recovery stroke is decreasing as viscosity increased.

Movie S6. Boundary sensing with the SIC array. This video shows the schematic of the proposed boundary sensing mechanism, the recorded videos and raw signals during the experiment with different boundary distance. The liquid we used is glycerol with viscosity of $987.6\ \mathrm{cP}$.

Movie S7. Sensing environmental fluid flows with reconfigurable sensing ranges and sensitivity. This video shows the comparison of deformation and recorded signal of the SIC between different strength of magnetic field in same liquid flow. The cilium with larger applied magnetic field shows smaller shape deformation and more stable signal, which reveals the superiority of tuneable stiffness in sensing extreme flows. The liquid we used is simulated blood that prepared by a mixed solution of glycerol and deionized water (volume ratio of water to glycerol, $65:44$; viscosity, $4.26\ \mathrm{cP}$).

SI References

1. X. Dong *et al.*, Bioinspired cilia arrays with programmable nonreciprocal motion and metachronal coordination. *Science advances* **6**, eabc9323 (2020).
2. L. Johnston, J. Yang, J. Han, K. Kalantar-Zadeh, J. Tang, Intermetallic wetting enabled high resolution liquid metal patterning for 3D and flexible electronics. *Journal of Materials Chemistry C* **10**, 921-931 (2022).
3. F. Pedregosa *et al.*, Scikit-learn: Machine learning in Python. *the Journal of machine Learning research* **12**, 2825-2830 (2011).
4. C. R. Harris *et al.*, Array programming with NumPy. *Nature* **585**, 357-362 (2020).
5. G. Bradski, The openCV library. *Dr. Dobb's Journal: Software Tools for the Professional Programmer* **25**, 120-123 (2000).
6. J. Schindelin *et al.*, Fiji: an open-source platform for biological-image analysis. *Nature methods* **9**, 676-682 (2012).
7. S. Hanasoge, P. J. Hesketh, A. Alexeev, Metachronal motion of artificial magnetic cilia. *Soft Matter* **14**, 3689-3693 (2018).
8. S. Hanasoge, M. Ballard, P. J. Hesketh, A. Alexeev, Asymmetric motion of magnetically actuated artificial cilia. *Lab on a Chip* **17**, 3138-3145 (2017).
9. S. Zhang *et al.*, 3D-printed micrometer-scale wireless magnetic cilia with metachronal programmability. *Science Advances* **9**, eadf9462 (2023).
10. M. Vilfan *et al.*, Self-assembled artificial cilia. *Proceedings of the national academy of sciences* **107**, 1844-1847 (2010).
11. H. Gu *et al.*, Magnetic cilia carpets with programmable metachronal waves. *Nature communications* **11**, 2637 (2020).
12. Z. Ren *et al.*, Soft-robotic ciliated epidermis for reconfigurable coordinated fluid manipulation. *Science Advances* **8**, eabq2345 (2022).
13. W. Wang *et al.*, Cilia metasurfaces for electronically programmable microfluidic manipulation. *Nature* **605**, 681-686 (2022).
14. C. L. Van Oosten, C. W. Bastiaansen, D. J. Broer, Printed artificial cilia from liquid-crystal network actuators modularly driven by light. *Nature materials* **8**, 677-682 (2009).
15. S. Li *et al.*, Self-regulated non-reciprocal motions in single-material microstructures. *Nature* **605**, 76-83 (2022).
16. E. Milana *et al.*, Metachronal patterns in artificial cilia for low Reynolds number fluid propulsion. *Science advances* **6**, eabd2508 (2020).
17. C. Dillinger, N. Nama, D. Ahmed, Ultrasound-activated ciliary bands for microrobotic systems inspired by starfish. *Nature communications* **12**, 6455 (2021).
18. M. Asadnia *et al.*, From biological cilia to artificial flow sensors: Biomimetic soft polymer nanosensors with high sensing performance. *Scientific reports* **6**, 32955 (2016).
19. S. Chun *et al.*, Bioinspired hairy skin electronics for detecting the direction and incident angle of airflow. *ACS applied materials & interfaces* **11**, 13608-13615 (2019).
20. A. Alfadhel, J. Kosel, Magnetic Nanocomposite Cilia Tactile Sensor. *Advanced Materials* **27**, 7888-7892 (2015).
21. J. Man, J. Zhang, G. Chen, N. Xue, J. Chen, A tactile and airflow motion sensor based on flexible double-layer magnetic cilia. *Microsystems & Nanoengineering* **9**, 12 (2023).
22. A. Mustafa *et al.*, A micropillar-based microfluidic viscometer for Newtonian and non-Newtonian fluids. *Analytica Chimica Acta* **1135**, 107-115 (2020).
23. S. Yang *et al.*, High-sensitivity lollipop-shaped cilia sensor for ocean turbulence measurement. *Sensors and Actuators A: Physical* **332**, 113109 (2021).
24. Q. He *et al.*, Triboelectric vibration sensor for a human-machine interface built on ubiquitous surfaces. *Nano Energy* **59**, 689-696 (2019).
25. A. G. P. Kottapalli, M. Bora, D. Sengupta, J. Miao, M. S. Triantafyllou (2018) Hydrogel-CNT biomimetic cilia for flow sensing. in *2018 IEEE SENSORS* (IEEE), pp 1-4.
26. A. Shields *et al.*, Biomimetic cilia arrays generate simultaneous pumping and mixing regimes. *Proceedings of the National Academy of Sciences* **107**, 15670-15675 (2010).

27. E. Milana, B. Gorissen, S. Peerlinck, M. De Volder, D. Reynaerts, Artificial soft cilia with asymmetric beating patterns for biomimetic low‐Reynolds‐number fluid propulsion. *Advanced Functional Materials* **29**, 1900462 (2019).
28. S. Zhang, Y. Wang, P. R. Onck, J. M. den Toonder, Removal of microparticles by ciliated surfaces—An experimental study. *Advanced Functional Materials* **29**, 1806434 (2019).
29. S. Zhang, Z. Cui, Y. Wang, J. M. den Toonder, Metachronal actuation of microscopic magnetic artificial cilia generates strong microfluidic pumping. *Lab on a Chip* **20**, 3569-3581 (2020).
30. T. u. Islam, Y. Bellouard, J. M. den Toonder, Highly motile nanoscale magnetic artificial cilia. *Proceedings of the National Academy of Sciences* **118**, e2104930118 (2021).
31. Z. Ren, W. Hu, X. Dong, M. Sitti, Multi-functional soft-bodied jellyfish-like swimming. *Nature communications* **10**, 2703 (2019).
